# Evaluation of the Accuracy of Contactless Consumer Sleep-Tracking Devices Application in Human Experiment: A Systematic Review and Meta-Analysis

**DOI:** 10.3390/s23104842

**Published:** 2023-05-17

**Authors:** Huifang Zhai, Yonghong Yan, Siqi He, Pinyong Zhao, Bohan Zhang

**Affiliations:** 1 Faculty of Architecture and Urban Planning, Chongqing University, Chongqing 400044, China; zhaihuifang@cqu.edu.cn; 2 Key Laboratory of New Technology for Construction of Cities in Mountain Area, Chongqing University, Chongqing 400044, China; 3 College of Landscape Architecture, Nanjing Forestry University, Nanjing 210037, China; 4 College of Mathematics and Statistics, Chongqing University, Chongqing 400044, China; 5 Faculty of Engineering, The University of Sydney, Camperdown, NSW 2006, Australia

**Keywords:** polysomnography, actigraphy, sleep stages, validation

## Abstract

Compared with the gold standard, polysomnography (PSG), and silver standard, actigraphy, contactless consumer sleep-tracking devices (CCSTDs) are more advantageous for implementing large-sample and long-period experiments in the field and out of the laboratory due to their low price, convenience, and unobtrusiveness. This review aimed to examine the effectiveness of CCSTDs application in human experiments. A systematic review and meta-analysis (PRISMA) of their performance in monitoring sleep parameters were conducted (PROSPERO: CRD42022342378). PubMed, EMBASE, Cochrane CENTRALE, and Web of Science were searched, and 26 articles were qualified for systematic review, of which 22 provided quantitative data for meta-analysis. The findings show that CCSTDs had a better accuracy in the experimental group of healthy participants who wore mattress-based devices with piezoelectric sensors. CCSTDs’ performance in distinguishing waking from sleeping epochs is as good as that of actigraphy. Moreover, CCSTDs provide data on sleep stages that are not available when actigraphy is used. Therefore, CCSTDs could be an effective alternative tool to PSG and actigraphy in human experiments.

## 1. Introduction

Sleep is an important indicator when analyzing fatigue [1,2], working performance [3,4], mood [5,6], circadian entrainment [7,8], etc. Some human experiments om large samples have been conducted in the field, with sleep monitoring performed at home [6,9,10]. Although the gold standard for measuring sleep, polysomnography (PSG), is the most precise device used to evaluate sleep quality, it is expensive and requires the assistance of specialists [11]. In addition, the discomfort caused by electrodes attached to the human body can interfere with normal sleep behavior, making it impossible to monitor long-term sleep in home environments [12]. Currently, actigraphy has become a widely used instrument for assessing sleep in human experiments [1,13,14,15]. Compared with PSG, actigraphy provides a convenient and feasible solution for long-term sleep monitoring in the home environment. However, actigraphy is still costly to use in sleep experiments with a large sample size as it requires simultaneity. Moreover, as a wearable device, some people feel uncomfortable and easily forget to wear them before sleep [16].

Contactless consumer sleep-tracking devices (hereinafter referred to as ‘CCSTDs’) have a low cost and are simple to operate. Whether it is a bedside device using technologies, such as radiofrequency identification, an infrared camera, or a mattress device that employs pressure and piezoelectric sensors, they make minimal or zero contact with the human body, minimizing interference with the user’s natural sleep behavior [17]. Studies on the accuracy, validity, and reliability of different brands of CCSTDs have been extensively conducted on different groups, including psychiatric patients [18], burn patients, rehabilitation patients [19], ICU patients [20], autistic children [21], dementia patients [22], elderly patients [22,23,24], and infants [25]. The findings suggest that CCSTDs have great advantages and potential for application in large-sample and long-period sleep experiments. The objective of this study is to evaluate whether CCSTDs can be widely used as a valid alternative tool to PSG and actigraphy in human experiments by assessing the accuracy and performance of CCSTDs with a systematic review of relevant publications and a meta-analysis of their reported data.

## 2. Method

This systematic review was conducted following the Preferred Reporting Items for Systematic Reviews and Meta-Analyses (PRISMA) statement www.prisma-statement.org (accessed on 2 April 2022). The protocol for this review was registered in the International Prospective Register of Systematic Reviews (PROSPERO: CRD42022342378, which is available at: https://www.crd.york.ac.uk/prospero/display_record.php?ID=CRD42022342378, accessed on 2 April 2022).

### 2.1. Search Strategy

An online comprehensive search of the following databases was performed between April and August 2022: PubMed, EMBASE, Cochrane CENTRALE, and Web of Science.

The search was designed to find studies of CCSTDs that involved the validation of sleep outcomes using the gold standard, PSG. The following terms (including synonyms and medical subject headings terms) were used for the search: “sleep” and “contactless” or “brand name (e.g., Emfit, Beddit, ResMed, etc.)” and “polysomnography” (Mesh). The search was performed without date, language, or publication status restrictions. The search strategies for each database can be found in the Supplementary Materials Table S1.

### 2.2. Eligibility Criteria

Literature eligible for inclusion in the meta-analysis was as follows: (1) contactless sleep monitoring products were commercially available and of consumer grade. (2) One or more of the following outcomes were reported: TST, total sleep time; WASO, wake after sleep onset; SOL, sleep onset latency; SE, sleep efficiency; light sleep (N1 + N2); deep sleep (N3); REM, rapid eye movement; sensitivity, specificity, and accuracy in detecting sleep epochs; (3) sleep data from PSG or professional sleep monitoring instruments with equivalent validity were used as the reference standard; (4) the experimental subjects were human.

Exclusion criteria included (1) fewer than five subjects, (2) conference papers, case series, and case reports, (3) absent or inappropriate statistical analysis, and (4) sleep tracking apps on smartphones.

### 2.3. Data Extraction

Characteristics for each study were extracted and collated in a standardized table containing the first author, year of publication, sample characteristics (sample size, age, health status, and country), sleep device characteristics (name, sensor, and placement location), reference, sleep site, duration of the assessment, and bedtime. The study outcomes relative to the denoted reference standard, total sleep time (TST), sleep onset latency (SOL), wake after sleep onset (WASO), sleep efficiency (SE), light sleep, deep sleep, and rapid eye movement (REM), as well as the sensitivity, specificity, and accuracy of detecting sleep epochs, were extracted. Endpoints reported as median and range were transformed into mean and standard deviation using the method of Hozo et al. [26].

### 2.4. Bias Assessment

The studies’ quality was assessed (unblinded) using the standards of the quality assessment of diagnostic accuracy studies, version 2 (QUADAS-2) [27] (see Supplementary Materials (Figures S1 and S2)).

### 2.5. Statistical Analysis

For studies that compared CCSTDs with PSG and provided quantitative data, the mean, standard deviation, and sample size were used to produce a pooled estimated mean effect size and 95% confidence intervals (CI) using a random effects model. Heterogeneity among studies was determined by calculating I^2^ statistics. I^2^ values in the order of 25%, 50%, and 75% were considered as having small, medium, and large heterogenicity, respectively [28]. A threshold probability of 5% (*p* = 0.05) was selected as the basis for rejecting the null hypothesis. As recommended, the threshold probability of 10% (*p* = 0.10) was the basis for testing the significance of heterogenicity, and also, for determining the statistical significance of subgroup comparisons [29]. Meta-analyses were performed using Stata version 14, with metan, metafunnel, and metabias packages. In addition, Review Manager 5.4.1 and R program were used for analysis in this study.

## 3. Results

### 3.1. Study Selection

**Figure 1:**
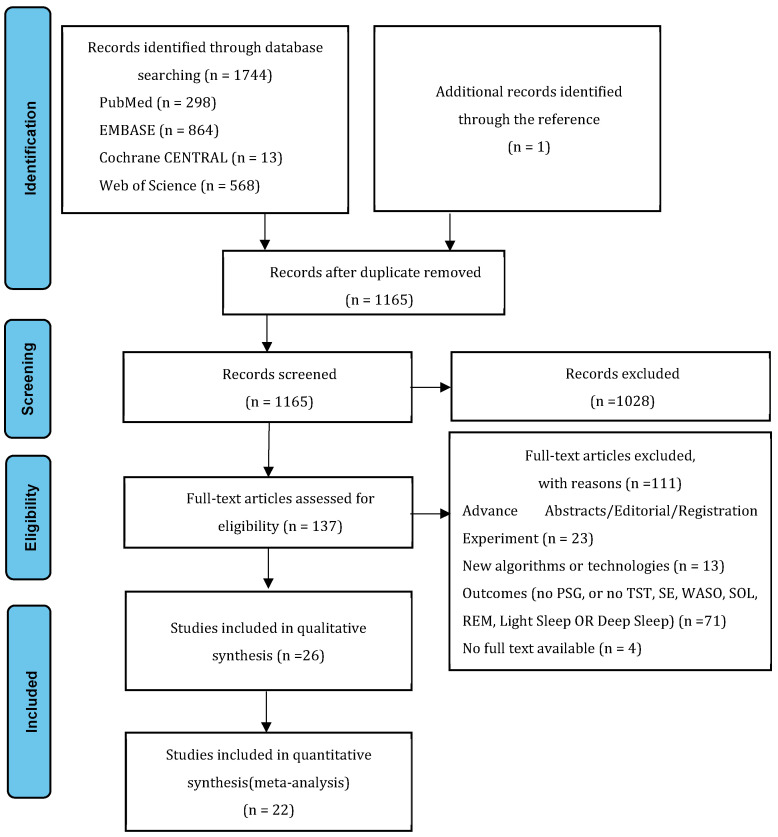
PRISMA flow diagram.

Figure 1 presents a summary of the selection and qualification of articles that were reviewed. A total of 1744 publications were retrieved through searching the databases. An additional publication was identified in the reference list of identified articles. After removing duplicate publications found in multiple databases, 1165 articles remained for screening. The examination of individual titles and abstracts yielded 137 publications for the full-text appraisal. However, after scrutinizing each of them according to a priori inclusion and exclusion criteria, only 26 articles were qualified for systematic review [17,20,30,31,32,33,34,35,36,37,38,39,40,41,42,43,44,45,46,47,48,49,50,51,52,53]. Out of the 26 articles, one did not evaluate PSG and CCSTD simultaneously [30], which likely led to a bias; three did not include data of meta-analysis [30,39,41,46]. Finally, 22 articles qualified for meta-analysis [17,20,31,32,33,34,35,36,37,38,40,42,43,44,45,47,48,49,50,51,52,53].

### 3.2. Main Characteristics of the Studies Included in the Review

**Figure 2:**
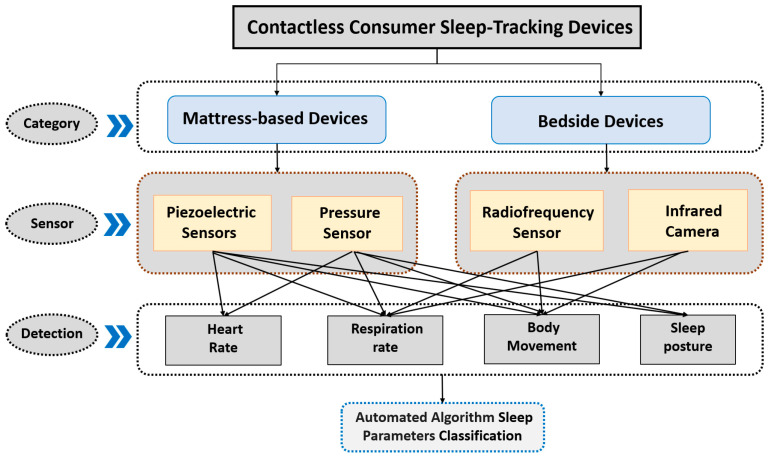
Types of CCSTDs and contactless sleep monitoring mechanism.

The descriptive characteristics of 26 included articles are summarized in Table 1, and the results from qualified publications on CCSTDs and actigraphy versus PSG can be found in the Supplementary Materials Table S2. These articles described 32 studies of CCSTDs. Among the studies that used PSG as the standard test, five studies compared CCSTDs and actigraphy using the same cohort [34,35,42,49,53]. Their performance was tested against that of PSG by making participants wear both CCSTD and an actigraphy device. CCSTDs use two types of device, i.e., mattress-based devices and bedside devices. Out of 26 articles, 11 (42%) studied mattress-based devices, 12 (46%) studied bedside devices, 2 (8%) studied both mattress-based and bedside devices [49,51], and 1 (4%) studied bedside an automatic video device [36]. The most common device sensors used in CCSTDs were piezoelectric sensors (n = 9), pressure sensors (n = 5), radiofrequency sensors (n = 17), and infrared cameras (n = 1). Figure 2 shows the different types of sensors and their sleep monitoring mechanism. Piezoelectric or pressure sensors are used in mattress-based devices, and radiofrequency sensors and infrared cameras are used in bedside devices. These sensors could monitor two or four physiological signals in real time, including heart rate, respitation rate, body movement, and sleep posture, and data were analyzed to determine the sleep status via built-in algorithms. These studies were performed in 11 countries. The number of participants ranged from 5 to 198, and they aged from 16 to 84 years. The participants included normal sleepers and those diagnosed with sleep (PLMS), obstructive sleep apnea (OSA), sleep-disordered breathing (SDB), central disorders of hypersomnolence (CDH), insomnia, diabetes, hypertension, arthritis, perioperative, and septic shock, according to periodic limb movements. Feng et al. [45] was included in the patient group in the meta-analysis since only one of the subjects did not have OSA. Among twenty-six articles, experiments in twenty-one articles (81%) were conducted in sleep laboratories, one (4%) was conducted in the home environment, three (11%) were conducted either at home or in a sleep laboratory, and one was conducted (4%) in an ICU [20].

### 3.3. Publication Bias

According to the Cochrane handbook, a publication bias test was performed on the outcome indicators included in the meta-analysis for more than ten studies. The Funnel plot showed no publication bias (see Supplementary Materials Figure S3). Egger’s test (P_TST_ = 0.608, P_SOL_ = 0.595, P_SE_ = 0.458, P_WASO_ = 0.637, P_lightsleep_ = 0.172, P_deepsleep_ = 0.997, and P_REM_ = 0.487) and Begg’s test (P_TST_ = 0.733, P_SOL_ = 0.434, P_SE_ = 0.707, P_WASO_ = 0.620, P_lightsleep_ = 0.371, P_deepsleep_ = 0.721, and P_REM_ = 0.592) indicated no publication bias.

### 3.4. Meta-Analysis on Accuracy of Sleep Parameters Assessed Using CCSTDs Versus PSG

#### 3.4.1. Principal Analysis

Out of the 26 articles, 22 articles were included in the meta-analysis. Of the four articles excluded, three articles reported their results without raw data, and in one article, the CCSTD was not monitored at the same time as the gold standard, PSG, in the experiment [30], which likely led to a bias. The articles included described thirty-six studies in total, with six articles each reporting from two to five studies of CCSTDs. A summary of the values for each outcome is provided in Table 2. The pooled estimate of effect size reveals the following results on CCSTDs relative to PSG: significant overestimations of TST (N = 35 studies; pooled mean = 19.55 min, 95% CI from 12.22 to 26.88; *p* < 0.001), SE (N = 28 studies; pooled mean = 2.88%, 95% CI from 1.58% to 4.17%; *p* < 0.01), deep sleep (N = 10 studies; pooled mean = 11.07 min, 95% CI from 0.37 to 21.76; *p* < 0.05); significant underestimation of SOL (N = 17 studies; pooled mean = −4.61 min, 95% CI from −6.56 to −2.66; *p* < 0.01), WASO (N = 16 studies; pooled mean = −12.07 min, 95% CI from −18.75 to −5.38; *p* < 0.01); non-significant differences in the estimation of light sleep (N = 10 studies; pooled mean = 5.62 min, 95% CI from −12.81 to 24.06; *p* = 0.550) and REM (N = 10 studies; pooled mean = 3.44 min, 95% CI from −14.81, 21.68; *p* = 0.823). All outcome indexes showed medium and large degrees of heterogeneity, except SE (I^2^ = 34.2%, *p* = 0.041).

#### 3.4.2. Subgroup Analyses

The results of subgroup analyses of sensors, device type, participant type, and brands compared with those of PSG are presented in the Supplementary Materials Tables S3–S6 and Figures S4–S31.

The subgroup analyses of sensors reveal that there were no significant differences for the piezoelectric sensor in the estimation of TST, SOL, WASO, and REM; for the pressure sensor in the estimation of SOL, SE, and deep sleep; for the radiofrequency sensor in the estimation of sleep stages; for infrared camera in the estimation of TST. In the subgroup of device types, there were no significant differences for the mattress-based devices in the estimation of SOL, WASO, and sleep stages or for the bedside devices in the estimation of sleep stages. In the subgroup of participant types, there were no significant differences among healthy participants in assessing sleep stages; for patient who participated in REM studies; for healthy and patient who participated in WASO and deep sleep studies. The results of the subgroup analyses for the brand of devices showed no significant differences for the use of ResMed S+ in terms of all the sleep parameters; for the use of Beddit in terms of SOL, WASO, and SE; for the use of Sonomat in terms of TST; for the use of other brands of mattress devices in terms of SOL, SE, and sleep stages; for the use of other brands of bedside devices in terms of TST, SOL, SE, light sleep, and REM.

### 3.5. Accuracy, Sensitivity, and Specificity in Detecting Sleep Epochs by CCSTDs Versus PSG

#### 3.5.1. Sleep and Wake Epoch Identification

**Figure 3:**
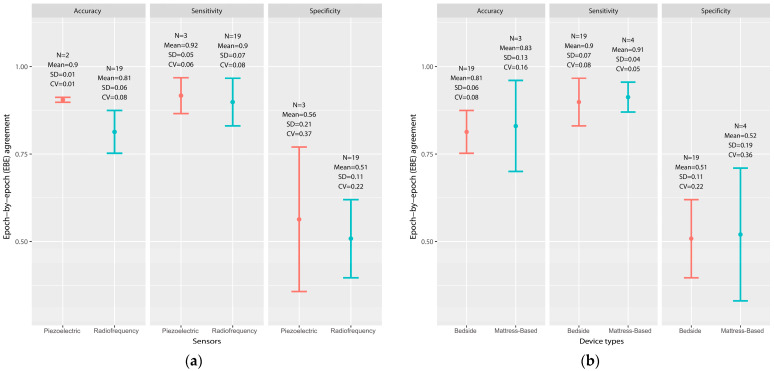

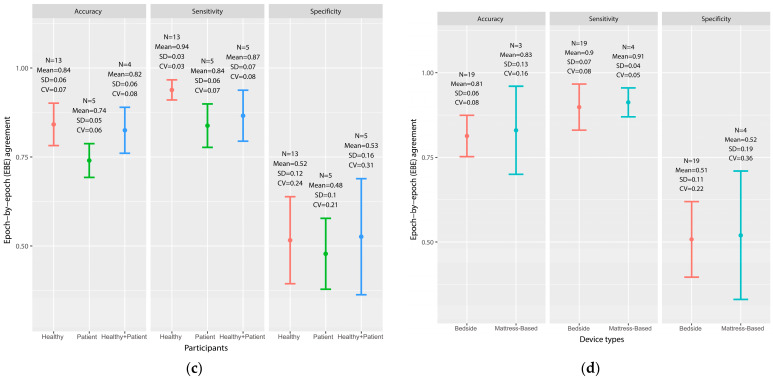
Comparison of the epoch-by-epoch (EBE) agreement for asleep/awake subgroups using error bars. (**a**) Sensors, (**b**) device types, (**c**) participants, and (**d**) brands (the number of samples of the same brand greater than or equal to two will be included in the same group, and the other samples are divided into (1) other brands of bedside device, and (2) other brands of mattress device). N, number of studies; CV, coefficient of variation; bedside, bedside devices; mattress-based, mattress-based devices; healthy, healthy participants; healthy + patient, both healthy and patient participants; others(M), other brands of mattress device; others(B), other brands of bedside device; accuracy, proportion of correctly classified sleep and wake epochs; sensitivity, proportion of correctly classified sleep epochs; specificity, proportion of correctly classified wake epochs [54].

A total of twelve studies (n = 1200 samples) involved an epoch-by-epoch (EBE) investigation of CCSTDs, taking PSG as a reference for sleeping and awake state determination [20,31,32,34,35,39,40,41,42,43,48,49]. Out of these twelve studies, four consisted of two or five different samples, thereby increasing the total number of evaluations to twenty-four. Across those trials, CCSTDs versus PSG analyses identified sleep epochs with accuracy values between 0.68 and 0.91 (0.81 ± 0.07) (N = 22 studies), sensitivity values between 0.75 and 0.97 (0.90 ± 0.06) (N = 23 studies), and specificity values between 0.37 and 0.80 (0.51 ± 0.12) (N = 23 studies). Figure 3 displays error bars of the EBE agreement for sleeping and awake states in terms of sensors, the device type, the health conditions of the participants, and the brand of device. As only one study [20] provided EBE data given by the pressure sensor, it was not included in the error bar of the sensors subgroup. The analysis of four subgroups showed a high degree of consistency in identifying asleep and awake states, which related to high sensitivity and low specificity, respectively. Compared to the mean values in the subgroups, the results of the EBE agreement for asleep and awake states in terms of accuracy, sensitivity, and specificity are as follows: the piezoelectric sensor (accuracy: N = 2, 0.90 ± 0.01; sensitivity: N = 3, 0.92 ± 0.05; specificity: N = 3, 0.56 ± 0.21) was better than the radiofrequency sensor was (accuracy: N = 19, 0.81 ± 0.06; sensitivity: N = 19, 0.90 ± 0.07; specificity: N = 19, 0.51 ± 0.11); the mattress-based one (accuracy: N = 3, 0.83 ± 0.13; sensitivity: N = 4, 0.91 ± 0.04; specificity: N = 4, 0.52 ± 0.19) was slightly better than the bedside one was (accuracy: N = 19, 0.81 ± 0.06; sensitivity: N = 19, 0.90 ± 0.07; specificity: N = 19, 0.51 ± 0.11); healthy patients (N = 13, 0.94 ± 0.03) had the best sensitivity results; the healthy + patient group (N = 5, 0.53 ± 0.16) was slightly better than the healthy group was (N = 13, 0.52 ± 0.12) in terms of specificity; the healthy (N = 13, 0.84 ± 0.06) and healthy + patient groups (N = 4, 0.82 ± 0.06) generally had consistent accuracy, and the patient group had the worst scores of all; the others group (B) (N = 3, 0.96 ± 0.02) scored the best in terms of sensitivity, and ResMed S had a higher accuracy (N = 4, 0.84 ± 0.09) and specificity (N = 4, 0.59 ± 0.15) than the other brands did. The coefficients of variation (CV) of the piezoelectric sensor, mattress-based devices, the healthy group, and the others group (B) had the lowest sensitivity scores, while the CVs of the radiofrequency sensor, bedside devices, the patient group, and SleepMinder had the lowest specificity results.

#### 3.5.2. Sleep Stage Identification

**Figure 4:**
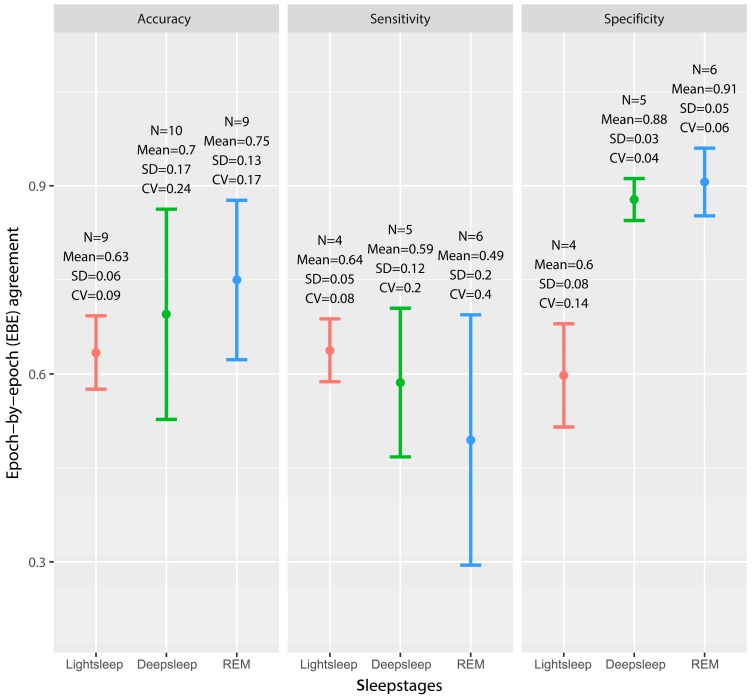
Comparison of the epoch-by-epoch (EBE) agreement for sleep stages in the error bars. N, number of studies; CV, coefficient of variation. Accuracy, proportion of correctly classified epochs for each stage over the total number of epochs; sensitivity, proportion of epochs classified as each stage; specificity, proportion of epochs not classified as each stage [54].

A total of eight studies (n = 390 samples) appraised the performance of CCSTDs using sleep staging functions in identifying sleep stages via EBE analysis [17,39,40,41,42,43,48,49]. Out of these eight studies, two consisted of two or three different samples, thereby increasing the total number of evaluations to eleven. Figure 4 displays the error bar of the EBE agreement between sleep stages. Compared with PSG, CCSTDs had the highest sensitivity (N = 4, 0.64 ± 0.05) and the lowest specificity (N = 4, 0.60 ± 0.08) and accuracy (N = 9, 0.63 ± 0.06) in detecting light sleep. REM showed the opposite result: the highest specificity (N = 6, 0.91 ± 0.05) and accuracy (N = 9, 0.75 ± 0.13), but the lowest sensitivity (N = 6, 0.49 ± 0.20). Deepsleep (CV = 0.06) had the smallest coefficient of variation in specificity, but a high level of specificity (N = 5, 0.88 ± 0.03). Light sleep had the smallest coefficient of variation in terms of sensitivity (CV = 0.08) and accuracy (CV = 0.09).

### 3.6. Comparison of Sleep Parameters Assessed via CCSTDs Versus Actigraphy

Five studies investigated the accuracy of CCSTDs relative to that of actigraphy [34,35,42,49,53]. As reported in Table 1 and Table S2, the participants in one study were patients undergoing an assessment for obstructive sleep apnoea syndrome [35], and participants in the other four studies were healthy people [34,42,49,53]. Two of those studies compared an early or newer version of ResMed S+ and Beddit and employed actigraphy [42,53]. ResMed S + V1 and ResMed S + V2 demonstrated a similar degree of sensitivity (ResMed S + V1 = 0.948; ResMed S + V2 = 0.938; actigraphy = 0.966), a higher degree of specificity (ResMed S + V1 = 0.695; ResMed S + V2 = 0.731; actigraphy = 0.476), and a similar degree of accuracy (ResMed S + V1 = 0.875; ResMed S + V2 = 0.876; actigraphy = 0.851) [42]. Beddit 3.5 showed a higher degree of agreement with PSG in terms of TST (ICC = 0.998), SE (ICC = 0.98), SOL (ICC = 0.92), and WASO (ICC = 0.92) than it did with actigraphy (TST (ICC = 0.96), SE (ICC = 0.81), SOL (ICC = 0.74), and WASO (ICC = 0.44)), while Beddit 3.0 showed a low degree of agreement with PSG, and its TST (ICC = 0.13), SE (ICC = 0.26), SOL (ICC = 0.02), and WASO (ICC = −0.02) were lower than those of actigraphy [53].

Three articles compared five different brands of CCSTDs using actigraphy [34,35,49]. One article compared actigraphy with SleepMinder. The results showed that actigraphy overestimated TST (57 min) and SE (8.8%), and SleepMinder overestimated TST (10 min) and SE (0.8%). Compared to actigraphy, SleepMinder demonstrated a similar degree of accuracy for sleep/wake determination (SleepMinder = 0.773; actigraphy = 0.765), a lower degree of sensitivity (SleepMinder = 0.864; actigraphy = 0.938), and a higher degree of specificity (SleepMinder = 0.518; actigraphy = 0.341) [35]. Another article compared two brands of devices with actigraphy and reported similar results [34]. SleepMinder (0.856), HSL-101 (0.856), and actigraphy (0.855) had similar degrees of accuracy for sleep/wake determination. SleepMinde and HSL-101 showed less sensitivity (SleepMinder = 0.953; HSL-101 = 0.964; actigraphy = 0.973), more specificity (SleepMinder = 0.389; HSL-101 = 0.358; actigraphy = 0.339), as well as slightly improved estimates of TST, SOL, WASO, and SE than actigraphy did. The other article compared three brands of CCSTDs with actigraphy. TST, SE, SOL, and WASO estimations using EarlySense Live, ResMed S+, and SleepScore Max were superior to those of actigraphy. The degree of sensitivity of EarlySense Live (0.96), ResMed S+ (0.93), and SleepScore Max (0.94) was slightly lower than that of actigraphy (0.97). However, the specificities of EarlySense Live (0.47), ResMed S+ (0.51), and SleepScore Max (0.50) were much higher than those of actigraphy (0.39), and the accuracies of EarlySense Live (0.90), ResMed S+ (0.88), and SleepScore Max (0.88) were the same as that of actigraphy (0.89) [49]. In summary, the findings in these three articles are consistent. Compared to actigraphy, CCSTDs have similar degrees of accuracy, less sensitivity, but more specificity.

## 4. Discussion

The aim of the study was to assess the feasibility of CCSTDs as an alternative to PSG and actigraphy using large sample size or via the long-term monitoring of humans through a comprehensive analysis of the effectiveness of CCSTDs. The findings are based on a review of relevant published articles and a meta-analysis.

According to the qualified publications, all CCSTDs can track sleep and wake, among which ResMed S+, EarlySense Live, and Somnofy can also monitor sleep stages. Compared to PSG, CCSTDs overestimated TST, SE and deep sleep, and underestimated SOL and WASO, but showed non-significant difference for light sleep only. The EBE analysis of asleep and awake participants showed that CCSTDs had a high degree of sensitivity (0.90 ± 0.06), but a relatively low degree of specificity (0.51 ± 0.12), indicating a tendency for the devices to accurately detect sleep epochs, but less accurately detect awake epoch. Moreover, there is a wide range of values for accuracy (i.e., between 0.68 and 0.91) of sleep and wake epoch identification. In terms of EBE agreement for sleep stages, the degree of sensitivity was relatively low for light sleep, deep sleep, and REM. The degree of specificity was relatively high, with a narrower range of values for deep sleep, and REM, but low values for light sleep. This indicates an overall poorer and inconsistent ability of CCSTDs to correctly detect sleep stage epochs [17,49]. The results of the analysis of the accuracy for EBE agreement for sleep stages confirm this. In summary, the accuracy of CCSTDs varied widely, which could be attributed to the varied health states of participants and different sensors and algorithms used in CCSTDs.

The health state of participants is one of the most important factors affecting the accuracy of CCSTD sleep monitoring. For healthy participants with normal sleep patterns and behaviors, the CCSTDs results did not significantly differ in assessing sleep stages compared to those of PSG, but for the patient participants, CCSTDs did not show significant difference in terms of REM only. In the cases when healthy participants were involved, CCSTDs demonstrated much higher degrees of accuracy, sensitivity, and specificity than when patients were involved in the EBE analysis of asleep and awake epochs. The same conclusion was confirmed in previous studies that used the same device to study healthy participants and patients [20,31,35,55]. This is because patients who suffer from disturbed sleep are prone to repeated arousal during sleep [56]. The more wakefulness occurs, the more erroneous data there are, leading to sleep overestimation [57]. Hence, sleep/wake identification becomes more inaccurate for patients.

The sensor is the most important factor affecting the accuracy of CCSTDs in sleep monitoring. CCSTDs are used to sense the sleep state by detecting one or more physiological signals, such as chest and abdominal breathing movements, heart movements, and body movements using sensors, such as radiofrequency, infrared light, pressure, and piezoelectricity ones, and microphones [58]. The subgroup analysis of sensors showed that both piezoelectric and radiofrequency sensors performed well in sleep monitoring. However, the piezoelectric sensor had high degree of accuracy in terms of both wakefulness and sleep, while the radiofrequency sensor had high degree of accuracy in terms of sleep only. This is consistent with the results of the EBE analysis, where the piezoelectric sensor had excellent accuracy and sensitivity, and the radiofrequency sensor excelled only in terms of sensitivity. The reason for this is that the piezoelectric sensor collects data on heartbeat rate and respiratory rate, body movements, and sleep postures during sleep, while the radiofrequency sensor obtains sleep data by detecting breathing and body movements [59]. In addition, it is difficult to detect small movements within the body, such as heartbeat or pulse, using current radiofrequency technology, and it cannot monitor multiple people simultaneously [58]. Therefore, the radiofrequency sensor is slightly worse than the piezoelectric sensor in terms of sleep monitoring performance. This could also explain the results of the meta-analysis in which mattress-based devices performed slightly better than bedside devices did in terms of the mean values of sensitivity and specificity for monitoring the sleep period. The other advantages of mattress-based devices over bedside devices are that they not only allow small movements within the body to be more easily detected, but they also allow multiple people in the same room to be monitored simultaneously [16].

It is worth noting that some bedside instruments with better sensor performances and better algorithms show as accuracy values as high as those of mattress instruments, such as ResMed S+, EarlySense, and Somnofy [42,48,49], indicating a significant difference among different brands. The results of the subgroup meta-analysis by brand confirm this. For example, ResMed S+ performed as well PSG did in measuring the sleep parameters in this review.

Therefore, CCSTDs, especially, mattress-based devices with a built-in piezoelectric sensor, demonstrated excellent performances in the sleep monitoring of healthy populations. They can be used as an alternative tool to PSG in monitoring overall sleep conditions in long-period and large-sample healthy population experiments, rather than experiments that require precise data because CCSTDs overestimation or underestimation some sleep parameters to some degree [43,47,49,53].

Actigraphy devices have been extensively validated [34,60], and found to be highly sensitive (0.965) and accurate (0.863), but poorly specific (0.329) to sleep [61]. Actigraphy is used to estimate asleep/awake epochs by measuring body movements using accelerometers. The definition of sleep is a lack of movement. Thus, the brief awake periods that produce a small amount of motion and physical stillness that precede sleep onset are often classified incorrectly as sleep, leading to low specificity, overestimated TST and SE, and underestimated SOL and WASO, especially among individuals with sleep disturbances [61,62,63,64]. The specificity for studies that have included healthy participants ranged from 0.269 to 0.77 [56,65,66,67,68,69,70,71,72,73], whereas others that included a variety of patient groups report specificity values that range from 0.325 to 0.80 [61,74,75,76]. A low degree of specificity is the main limitation of sleep monitoring via actigraphy. Further limitations concern the inability to discriminate between sleep stages. Studies that compared CCSTDs with actigraphy [34,35,42,49,53] revealed that the CCSTDs demonstrated a similar degree of accuracy, a higher degree of specificity, and a lower degree of sensitivity than actigraphy did for sleep/wake epoch identification. TST, SE, SOL, and WASO estimation using CCSTDs were superior to those of actigraphy. The accuracies of actigraphy systems varies widely and are typically dependent on the observed population [77]. The accuracy of actigraphy in determining asleep and awake epochs is reasonably high in the normal subjects [78,79,80]. The American Academy of Sleep Medicine Practice Guidelines indicates that actigraphy represents a reliable method of measuring sleep in the normal healthy adult population [81]. However, the differences in accuracy are large in the presence of sleep disorders, such as sleep-disordered breathing, insomnia, and periodic limb movements [82,83]. For example, in sleep-disordered breathing patients, a per-epoch accuracy of 0.80–0.86 has been reported, and the accuracy tends to be less in severe apnea cases (0.861 in the normal group versus 0.799 in the severe OSA group) [77]. This difference is consistent with the results of EBE agreement for asleep/awake epochs in the subgroup of participant type in this study. In summary, this study shows that CCSTDs can be a valid alternative to the silver standard of actigraphy for sleep monitoring. In addition, CCSTDs have the advantage of detecting sleep stages.

However, the included studies have certain limitations. First, the participants were mostly monitored for one night. Second, there is a lack of published information, such as that about the algorithm and the reliability of hardware, to explain the different performances of various CCSTDs. Third, most of the included studies analyzed only one or two sleep indicators using the device, which may have led to a bias in the final results. In addition, a wide range of CCSTDs have appeared on the market. They may have the problems of unstandardized, undisclosed, and unvalidated data and algorithms [54,84]. Hence, in the face of the constant introduction of new devices and algorithms, it is essential to refer to the gold standard, PSG, and perform validation evaluations for specific populations before using them in human experiments.

## 5. Conclusions

This systematic review and meta-analysis have shown that CCSTDs, especially, mattress-based devices with a piezoelectric sensor, have a better accuracy when they are used in experiments involving healthy participants. They can be used as an alternative tool to PSG for long-period and large-sample human experiments that do not require precise data. Furthermore, CCSTDs demonstrate accuracy values as high as those for actigraphy and can provide data on sleep stages that are not available with the use of actigraphy. Therefore, CCSTDs can be an effective alternative tool to actigraphy for monitoring sleep.

## Figures and Tables

**Table 1:** A detailed summary of qualified publications.

The First Author (Year)	Country	Device	Sensors	Reference and Comparison	Participants			Investigative Details		
					Total Number (Female Number)	Age (Years), Mean (SD), and/or Range	Type	(Duration) Study Site	Tracker Placement	Bed-Time
Zhang (2010) [30]	China	MMSM (RS-611)	Pressure	PSG	40 (5)	29–71	OSAHS ^1^	(2 nights) Sleep lab	Under-mattress	Habitual
De Chazal (2011) [31]	Ireland	SleepMinder	Radiofrequency	PSG	113 (19)	53 (13)	Healthy, sleep apnoea	(1 night) Sleep lab	Bedside table	Habitual ^2^
					50 (3)	50 (14)	AHI ≤ 15			
					63 (16)	57 (12)	AHI > 15			
Hashizaki (2014) [32]	Japan	SleepMinder	Radiofrequency	PSG	148 (35)	43.7 (12.1) ≥18	AHI ≤ 15 & AHI > 15	(1 night) Sleep lab	Bedside table	Habitual ^2^
					49 (14)	37.7 (11.6) ≥18	AHI ≤ 15			
					99 (21)	46.7 (11.2) ≥18	AHI > 15			
Norman (2014) [33]	Australia	Sonomat	Piezoelectric	PSG	62 (25)	26 (16) ≥18	OSA and Healthy	(1 night) Sleep lab/home	Under-mattress	Habitual ^2^
O’Hare (2014) [34]	Ireland	SleepMinder	Radiofrequency	PSG	20 (9)	30 (6) ≥18	Healthy	(1 night) Sleep lab	Bedside table	22:30–6:30
		SleepDesign (HSL-101)	Radiofrequency							
Pallin (2014) [35]	Ireland	SleepMinder	Radiofrequency	PSG & actigraphy	103 (19)	55 (14)	OSA ^3^, non-OSA	(1 night) Sleep lab	Bedside table	Habitual ^2^
Abad (2016) [36]	Spain	SleepWise	Infrared camera	PSG	50	53.1 (14.0) >18	Healthy, Mild OSA, Moderate OSA, Severe OSA	(1 night) Sleep lab	Bedside (60 cm above thorax)	Habitual ^2^
Terjung (2016) [37]	Germany	SleepMinder	Radiofrequency	PSG	57 (11)	57.1 (13.4); 32–84	Healthy, OSA and PLM ^4^	(1 night) Sleep lab	Bedside table	Habitual ^2^
Norman (2017) [38]	Australia	Sonomat	Piezoelectric	PSG	76 (30)	5.8 (2.8) 2–17	Suspected SDB ^5^	(1 night) Sleep lab	Under-mattress	Habitual ^2^
Tal (2017) [39]	Israel	EarlySense	Piezoelectric	PSG	43 (9)	45.9 (14.4) 17–72	Suspected SDB	(1 night) Sleep lab	Under-mattress	Habitual ^2^
					7 (4)	31.2 (10.8) 24–65	Healthy	(2–3 nights) Home		
					13 (5)	40.0 (10) 29–59	Healthy	(2–3 nights) Home		
Zaffaroni (2017) [40]	Ireland	ResMed S+	Radiofrequency	PSG	18 (10)	32.6 (10.9) >18	AHI ≤ 15	(1 night) Sleep lab	Bedside table	Habitual ^2^
Chung (2018) [41]	Korea	ResMed S+	Radiofrequency	PSG	8 (1)	45.0 (14.6)	Suspected OSA	(1 night) Sleep lab	Bedside table	23:00–5:00
Schade (2019) [42]	USA	ResMed S + V1	Radiofrequency	PSG & actigraphy	27 (11) (V1 = 27, V2 = 22)	29.1 (11.7) ≥18	Healthy	(1 night) Sleep lab	Bedside table	Habitual–6:00
		ResMed S + V2	Radiofrequency							
Tuominen (2019) [43]	Finland	Beddit Sleep Tracker	Piezoelectric	PSG	10 (5)	24.5 (2.51) 18–30	Healthy	(2 nights) Sleep lab	Under-mattress	Habitual ^2^
Zaffaroni (2019) [44]	Ireland	ResMed S+	Radiofrequency	PSG	62(31)	46.9(15.9) ≥18	AHI < 5, PLM < 30/h	(1 night) Sleep lab	Bedside table	Habitual ^2^
Feng (2020) [45]	China	IR-UWB	Radiofrequency	PSG	40 (11)	38.3 (9.67) 16~60	OSA, non-OSA	(1 night) Sleep lab	Bedside table	——
Miyata (2020) [46]	Japan	SD102 HSAT sleep recorder	Pressure	PSG	189 (56)	56.1 (18.3) ≥20	OSA, PLMs, Central sleep apnea	(1 night) Sleep lab	Under-mattress	Habitual ^2^
Nagatomo (2020) [20]	Japan	Nemuri SCAN	Pressure	PSG	11 (3)	71 (2.82) ≥20	Perioperative, septic shock	(1 day) ICU	Under-mattress	21:00–05:99 ^6^
Stone (2020) [47]	USA	Beddit Sleep Monitor 3.0	Piezoelectric	PSG	5 (3)	27.8 (7.7) 22–41	Healthy	(average 19.6 nights) Home	Under-mattress	Habitual ^2^
Toften (2020) [48]	Norway	Somnofy	Radiofrequency	PSG	71 (43)	28.9 (9.7) 19–61	Healthy	(1 night) Clinic bedrooms and home	Nightstand and wall	Habitual ^2^
Chinoy (2021) [49]	USA	EarlySense Live	Piezoelectric	PSG & actigraphy	34 (22)	28.1 (3.9) 18–35	Healthy	(3 nights) Sleep lab	Under-mattress	Habitual
		ResMed S+	Radiofrequency						Bedside table	
		SleepScore Max	Radiofrequency							
Edouard (2021) [50]	France	Withings Sleep Analyzers (WSA)	Pressure	PSG	118 (67)	49.3 (12.1) 18–70	Healthy, OSA, comorbidities, epilepsy	(1 night) Sleep lab	Under the mattress	——
Ellender (2021) [51]	Australia	Beddit	Piezoelectric	PSG	54 (31) (42 Beddit, 29 ResMed S+)	48.09 (18.05) >18	OSA, insomnia, central hypersomnolence disorder	(1 night) Sleep lab	Under-mattress	——
		ResMed S+	Radiofrequency						Bedside	——
Xue (2021) [52]	China	UWB Radar Sleep Monitoring System	Radiofrequency	PSG	198 (78)	45.5 (4.05) ≥18	Healthy, OSA	(1 night) Sleep lab	Bedside table	22:00–6:00
Hsiou (2022) [53]	USA	Beddit 3.0	Piezoelectric	PSG & actigraphy	35 (27)	18.97 (0.95) ≥18	Healthy	(1 night) Sleep lab	Under the bedsheet	22:30–7:00 h; 1:30–7:00 h
		Beddit 3.5	Piezoelectric							
Kholghi (2022) [17]	Australia	EMFIT Quantified Sleep (QS)	Pressure	PSG	33 (15)	53.7 (16.5) 18–80	Healthy; OSA, PLM	(1 night) Sleep lab	Under the mattress or mattress topper	Habitual ^2^

^1^ OSAHS: obstructive sleep apnea–hypopnea syndrome. ^2^ Exact bedtime is not reported, but investigative protocol infers habitual bedtime. ^3^ OSA: obstructive sleep apnea. ^4^ PLMS: periodic limb movement in sleep. ^5^ SDB: sleep-disordered breathing. ^6^ Inclusion of nighttime sleep data only.

**Table 2:** Meta-analyses results.

Outcome	N	n	Pooled Mean	95% CI	I^2^ (*p*)	Z (*p*)
TST (min)	35	1873	19.55	12.22, 26.88	88.2% (0.000)	5.23 (0.000)
SOL (min)	17	796	−4.61	−6.56, −2.66	51.1% (0.008)	−4.63 (0.000)
WASO (min)	16	846	−12.07	−18.75, −5.38	56.1% (0.003)	−3.54 (0.000)
SE (%)	28	1391	2.88	1.58, 4.17	34.2% (0.041)	4.36 (0.000)
Light Sleep (min)	10	417	5.62	−12.81, 24.06	86.8% (0.000)	0.60 (0.550)
Deep Sleep (min)	10	417	11.07	0.37, 21.76	75.3% (0.000)	2.03 (0.043)
REM (min)	10	417	3.44	−14.81, 21.68	95.3% (0.000)	0.37 (0.712)

N: number of studies; n: number of samples; CI, confidence interval; I^2^: heterogeneity; Z: test of overall effect; TST, total sleep time; SOL, sleep onset latency; WASO, wake after sleep onset; SE, sleep efficiency; REM, rapid eye movement.

## Data Availability

Not applicable.

## Supplementary Materials

The following supporting information can be downloaded at: https://www.mdpi.com/article/10.3390/s23104842/s1.
