# Supplementary material for: Evaluation of the Accuracy of Contactless Consumer Sleep-Tracking Devices Application in Human Experiment: A Systematic Review and Meta-Analysis
Source: Sensors (Basel). 2023 May 17;23(10):4842. doi: 10.3390/s23104842 (PMC10222058; doi:10.3390/s23104842)
Supplement: Supplementary file 1 [file sensors-23-04842-s001.zip › sensors-2214885-supplementary.pdf]

Evaluation of the Accuracy of Contactless Consumer Sleep-Tracking Devices Application in Human Experimentation: A Systematic Review and Meta-Analysis

Supplemental Tables

Table S1. Search strategy for the meta-analyses.
Table S2. Results from qualified publications.
Tables S3-S6. Meta-subgroup analysis results of subgroups.

Supplemental Figures

Figure S1 Tabular presentation of QUADAS-2 results.
Figure S2 Graphical presentation of QUADAS-2 results.
Figure S3 Funnel plot with pseudo 95% confidence limits.
Figures S4-S31 Forest plot meta-analyses for subgroups.

Supplemental Tables

Table S1. Search strategy for the meta-analyses.

Database	Search Terms	Total Studies
PubMed		298
	(sleep*[Title/Abstract]) AND ((noncontact*[Title/Abstract]) OR (non-contact*[Title/Abstract]) OR (nonwearable[Title/Abstract]) OR (non-wearable[Title/Abstract]) OR (nontouch*[Title/Abstract]) OR (non-touch*[Title/Abstract]) OR (contact-free[Title/Abstract]) OR (contactless[Title/Abstract]) OR (*mattress*[Title/Abstract]) OR (sheet*[Title/Abstract]) OR (Emfit[Title/Abstract]) OR (Beddit[Title/Abstract]) OR (EarlySense[Title/Abstract]) OR (RestOn[Title/Abstract]) OR (Dot[Title/Abstract]) OR (Withings[Title/Abstract]) OR (SleepScore[Title/Abstract]) OR (ResMed[Title/Abstract]) OR (Somnofy[Title/Abstract]) OR (Nemuri SCAN[Title/Abstract]) OR (Isleep[Title/Abstract]) OR (Sonomat[Title/Abstract]) OR (SleepWise[Title/Abstract]) OR (SleepMinder[Title/Abstract])) AND (("Polysomnography"[Mesh]) OR (PSG[Title/Abstract]))	
EMBASE		864
	('sleep'/exp OR 'sleep' OR 'sleeping') AND ('non contact' OR noncontact OR nonwearable OR 'non wearable' OR nontouch OR 'non touch' OR contact-free OR 'mattress'/exp<br>OR sheet% OR contactless OR emfit OR beddit OR earlysense OR reston OR dot OR w ithings OR sleepscore OR resmed OR somnofy OR 'nemuri<br>scan' OR isleep OR sonomat OR sleepwise OR sleepminder) AND ('polysomnography'/exp OR psg)	
Cochrane Library		13
	#1 MeSH descriptor: [Sleep] explode all trees 6477<br>#8 (noncontact*):ti,ab,kw (Word variations have been searched) 696<br>#9 (non-contact*):ti,ab,kw (Word variations have been searched) 506<br>#10 (nonwear*):ti,ab,kw (Word variations have been searched) 53<br>#11 (non-wear*):ti,ab,kw (Word variations have been searched) 47<br>#12 (nontouch*):ti,ab,kw (Word variations have been searched) 27<br>#13 (non-touch*):ti,ab,kw (Word variations have been searched) 28<br>#14 (contact-free):ti,ab,kw (Word variations have been searched) 13<br>#15 (contactless):ti,ab,kw (Word variations have been searched) 35<br>#16 (*mattress*):ti,ab,kw (Word variations have been searched) 974<br>#17 (sheet*):ti,ab,kw (Word variations have been searched) 5235<br>#18 (Emfit):ti,ab,kw (Word variations have been searched) 2<br>#19 (Beddit):ti,ab,kw (Word variations have been searched) 4	

	#20 (EarlySense):ti,ab,kw (Word variations have been searched) 4<br>#21 (RestOn):ti,ab,kw (Word variations have been searched) 39<br>#22 (Dot):ti,ab,kw (Word variations have been searched) 1446<br>#23 (Withings):ti,ab,kw (Word variations have been searched) 23<br>#25 (SleepScore Max):ti,ab,kw (Word variations have been searched) 4<br>#26 (Somnofy):ti,ab,kw (Word variations have been searched) 1<br>#27 (Nemuri SCAN):ti,ab,kw (Word variations have been searched) 1<br>#28 (Isleep):ti,ab,kw (Word variations have been searched) 15<br>#29 (Sonomat):ti,ab,kw (Word variations have been searched) 1<br>#30 (SleepWise):ti,ab,kw (Word variations have been searched) 0<br>#31 (SleepMinder):ti,ab,kw (Word variations have been searched) 1<br>#32 (ResMed):ti,ab,kw (Word variations have been searched) 163<br>#33 #2 OR #3 OR #4 OR #5 OR #6 OR #7 OR #8 OR #9 OR #10 OR #11 OR #12 OR #13 OR #14 OR #15 OR #16 OR #17 #18 OR #19 OR #20 OR #21 OR #22 OR #23 OR #24 OR #25 8691<br>#35 MeSH descriptor: [Polysomnography] explode all trees 1959<br>#36 (PSG):ti,ab,kw (Word variations have been searched) 1530<br>#37 #27 OR #28 3224<br>#38 #1 AND #33 AND #34 AND #37 13	
Web of Science		568
	1: TS=(sleep*) Results: 585334<br>2: TS=(noncontact*) OR TS=(non-contact*) OR TS=(nonwear*) OR TS=(non-wear*) OR TS=(nontouch*) OR TS=(non-touch*) OR TS=(contact-free) OR TS=(contactless) OR TS=(*mattress*) OR TS=(sheet*) OR TS=(Emfit) OR TS=(Beddit) OR TS=(EarlySense) OR TS=(RestOn) OR TS=(Dot) OR TS=(Withings) OR TS=(SleepScore) OR TS=(ResMed) OR TS=(Somnofy) OR TS=(Nemuri SCAN) OR TS=(Isleep) OR TS=(Sonomat) OR TS=(SleepWise) OR TS=(SleepMinder) Results: 3562802<br>3: TS=(polysomnography) OR TS=(somnography) OR TS=(PSG) Results: 45568<br>4: #1 AND #2 AND #3 Results: 568	
Total after de-duplication		*1744*

Note. Tiab, title abstract; exp, "explodes" the category to include all terms under the umbrella term; ab,ti, abstract and title' ti,ab,kw, title abstract keyword; tw, title word

Table S2. Results from qualified publications

First author (year)	Device	Results[1]
Zhang (2010) [1]	MMSM(RS-611)	• Overestimated SE[2] (20.9±4.1%), light sleep[2] (18.5±2.2%), REM (0.8±1.0%, NS[3]); underestimated deep sleep[2] (19.4±1.7%)[4].

De Chazal (2011) [2]	SleepMinder	• All subjects: Overestimated TST (19.0±58.6 min), SE (4.8±14.4%); accuracy (78.0±11.1%), sensitivity (87.3±13.6%), specificity (50.1±19.8%)[5]; PPV[6] (81.4±13.9%), NPV[7] (66.1±24.3%);<br>• Low AHI: Overestimated TST (27.6±57.7 min), SE (7.0±14.1%); accuracy (81.3±10.0%), sensitivity (92.0±9.2%), specificity (51.1±19.1%)[5]; PPV (83.3±13.6%), NPV (72.4±21.4%);<br>• High AHI: Overestimated TST (8.2±58.6 min), SE (1.9±14.4%); accuracy (74.8±10.5%), sensitivity (81.1±15.8%), specificity (48.8±20.8%)[5]; PPV (78.9±14.2%), NPV (57.9±25.5%).
Hashizaki (2014) [3]	SleepMinder	• All subjects: Overestimated TST (12.7±63.9 min), SE (3.0±13.3 %); underestimated SOL (5.0±9.9 min), WASO (9.6±59.0 min); accuracy (84.1±11.1%), sensitivity (91.8±12.8%), specificity (37.6±21.8%)[5];<br>• AHI≤15: Overestimated TST (12.4±31.8 min), SE (3.1±6.6%); underestimated SOL (3.7±7.5 min), WASO (10.4±31.1 min); accuracy (90.3±5.1%), sensitivity (96.0±4.9%), specificity (38.9±22.6%)[5];<br>• AHI>15: Overestimated TST (12.8±75.0 min), SE (2.9±15.7%); underestimated SOL (5.6±10.9 min), WASO (9.1±68.9 min); accuracy (81.0±12.0%), sensitivity (89.7±14.9%), specificity (36.9±21.4%)[5].
O'Hare (2014) [4]	SleepMinder	• Overestimated TST[8] (35.0±13.3 min), SE[8] (6.4±3.0 %); underestimated SOL[8] (10.0±3.7 min), WASO[8] (23.0±12.7 min); accuracy (85.6 ± 6.2%), sensitivity (95.3 ± 3.8%), specificity (38.9 ± 19%)[5].
	SleepDesign (HSL-101)	• Overestimated TST[8] (43.0±12.0 min), SE[8] (8.1±2.7 min); underestimated SOL[8] (15.0±3.1 min), WASO[8] (26.0±11.4 min); accuracy (85.6±6.4%), sensitivity (96.4±2.4%), specificity (35.8±14.2%)[5].
	Actigraphy	• Overestimated TST[8] (50.0±11.5 min), SE[8] (9.6±2.6 min); underestimated SOL[8] (16.0±2.9 min), WASO[8] (31.0±11.0 min); accuracy (85.5±6.3%), sensitivity (97.3±2.0%), specificity (33.9±12.7%)[5].
Pallin (2014) [5]	SleepMinder	• All subjects: Overestimated TST (10±67 min, NS), SE (0.8±15.8%, NS); accuracy 77.3%, sensitivity 86.4%, specificity 51.8%[5];<br>• AHI<5: Overestimated TST[2] (34±43 min), SE[2] (5.5±7.7%); accuracy 82.1%, sensitivity 93.9%, specificity 49.0%[5];<br>• AHI 5-15: Overestimated TST[8] (27±68 min), SE[8] (3.4±13.5%); accuracy 81.2%, sensitivity 92.0%, specificity 46.6%[5];<br>• AHI 15-30: Overestimated TST (-22±76 min, NS), SE (-6.9±21.1%, NS); accuracy 71.5%, sensitivity 76.3%, specificity 61.3%[5];<br>• AHI >30: Overestimated TST (-4.5±71 min, NS), SE (0.1±16.5%, NS); accuracy 73.6%, sensitivity 82.1%, specificity 53.3%[5].
	Actigraphy	• Overestimated TST[2] (57±61 min), SE[2] (8.8±13.4%); accuracy 76.5%, sensitivity 93.8%, specificity 34.1[5]; PPV (83.3±13.6%); NPV (72.4±21.4%);<br>• AHI<5: Overestimated TST[2] (70±62 min), SE[2] (11.4±11.2%); accuracy 76.6%, sensitivity 95.5%, specificity 28.4%[5]; PPV (83.3±13.6%), NPV (72.4±21.4%);<br>• AHI 5-15: Overestimated TST[2] (57±49 min), SE[2] (7.2±11.5%); accuracy 79.2%, sensitivity 96.2%, specificity 32.9%[5];<br>• AHI 15-30: Overestimated TST[2] (63±68 min), SE[2] (11.6±15.7%); accuracy 74.2%, sensitivity 93.0%, specificity 29.9%[5];<br>• AHI >30: Overestimated TST[2] (44±68 min), SE[8] (6.9±15.1%); accuracy 74.8%, sensitivity 90.4%, specificity 42.6%[5].
Norman (2014) [6]	Sonomat	• Overestimated TST[2] (36.5±1.9 min).

Abad (2016) [7]	SleepWise	• Underestimated TST (7.2±8.8 min, NS).
Terjung (2016) [8]	SleepMinder	• Overestimated TST (34.0±10.5 min), SE (8.1±2.0%).
Norman (2017) [9]	Sonomat	• Overestimated TST (2.6±3.9 min, NS).
Tal (2017)[10]	EarlySense	• Sleep laboratory (setup I): accuracy 88.5%, sensitivity 89.7%, specificity 83.4%[5]; sensitivity in detecting light sleep 63.3%, deep sleep 53.6%, REM 40.0%;<br>• Home (setup II): accuracy 92.1%, sensitivity 95.4%, specificity 72.1%[5];<br>• Home (setup III): accuracy 92.5%, sensitivity 95.1%, specificity 79.0%[5].
Zaffaroni (2017) [11]	ResMed S+	• Overestimated SOL (5.2±5.8 min, NS); underestimated TST (6.3±11.4 min, NS), SE (0.2±1.2 min, NS), SOL (0.7±3.5min, NS), light sleep (2.5±12.9 min, NS), deep sleep (3.0±7.5min,NS), REM (0.9±7.0 min, NS); accuracy in detecting wake 92%, deep sleep 88%,REM 89%; sensitivity in detecting sleep 62%, deep sleep 68%, REM 73%; specificity in detecting wake 95%, deep sleep 93%, REM 94%.
Chung (2018) [12]	ResMed S+	• Sensitivity in sleep/wake 75.3%, REM 14.6%; specificity in sleep/wake 43.1%, REM 89.6%.
Zaffaroni（2019） [13]	ResMed S+	• Overestimated TST (8.5±9.7 min, NS), light sleep (0.4±6.5 min, NS), REM[2] (13.7±3.8 min); underestimated SOL (0.7±3.5min, NS), WASO (7.8±6.1min, NS), deep sleep (5.8±5.6min, NS).
Schade (2019) [14]	ResMed S+V1	• Overestimated TST (17.7±61.4 min), light sleep (6.4±52.2 min), deep sleep (1.0±50.0 min), REM (10.2±26.6 min); accuracy 87.5%, sensitivity 94.8%, specificity 69.5%[5]; accuracy in detecting light sleep 64.0%, deep sleep 61.1%, REM 61.5%.
	ResMed S+V2	• Overestimated TST (13.2±51.4 min), light sleep (12.8±41.2 min), REM (10.7±28.5min); underestimated deep sleep (-10.5±40.3 min); accuracy 87.6%, sensitivity 93.8%, specificity 73.1%[5]; accuracy in detecting light sleep 65.1%, deep sleep 52.2%, REM 61.6%.
	Actigraphy	• Overestimated TST (43.4±53.5 min); accuracy 85.1%, sensitivity 96.6%, specificity 47.6%[5].
Tuominen (2019) [15]	Beddit Sleep Tracker	• Overestimated TST[2] (43.5± 17.9 min), SE[2] (7.3±2.4%); underestimated SOL (3.2±6.6 min, NS), WASO[2] (32.6±8.8 min); specificity 42.1%; accuracy in detecting light sleep 55.6%, deep sleep 37.5%.
Miyata（2020） [16]	SD 102	• Total subjects: correlation TST[8] (r=-0.151), SE[2] (r=-0.283);<br>• Men: correlation TST[8] (r=-0.214), SE[2] (r=-0.377);<br>• Women: correlation TST (r=-0.042, NS), SE (r=-0.105, NS).
Stone（2020） [17]	Beddit Sleep Monitor 3.0	• Overestimated TST[2] (30.0±34.0 min), SE[2] (7.2±7.1%).
Nagatomo (2020) [18]	Nemuri SCAN	• Daytime: overestimated TST (337.2±38.0 min), SE (39.5±3.8%);<br>• Nighttime: overestimated TST (46.9±16.1 min), SE (10.9±3.1%); accuracy 68.4%; sensitivity 90.1%, specificity 38.8%5; PPV 66.8%, NPV 74.2%.
Toften (2020) [19]	Somnofy	• Overestimated TST (5.1±20.8 min), SOL (0.01±21.3 min), SE (1.0±4.3%), deep sleep(4.4±34.6min), REM (3.4±28.0 min); underestimated WASO (5.1±22.0 min), light sleep (2.7±35.7 min); accuracy 0.76, sensitivity 0.97, specificity 0.72; accuracy in detecting light sleep 0.75, deep sleep 0.74, REM 0.78.
Feng (2020) [20]	IR-UWB	• Overestimated SE (0.4±1.8%), deep sleep (40.3±12.7min), REM (5.5±7.4 min); underestimated light sleep (85.3±20.5min).
Chinoy (2021) [21]	EarlySense Live	• Overestimated TST[2] (13.6±29.9 min), SE[2] (2.9±6.2%), SOL (0.8±8.2 min, NS), deep sleep[2] (35.9±52.4 min), REM (0.0±42.3 min, NS); underestimated WASO[2] (-15.3±19.5 min), light sleep[2] (-22.3±56.8 min); accuracy 0.90, sensitivity 0.96,

		specificity 0.47[5]; accuracy in detecting light sleep 0.63, deep sleep 0.81, REM 0.84; sensitivity in detecting light sleep 0.57, deep sleep 0.68, REM 0.64; specificity in detecting light sleep 0.69, deep sleep 0.84, REM 0.89.
	ResMed S+	• Underestimated TST (-0.3±35.9 min, NS), WASO (-3.4±32.9 min, NS), REM (-31.9±27.4 min); overestimated SE (0.0±7.5%, NS), SOL[8] (4.0±14.8 min), light sleep[2] (17.1±38.5 min), deep sleep[2] (14.5±35.4 min); accuracy 0.88, sensitivity 0.93, specificity 0.51[5]; accuracy in detecting light sleep 0.64, deep sleep 0.83, REM 0.85; sensitivity in detecting light sleep 0.67, deep sleep 0.59, REM 0.50; specificity in detecting light sleep 0.61, deep sleep 0.88, REM 0.95; PPV 0.93, NPV 0.62.
	SleepScore Max	• Overestimated TST (7.5±34.8 min, NS), SE (1.6±7.2%, NS), SOL[8] (4.4±12.0 min), light sleep[2] (22.7±37.9 min), deep sleep[2] (20.7±29.1 min);underestimated WASO[8] (-12.1±34.9 min), REM[2] (-35.8±30.4 min); accuracy 0.88, sensitivity 0.94, specificity 0.50[5]; accuracy in detecting light sleep 0.64, deep sleep 0.84, REM 0.84; sensitivity in detecting light sleep 0.68, deep sleep 0.59, REM 0.49; specificity in detecting light sleep 0.60, deep sleep 0.88, REM 0.95.
	Actigraphy	• Overestimated TST[2] (23.9±33.4 min), SE[2] (5.0±6.9%); underestimated SOL[2] (-7.6±8.4 min), WASO[2] (-16.6±32.9 min); accuracy 0.89, sensitivity 0.97, specificity 0.39[5].
Edouard (2021) [22]	Withings Sleep Analyzers (WSA)	• Overestimated TST[8] (25.7±8.4min, NS), SE (0.1±1.5%, NS); underestimated WASO[2] (17.5±5.6 min).
Ellender (2021) [23]	Beddit	• Overestimated SOL (44.6±59.4min, NS); underestimated TST[2] (53.4±92.7 min), SE (1.4±18.7 min, NS).
	ResMed S+	• Overestimated SOL (35.6±46.6 min, NS), WASO2 (27.2±50.4 min); underestimated TST8 (34.4±113.6min), SE (15.9±19.1min, NS).
Xue (2021) [24]	UWB Radar Sleep Monitoring System	• Overestimated TST[2] (16.7±1.1min).
Hsiou (2022) [25]	Beddit 3.0	• Overestimated TST (45.4±108.0 min), WASO (25.7±59.0 min); underestimated SE (-1.0±14.7%), SOL (-13.4±23.2 min).
	Beddit 3.5	• Overestimated TST (1.57±7.4 min), SOL (6.4±6.1 min), WASO (21.7±14.9 min); underestimated SE (-1.1±2.1%).
	Actigraphy	• Underestimated TST (-2.2±28.3 min), SE (-1.0±5.7%), SOL (-4.4±15.6 min), WASO (-3.2±29.3 min).
Kholghi (2022) [26]	EMFIT Quantified Sleep (QS)	• Overestimated TST[2] (177.5±119.4 min), light sleep (94.2±90.4min), deep sleep (8.3±39min, NS), REM (75.0±40.87min); underestimated WASO[2] (44.7 ± 68.8min), SOL (5.6± 46.4 min, NS).

[1]Accuracy, sensitivity, and specificity in detecting sleep epochs are reported unless otherwise specified.

[2] P<.01.

[3] NS: not significant.

[4] Differences for PSG and MMSM(RS-611) in light sleep, deep sleep, and REM Sleep staging composition ratio.

[5] Epoch-by-epoch sleep–wake agreement between polysomnography and the CCSTD.

[6] PPV, positive predictive value (percentage of epochs labeled as sleep that are correctly labeled).

[7] NPV, negative predictive value (percentage of epochs labeled as wake that are correctly labeled).

[8] *P*<.05.

Meta-subgroup analysis results of subgroups.

Table S3. Meta-subgroup analyses of sensors.

Outcome	Subgroup	N	Pooled mean	95% CI	I^2 (P)	Z (P)
**TST (min)**	Piezoelectric	8	20.89	-0.74, 42.53	96.6%(0.000)	1.89(0.058)
	Pressure	3	80.29	6.83, 153.76	94.5% (0.000)	2.14 (0.032)
	Radiofrequency	23	16.27	14.26, 18.28	0.0% (0.460)	15.87(0.000)
	Infrared camera	1	-7.20	-24.50, 10.10	——	-0.82(0.415)
**SOL (min)**	Piezoelectric	5	-2.48	-9.19, 4.22	50.8% (0.087）	-0.73(0.468)
	Pressure	1	-5.60	-21.09, 9.89	——	-0.71 (0.478)
	Radiofrequency	11	-5.21	-7.16, -3.26	47.4%（0.040）	-5.24 (0.000)
**WASO (min)**	Piezoelectric	4	-2.29	-26.95, 22.37	85.1% (0.000)	-0.18 (0.855)
	Pressure	2	-29.09	-55.54, -2.65	76.5% (0.039)	-2.16 (0.031)
	Radiofrequency	10	-9.80	-15.07, -4.53	0.0%（0.846）	-3.64 (0.000)
**SE (%)**	Piezoelectric	6	3.19	0.59, 5.79	14.9% (0.318)	2.40 (0.016)
	Pressure	2	5.16	-5.38, 15.71	90.0% (0.002)	0.96 (0.337)
	Radiofrequency	20	2.61	1.23, 4.00	22.6% (0.176)	3.69 (0.000)
**Light Sleep (min)**	Piezoelectric	1	-22.30	-38.80, -5.80	——	-2.65 (0.008)
	Pressure	1	94.20	61.59, 126.81	——	5.66 (0.000)

	Radiofrequency	8	1.57	-13.81, 16.95	75.4% (0.000)	0.20 (0.842)
**Deep Sleep (min)**	Piezoelectric	1	35.90	21.49, 50.31	——	4.88 (0.000)
	Pressure	1	8.30	-9.39, 25.99	——	0.92(0.358)
	Radiofrequency	8	7.66	-3.48, 18.80	75.3% (0.000)	1.35(0.178)
**REM (min)**	Piezoelectric	1	0.00	-13.31, 13.31	——	0.00 (1.000)
	Pressure	1	62.00	47.80, 76.20	——	8.56 (0.000)
	Radiofrequency	8	-3.97	-21.14, 13.21	93.4% (0.000)	-0.45 (0.651)

N, number of samples; CI, confidence interval; I^2, Heterogeneity; Z, Test of overall effect; TST, total sleep time; SOL, sleep onset latency; WASO, wake after sleep onset; SE, sleep efficiency; REM, rapid eye movement.

Table S4. Meta-subgroup analyses of device types.

Outcome	Subgroup	N	Pooled mean	95% CI	I^2(P)	Z(P)
**TST (min)**	Mattress-based	11	36.96	18.16, 55.75	96.1% (0.000)	3.85 (0.000)
	Bedside	24	19.55	12.22, 26.88	20.6% (0.181)	4.91 (0.000)
**SOL (min)**	Mattress-based	6	-2.76	-8.47, 2.94	40.5% (0.136)	-0.949 (0.343)
	Bedside	11	-4.61	-6.56, -2.66	47.4% (0.040)	-5.236 (0.000)
**WASO (min)**	Mattress-based	6	-12.34	-28.76, 4.07	81.6% (0.000)	-1.474 (0.141)
	Bedside	10	-9.80	-15.07, -4.53	0.0% (0.846)	-3.64 (0.000)
**SE (%)**	Mattress-based	8	3.44	0.40, 6.48	56.7% (0.023)	2.218 (0.027)
	Bedside	20	2.62	1.23, 4.00	22.6% (0.176)	3.69 (0.000)
**Light Sleep (min)**	Mattress-based	2	35.07	-79.09, 149.22	97.4% (0.000)	0.602 (0.547)
	Bedside	8	1.57	-13.81, 16.95	75.4% (0.000)	2.00 (0.842)
**Deep Sleep**	Mattress-based	2	22.60	-4.44, 49.63	82.2% (0.018)	1.638

(min)						(0.101)
	Bedside	8	7.66	-3.48, 18.80	70.2% (0.001)	1.35 (0.178)
REM(min)	Mattress-based	2	30.95	-29.81, 91.71	97.4% (0.000)	0.998 (0.318)
	Bedside	8	-3.97	-21.14，13.21	93.4% (0.000)	-0.45 (0.651)

N, number of samples; CI, confidence interval; I^2, Heterogeneity; Z, Test of overall effect; TST, total sleep time; SOL, sleep onset latency; WASO, wake after sleep onset; SE, sleep efficiency; REM, rapid eye movement.

Table S5. Meta-subgroup analyses of participant types.

Outcome	Subgroup	N	Pooled mean	95% CI	I^2 (P)	Z (P)
TST (min)	Healthy	18	13.44	6.57, 20.32	13.1% (0.297)	3.83 (0.005)
	Patients	7	19.91	4.23, 35.58	0.0% (0.590)	2.49 (0.013)
	Healthy+ Patients	10	19.55	12.67, 36.80	96.6% (0.000)	4.02 (0.000)
SOL (min)	Healthy	12	-4.40	-7.39, -1.41	62.3% (0.002）	-2.88 (0.004)
	Patients	3	-5.66	-7.93, -3.38	0.0% (0.472)	-4.88 (0.000)
	Healthy+Patients	2	-5.01	-6.75, -3.27	0.0%（0.940）	-5.64 (0.000)
WASO (min)	Healthy	11	-9.76	-18.24, -1.28	59.2% (0.006)	-2.26 (0.024)
	Patients	3	-14.57	-23.67, -5.47	0.0% (0.501)	-3.14 (0.002)
	Healthy+Patients	2	-25.68	-60.15, 8.79	85.3%（0.009）	-1.46 (0.144)
SE (%)	Healthy	14	2.53	0.95, 4.10	30.6% (0.125)	3.15 (0.002)
	Patients	10	3.65	0.39, 6.90	55.1% (0.018)	2.19 (0.028)
	Healthy+Patients	3	3.00	0.19, 5.81	0.0% (0.983)	2.09 (0.036)
Light Sleep (min)	Healthy	8	4.96	-6.86, 16.78	65.6% (0.005)	0.834 (0.404)
	Patients	1	-85.32	-125.57,	——	-4.16 (0.000)

				-45.07		
	Healthy+Patients	1	94.20	61.59, 126.81	——	5.662 (0.000)
**Deep Sleep (min)**	Healthy	8	8.43	-3.52, 20.39	77.3% (0.000)	1.38 (0.167)
	Patients	1	40.34	15.49, 65.19	——	3.182 (0.001)
	Healthy+Patients	1	8.30	-9.39, 25.99	——	0.920 (0.358)
**REM (min)**	Healthy	8	-4.69	-21.58, 12.20	93.3% (0.000)	-0.54 (0.586)
	Patients	1	5.45	-8.97, 19.87	——	0.741 (0.459)
	Healthy+Patients	1	62.00	47.80, 76.20	——	8.556 (0.000)

N: number of samples; CI, confidence interval; I^2: Heterogeneity; Z: Test of overall effect; TST, total sleep time; SOL, sleep onset latency; WASO, wake after sleep onset; SE, sleep efficiency; REM, rapid eye movement.

Table S6. Meta-subgroup analyses of brands.

Outcome	Subgroup	N	Pooled mean	95% CI	I^2 (P)	Z (P)
**TST (min)**	SleepMinder	13	19.24	10.63, 27.84	0.0% (0.945)	4.38 (0.000)
	Sonomat	2	19.53	-13.69, 52.75	99.5% (0.000)	1.15 (0.249)
	ResMed S+	5	3.51	-5.74, 12.76	0.0% (0.659)	0.74 (0.457)
	Beddit	6	32.86	4.56,61.15	0.0% (0.833)	2.28 (0.023)
	Others (Mattress-based)	4	59.51	17.00,102.02	93.5% (0.000)	2.74 (0.006)
	Others(Bedside)	5	13.06	-0.50,26.61	69.9% (0.010)	1.89 (0.059)
**SOL (min)**	SleepMinder	4	-5.08	-6.33, -3.82	3.8% (0.374)	-7.94 (0.000)

	ResMed S+	3	-1.76	-5.96, 2.45	12.8% (0.318)	-0.82 (0.413)
	Beddit	5	-2.69	-13.89, 8.51	49.8% (0.093)	-0.47 (0.638)
	Other (Mattress-based)	2	-0.99	-4.05, 2.08	0.0% (0.551)	-0.63 (0.527)
	Other(Bedside)	3	-9.05	-18.47, 0.37	72.9% (0.025)	-1.88 (0.060)
**WASO (min)**	SleepMinder	4	-11.01	-18.67, -3.34	0.0% (0.799)	-2.82 (0.005)
	ResMed S+	2	-5.73	-14.42, 2.97	0.0% (0.621)	-1.29 (0.197)
	Beddit	4	6.26	-31.62, 44.13	84.7% (0.000)	0.32 (0.746)
	Others (Mattress-based)	3	-22.09	-35.12, -9.05	60.8% (0.078)	-3.32 (0.001)
	Others(Bedside)	3	-16.39	-29.72, -3.06	0.0% (0.552)	-2.41 (0.016)
**SE (%)**	SleepMinder	13	4.50	2.95, 6.04	0.0% (0.772)	5.70 (0.000)
	ResMed S+	2	-0.12	-2.00, 1.76	0.0% (0.919)	-0.13 (0.900)
	Beddit	6	2.78	-1.86, 7.42	26.9% (0.233)	1.18 (0.240)
	Others (Mattress-based)	3	3.90	-0.71, 8.52	80.3% (0.006)	1.66 (0.097)

	Others(Bedside)	4	0.96	-1.53, 3.45	0.0% (0.977)	0.75 (0.452)
**Light Sleep (min)**	ResMed S+	5	7.92	0.00, 15.84	3.7% (0.385)	1.96 (0.050)
	Other (Mattress-based)	2	35.07	-79.09, 149.22	97.4% (0.000)	0.60 (0.547)
	Other(Bedside)	3	-21.55	-98.41, 55.32	91.8% (0.000)	-0.55 (0.583)
**Deep Sleep (min)**	ResMed S+	5	0.43	-9.43, 10.29	50.9% (0.086)	0.09 (0.932)
	Others (Mattress-based)	2	22.60	-4.44, 49.63	82.2% (0.018)	1.64 (0.101)
	Others(Bedside)	3	24.66	11.64, 37.68	13.8% (0.314)	3.71 (0.000)
**REM (min)**	ResMed S+	5	0.10	-20.12, 20.32	93.8% (0.000)	0.10 (0.992)
	Others (Mattress-based)	2	30.95	-29.81, 91.71	97.4% (0.000)	1.00 (0.318)
	Others(Bedside)	3	-11.72	-46.45, 23.00	91.1% (0.000)	-0.66 (0.508)

N, number of samples; CI, confidence interval; I^2, Heterogeneity; Z, Test of overall effect; TST, total sleep time; SOL, sleep onset latency; WASO, wake after sleep onset; SE, sleep efficiency; REM, rapid eye movement.

Supplemental Figures

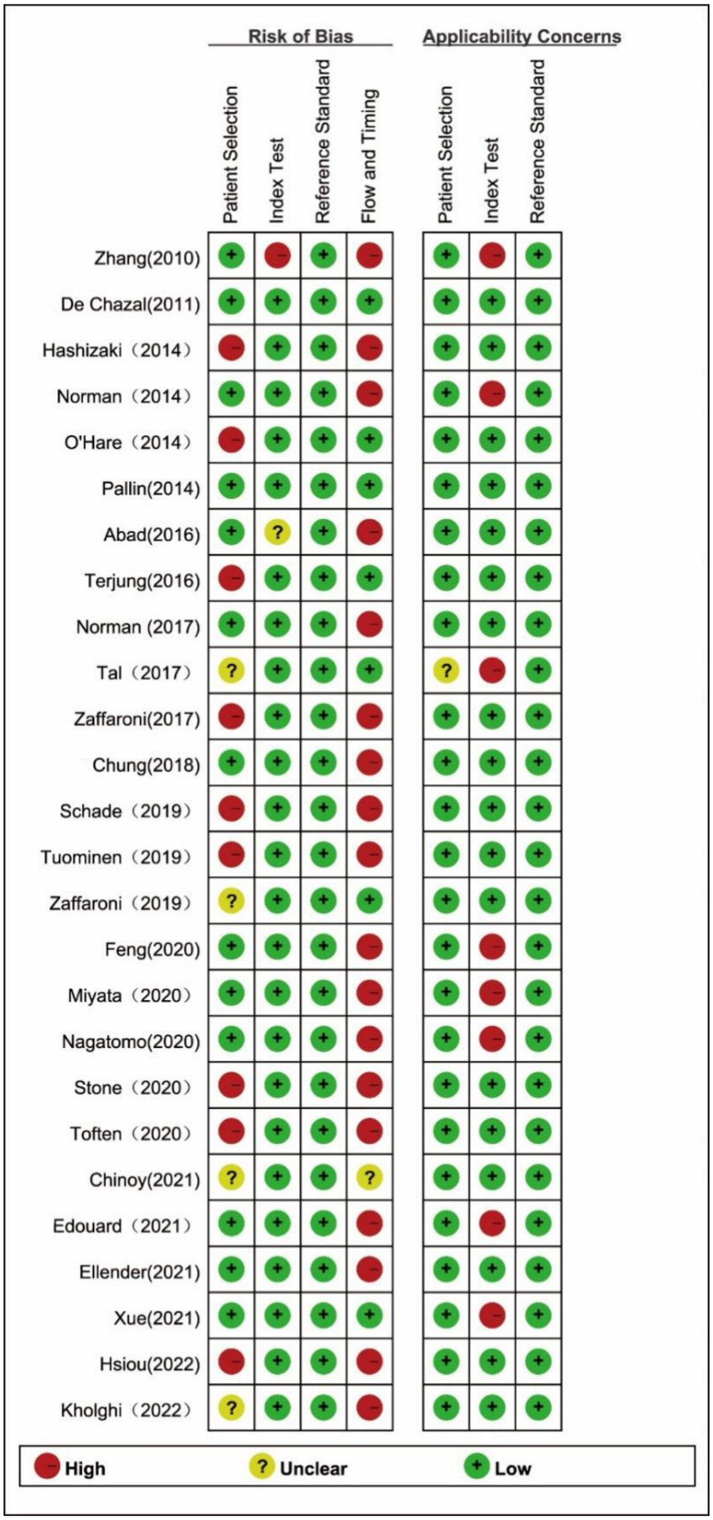

Figure S1. Tabular presentation of QUADAS-2 results.

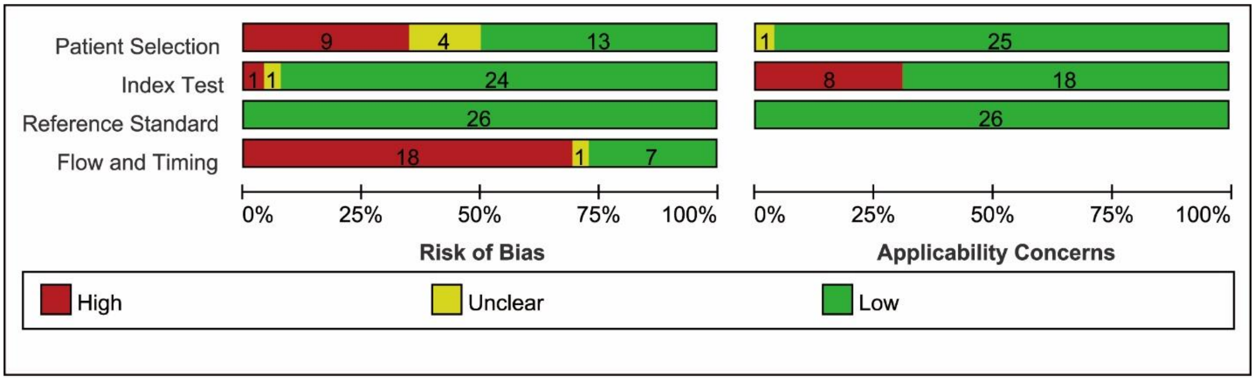

Figure S2. Graphical presentation of QUADAS-2 results.

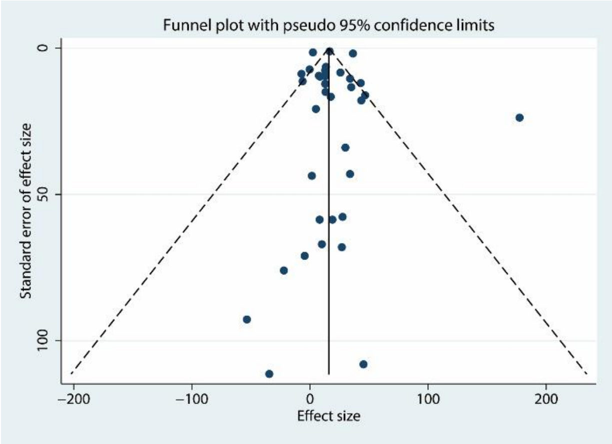

a.TST

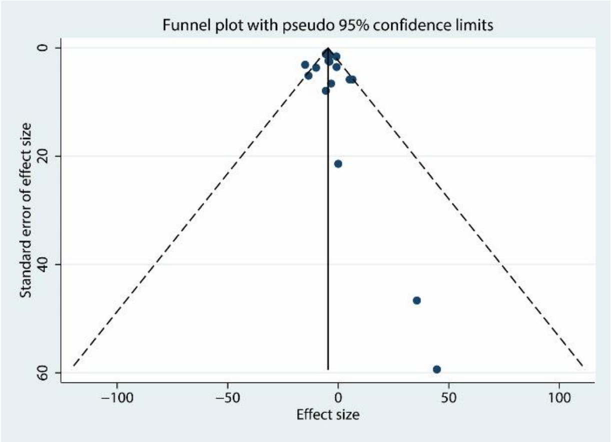

b.SOL

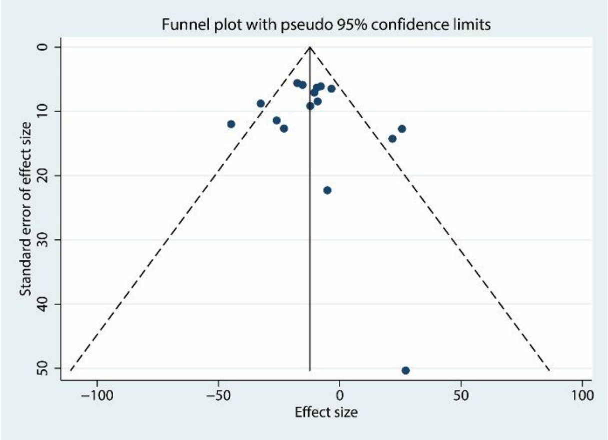

c.WASO

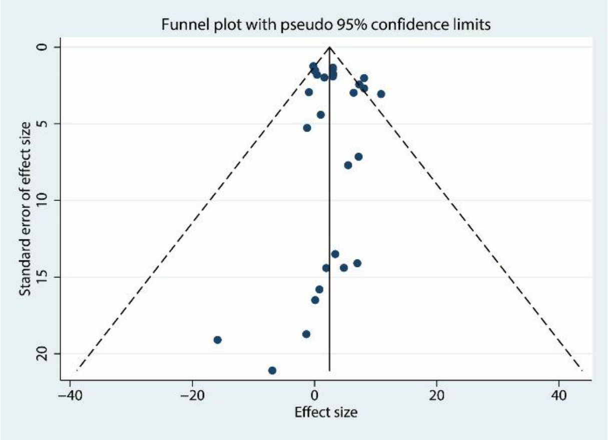

d.SE

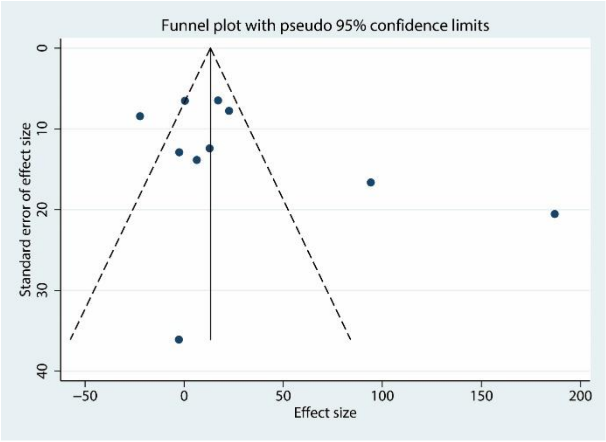

e. Light Sleep

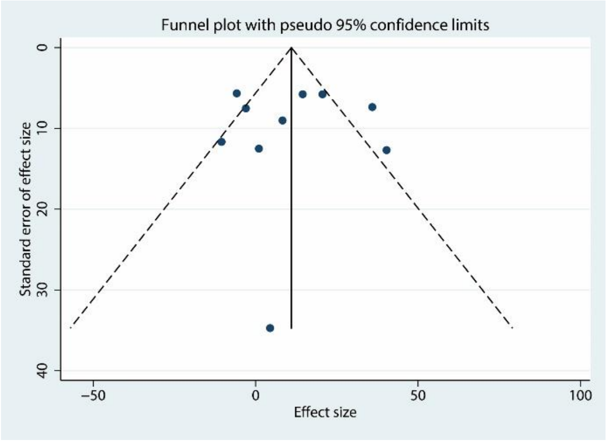

f. Deep Sleep

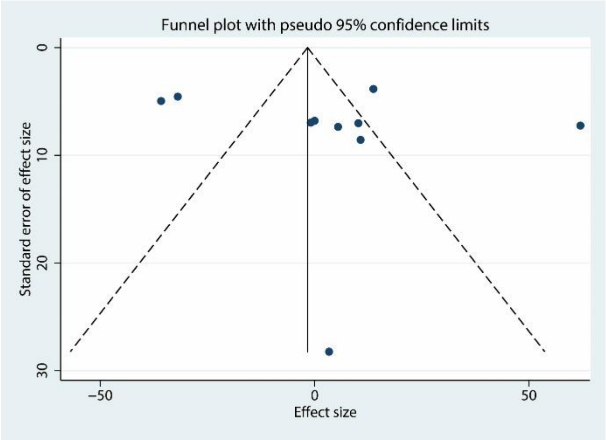

g. REM

TST, total sleep time; SOL, sleep onset latency; WASO, wake after sleep onset; SE, sleep efficiency, REM, rapid eye movement.

Figure S3. Funnel plot with pseudo 95% confidence limits.

Forest plot meta-analyses for subgroups.

This section lists the subgroups analysis results by expanding the search heterogeneity discussed in 3.4.1. Subgroup meta-analyses of TST, WASO, SOL, SE, light sleep, deep sleep, and REM were performed for the following variables: (1) the type of sensors, including the piezoelectric sensor, pressure sensor, radiofrequency sensor, and infrared camera; (2) the type of devices, including the mattress-based and bedside devices; (3) the variety of participants (ie, healthy participants, patients participants, and healthy and patients participants); (4) the brand of devices (the number of samples of the same brand greater than or equal to two will be included in the same group, and the other samples are divided into 1)other brands of bedside device, and 2)other brands of mattress device). The Forest plot meta-analyses are provided in Figures S4 to S31. A range of subgroups analysis results of sensors, device types, participant types, and brands are presented in Tables S3-S6.

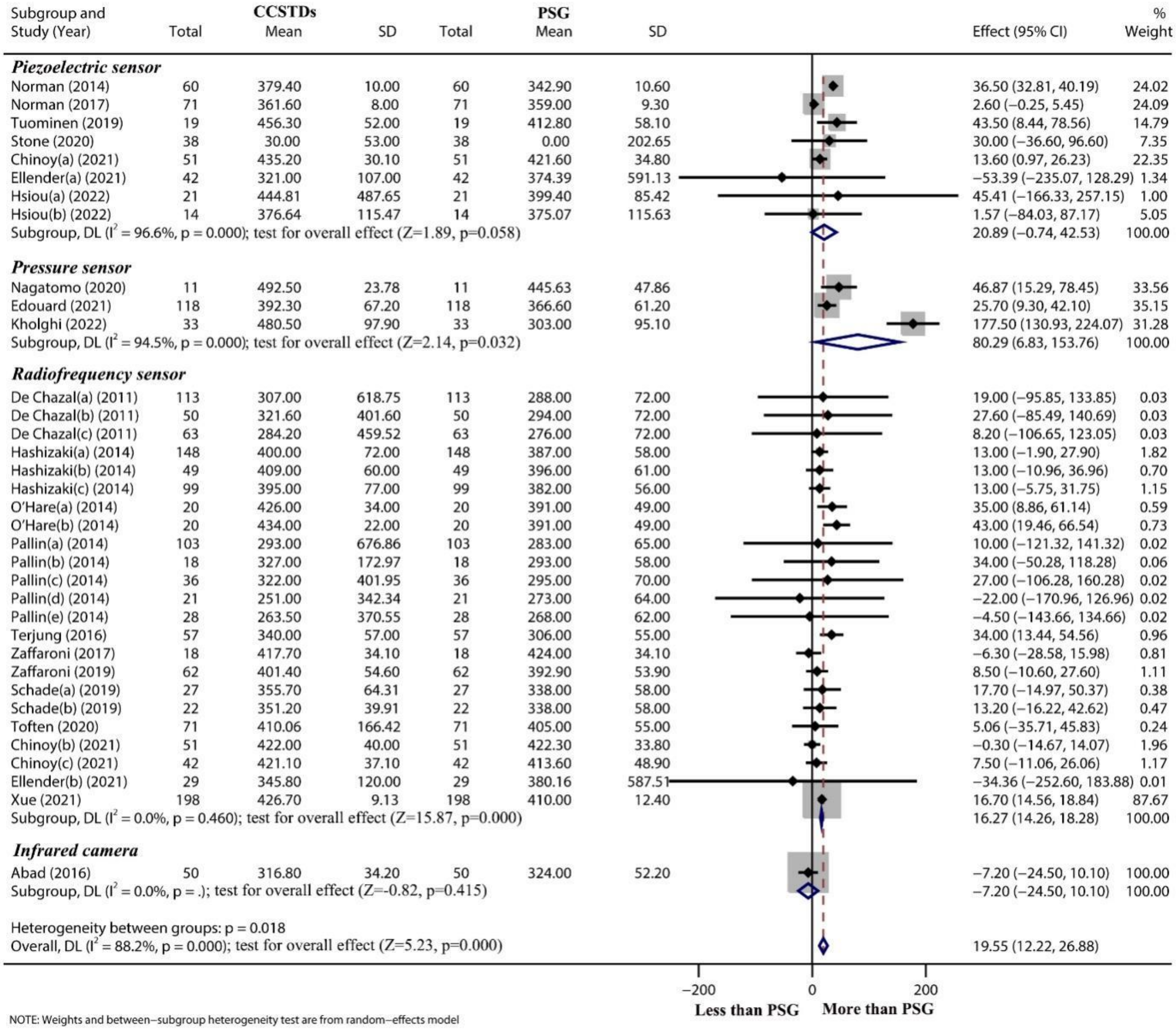

Figure S4. Forest plot meta-analyses for sensors subgroup -TST (min).

Subgroup and Study (Year)	CCSTDs Total	CCSTDs Mean	CCSTDs SD	PSG Total	PSG Mean	PSG SD	Effect (95% CI)	% Weight
Mattress-based								
Norman (2014)	60	379.40	10.00	60	342.90	10.60	36.50 (32.81, 40.19)	15.67
Norman (2017)	71	361.60	8.00	71	359.00	9.30	2.60 (−0.25, 5.45)	15.71
Tuominen (2019)	19	456.30	52.00	19	412.80	58.10	43.50 (8.44, 78.56)	10.18
Stone (2020)	38	30.00	53.00	38	0.00	202.65	30.00 (−36.60, 96.60)	5.29
Nagatomo (2020)	11	492.50	23.78	11	445.63	47.86	46.87 (15.29, 78.45)	10.91
Chinoy(a) (2021)	51	435.20	30.10	51	421.60	34.80	13.60 (0.97, 26.23)	14.72
Edouard (2021)	118	392.30	67.20	118	366.60	61.20	25.70 (9.30, 42.10)	14.07
Ellender(a) (2021)	42	321.00	107.00	42	374.39	591.13	−53.39 (−235.07, 128.29)	1.00
Hsiou(a) (2022)	21	444.81	487.65	21	399.40	85.42	45.41 (−166.33, 257.15)	0.75
Hsiou(b) (2022)	14	376.64	115.47	14	375.07	115.63	1.57 (−84.03, 87.17)	3.69
Kholghi (2022)	33	480.50	97.90	33	303.00	95.10	177.50 (130.93, 224.07)	8.01
Subgroup, DL (I^2 = 96.1%, p = 0.000); test for overall effect (Z=3.85, p=0.000)							36.96 (18.16, 55.75)	100.00
Bedside								
De Chazal(a) (2011)	113	307.00	618.75	113	288.00	72.00	19.00 (−95.85, 133.85)	0.21
De Chazal(b) (2011)	50	321.60	401.60	50	294.00	72.00	27.60 (−85.49, 140.69)	0.21
De Chazal(c) (2011)	63	284.20	459.52	63	276.00	72.00	8.20 (−106.65, 123.05)	0.20
Hashizaki(a) (2014)	148	400.00	72.00	148	387.00	58.00	13.00 (−1.90, 27.90)	8.59
Hashizaki(b) (2014)	49	409.00	60.00	49	396.00	61.00	13.00 (−10.96, 36.96)	4.07
Hashizaki(c) (2014)	99	395.00	77.00	99	382.00	56.00	13.00 (−5.75, 31.75)	6.09
O'Hare(a) (2014)	20	426.00	34.00	20	391.00	49.00	35.00 (8.86, 61.14)	3.50
O'Hare(b) (2014)	20	434.00	22.00	20	391.00	49.00	43.00 (19.46, 66.54)	4.19
Pallin(a) (2014)	103	293.00	676.86	103	283.00	65.00	10.00 (−121.32, 141.32)	0.16
Pallin(b) (2014)	18	327.00	172.97	18	293.00	58.00	34.00 (−50.28, 118.28)	0.38
Pallin(c) (2014)	36	322.00	401.95	36	295.00	70.00	27.00 (−106.28, 160.28)	0.15
Pallin(d) (2014)	21	251.00	342.34	21	273.00	64.00	−22.00 (−170.96, 126.96)	0.12
Pallin(e) (2014)	28	263.50	370.55	28	268.00	62.00	−4.50 (−143.66, 134.66)	0.14
Abad (2016)	50	316.80	34.20	50	324.00	52.20	−7.20 (−24.50, 10.10)	6.91
Terjung (2016)	57	340.00	57.00	57	306.00	55.00	34.00 (13.44, 54.56)	5.26
Zaffaroni (2017)	18	417.70	34.10	18	424.00	34.10	−6.30 (−28.58, 15.98)	4.60
Zaffaroni (2019)	62	401.40	54.60	62	392.90	53.90	8.50 (−10.60, 27.60)	5.92
Schade(a) (2019)	27	355.70	64.31	27	338.00	58.00	17.70 (−14.97, 50.37)	2.34
Schade(b) (2019)	22	351.20	39.91	22	338.00	58.00	13.20 (−16.22, 42.62)	2.84
Toften (2020)	71	410.06	166.42	71	405.00	55.00	5.06 (−35.71, 45.83)	1.55
Chinoy(b) (2021)	51	422.00	40.00	51	422.30	33.80	−0.30 (−14.67, 14.07)	9.03
Chinoy(c) (2021)	42	421.10	37.10	42	413.60	48.90	7.50 (−11.06, 26.06)	6.19
Ellender(b) (2021)	29	345.80	120.00	29	380.16	587.51	−34.36 (−252.60, 183.88)	0.06
Xue (2021)	198	426.70	9.13	198	410.00	12.40	16.70 (14.56, 18.84)	27.29
Subgroup, DL (I^2 = 20.6%, p = 0.181); test for overall effect (Z=4.91, p=0.023)							13.08 (7.86, 18.30)	100.00
Heterogeneity between groups: p = 0.016								
Overall, DL (I^2 = 88.2%, p = 0.000); test for overall effect (Z=5.23, p=0.000)							19.55 (12.22, 26.88)	

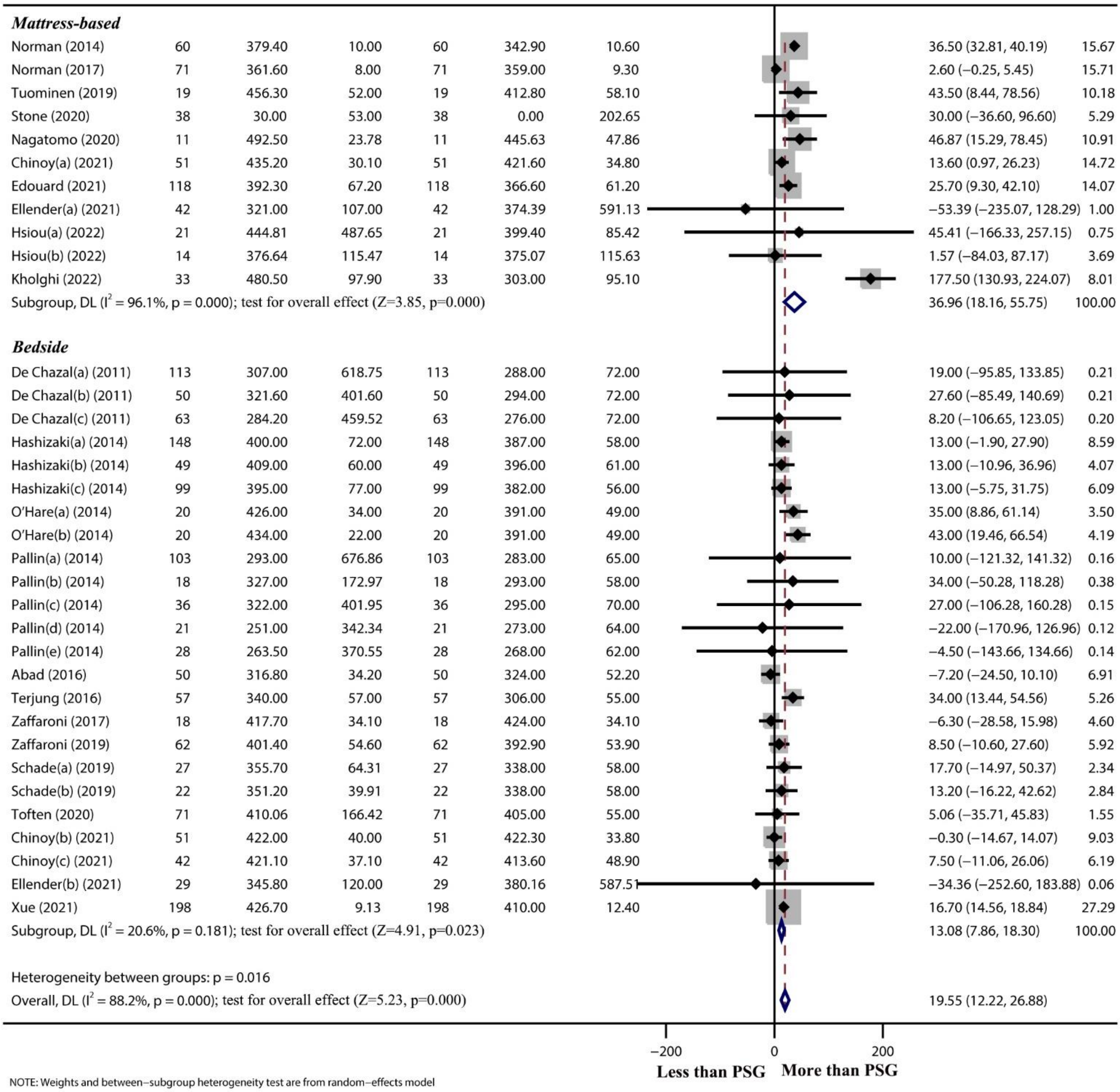

NOTE: Weights and between−subgroup heterogeneity test are from random−effects model

Figure S5. Forest plot meta-analyses for device types subgroup -TST (min).

Subgroup and Study (Year)	CCSTDs Total	CCSTDs Mean	CCSTDs SD	PSG Total	PSG Mean	PSG SD	Effect (95% CI)	% Weight
Healthy								
De Chazal(b) (2011)	50	321.60	401.60	50	294.00	72.00	27.60 (−85.49, 140.69)	0.40
Hashizaki(b) (2014)	49	409.00	60.00	49	396.00	61.00	13.00 (−10.96, 36.96)	3.88
O'Hare(a) (2014)	20	426.00	34.00	20	391.00	49.00	35.00 (8.86, 61.14)	3.60
O'Hare(b) (2014)	20	434.00	22.00	20	391.00	49.00	43.00 (19.46, 66.54)	3.94
Pallin(b) (2014)	18	327.00	172.97	18	293.00	58.00	34.00 (−50.28, 118.28)	0.68
Pallin(c) (2014)	36	322.00	401.95	36	295.00	70.00	27.00 (−106.28, 160.28)	0.29
Zaffaroni (2017)	18	417.70	34.10	18	424.00	34.10	−6.30 (−28.58, 15.98)	4.12
Zaffaroni (2019)	62	401.40	54.60	62	392.90	53.90	8.50 (−10.60, 27.60)	4.58
Schade(a) (2019)	27	355.70	64.31	27	338.00	58.00	17.70 (−14.97, 50.37)	2.86
Schade(b) (2019)	22	351.20	39.91	22	338.00	58.00	13.20 (−16.22, 42.62)	3.21
Tuominen (2019)	19	456.30	52.00	19	412.80	58.10	43.50 (8.44, 78.56)	2.64
Stone (2020)	38	30.00	53.00	38	0.00	202.65	30.00 (−36.60, 96.60)	1.02
Toften (2020)	71	410.06	166.42	71	405.00	55.00	5.06 (−35.71, 45.83)	2.17
Chinoy(a) (2021)	51	435.20	30.10	51	421.60	34.80	13.60 (0.97, 26.23)	5.55
Chinoy(b) (2021)	51	422.00	40.00	51	422.30	33.80	−0.30 (−14.67, 14.07)	5.29
Chinoy(c) (2021)	42	421.10	37.10	42	413.60	48.90	7.50 (−11.06, 26.06)	4.66
Hsiou(a) (2022)	21	444.81	487.65	21	399.40	85.42	45.41 (−166.33, 257.15)	0.12
Hsiou(b) (2022)	14	376.64	115.47	14	375.07	115.63	1.57 (−84.03, 87.17)	0.66
Subgroup, DL (I^2 = 13.1%, p = 0.297); test for overall effect (Z=3.83, p=0.000)							13.44 (6.57, 20.32)	49.65
Patient								
De Chazal(c) (2011)	63	284.20	459.52	63	276.00	72.00	8.20 (−106.65, 123.05)	0.38
Hashizaki(c) (2014)	99	395.00	77.00	99	382.00	56.00	13.00 (−5.75, 31.75)	4.63
Pallin(d) (2014)	21	251.00	342.34	21	273.00	64.00	−22.00 (−170.96, 126.96)	0.23
Pallin(e) (2014)	28	263.50	370.55	28	268.00	62.00	−4.50 (−143.66, 134.66)	0.27
Nagatomo (2020)	11	492.50	23.78	11	445.63	47.86	46.87 (15.29, 78.45)	2.97
Ellender(a) (2021)	42	321.00	107.00	42	374.39	591.13	−53.39 (−235.07, 128.29)	0.16
Ellender(b) (2021)	29	345.80	120.00	29	380.16	587.51	−34.36 (−252.60, 183.88)	0.11
Subgroup, DL (I^2 = 0.0%, p = 0.590); test for overall effect (Z=2.49, p=0.013)							19.91 (4.23, 35.58)	8.75
Healthy+Patient								
De Chazal(a) (2011)	113	307.00	618.75	113	288.00	72.00	19.00 (−95.85, 133.85)	0.38
Hashizaki(a) (2014)	148	400.00	72.00	148	387.00	58.00	13.00 (−1.90, 27.90)	5.21
Pallin(a) (2014)	103	293.00	676.86	103	283.00	65.00	10.00 (−121.32, 141.32)	0.30
Norman (2014)	60	379.40	10.00	60	342.90	10.60	36.50 (32.81, 40.19)	6.53
Abad (2016)	50	316.80	34.20	50	324.00	52.20	−7.20 (−24.50, 10.10)	4.85
Terjung (2016)	57	340.00	57.00	57	306.00	55.00	34.00 (13.44, 54.56)	4.36
Norman (2017)	71	361.60	8.00	71	359.00	9.30	2.60 (−0.25, 5.45)	6.57
Edouard (2021)	118	392.30	67.20	118	366.60	61.20	25.70 (9.30, 42.10)	4.98
Xue (2021)	198	426.70	9.13	198	410.00	12.40	16.70 (14.56, 18.84)	6.60
Kholghi (2022)	33	480.50	97.90	33	303.00	95.10	177.50 (130.93, 224.07)	1.80
Subgroup, DL (I^2 = 96.6%, p = 0.000); test for overall effect (Z=4.02, p=0.000)							24.73 (12.67, 36.80)	41.59
Heterogeneity between groups: p = 0.256								
Overall, DL (I^2 = 88.2%, p = 0.000); test for overall effect (Z=5.23, p=0.000)							19.55 (12.22, 26.88)	100.00

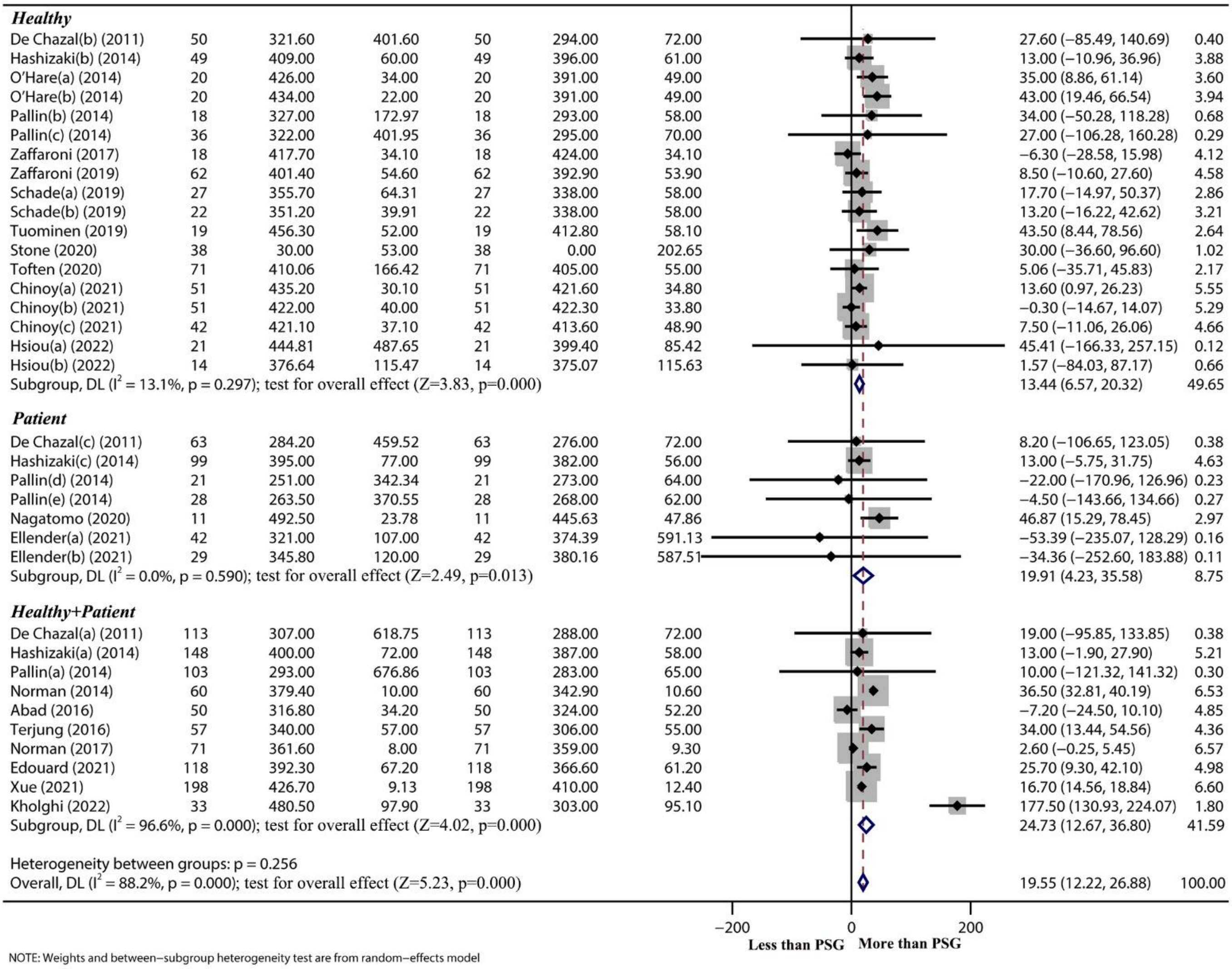

NOTE: Weights and between−subgroup heterogeneity test are from random−effects model

Figure S6. Forest plot meta-analyses for participant types subgroup - TST (min).

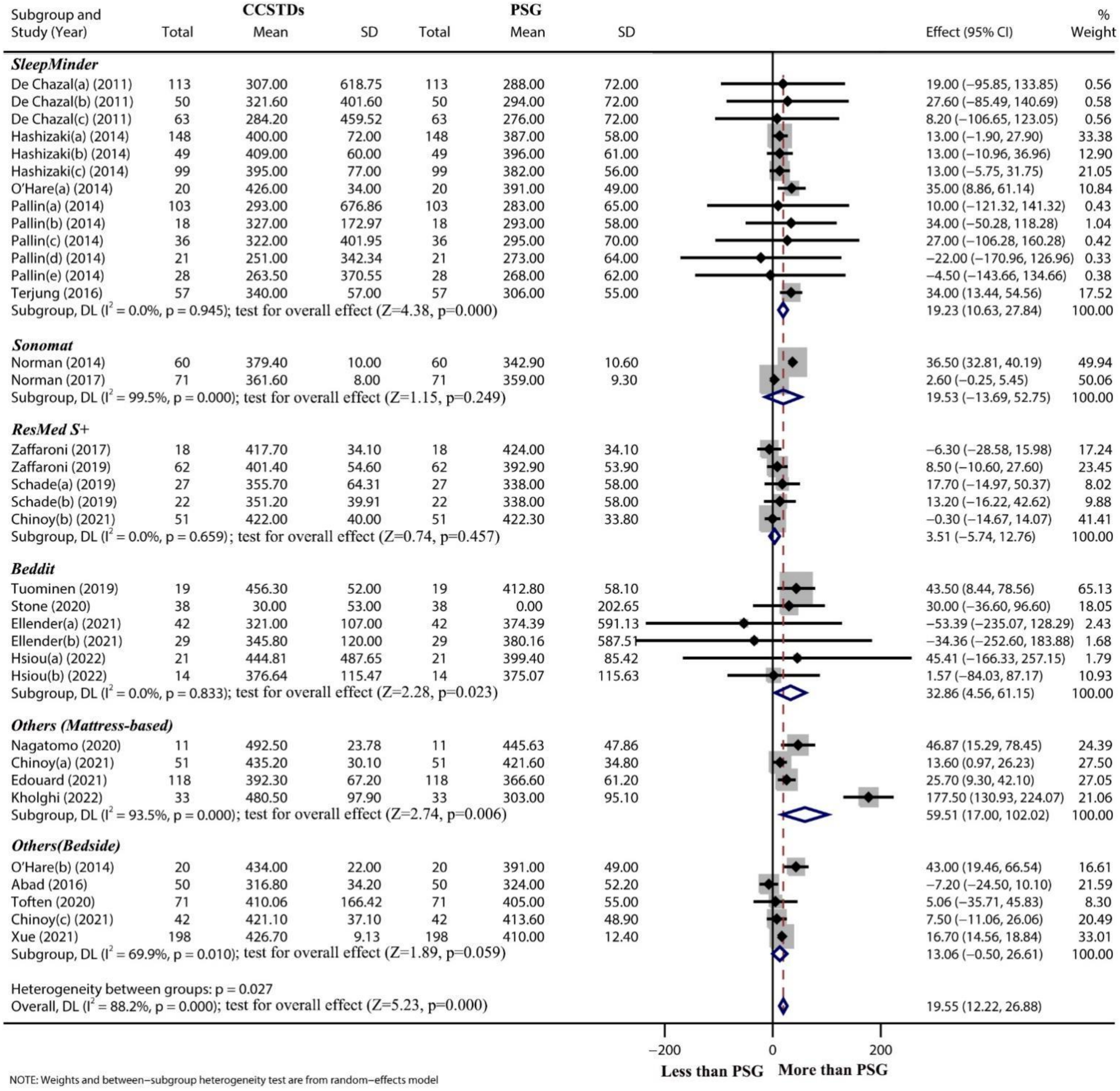

Figure S7. Forest plot meta-analyses for brands subgroup - TST (min).

- TST

As shown in Figures S4 to S7, compared CCSTDs with PSG, the subgroup analyses of sensors and brands revealed a nonsignificant difference of the piezoelectric sensor (N=8 studies; pooled mean=20.89 min, 95% CI -0.74 to 42.53; P=.058), infrared camera (N=1 study; pooled mean=-7.20 min, 95% CI -24.50 to 10.10; P=.415), Sonomat (N=2 studies; pooled mean=19.53 min, 95% CI -13.69 to 52.75; P=.249), ResMed S+ (N=5 studies; pooled mean=3.52 min, 95% CI -5.74 to 12.76; P=.457), and other brands of

bedside device (hereinafter referred to as “Others (Bedside)”) (N=5 studies; pooled mean=13.06 min, 95% CI -0.50 to 26.61; P=.059). However, bedside devices (hereinafter referred to as “Bedside”) (N=24 studies; pooled mean=19.55 min, 95% CI 12.22 to 26.88; P<.001), and healthy participants (hereinafter referred to as “Healthy”) (N=18 studies; pooled mean=13.44 min, 95% CI 6.57 to 20.32; P<.01) showed smaller mean differences in the respective subgroups. There was no significant heterogeneity in radiofrequency sensor (I^2=0.0%; *p*=.460), Bedside (I^2=20.6%; *p*=.181), patients (I^2=0.0%; *p*=.590), Healthy (I^2=13.1%; *p*=.297), SleepMinder (I^2=0.0%; *p*=.945), ResMed S+ (I^2=0.0%; *p*=.659), and Beddit (I^2=0.0%; *p*=.833). Since only one study evaluated infrared camera, testing for heterogeneity was not relevant.

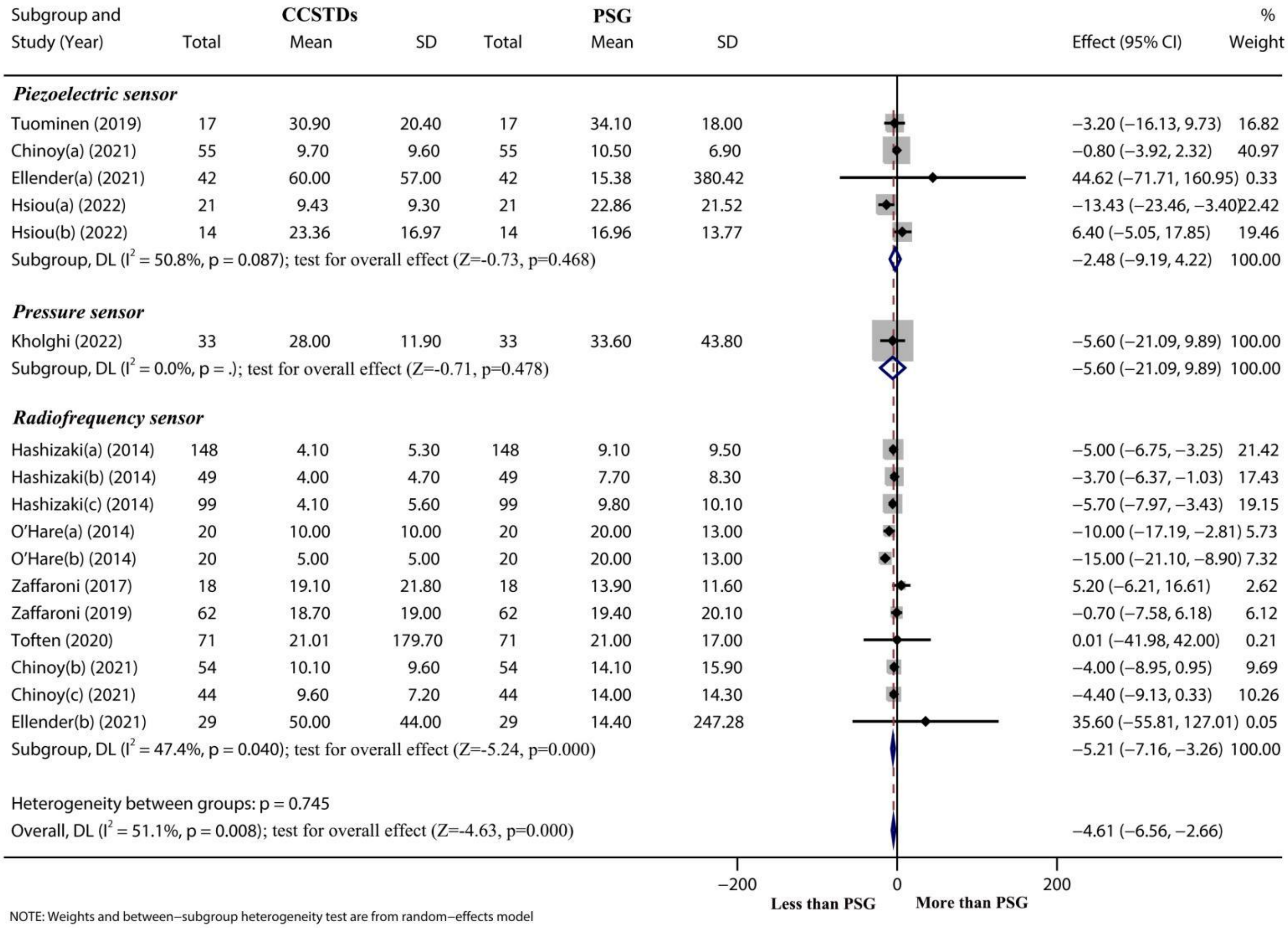

Figure S8. Forest plot meta-analyses for sensors subgroup – SOL (min).

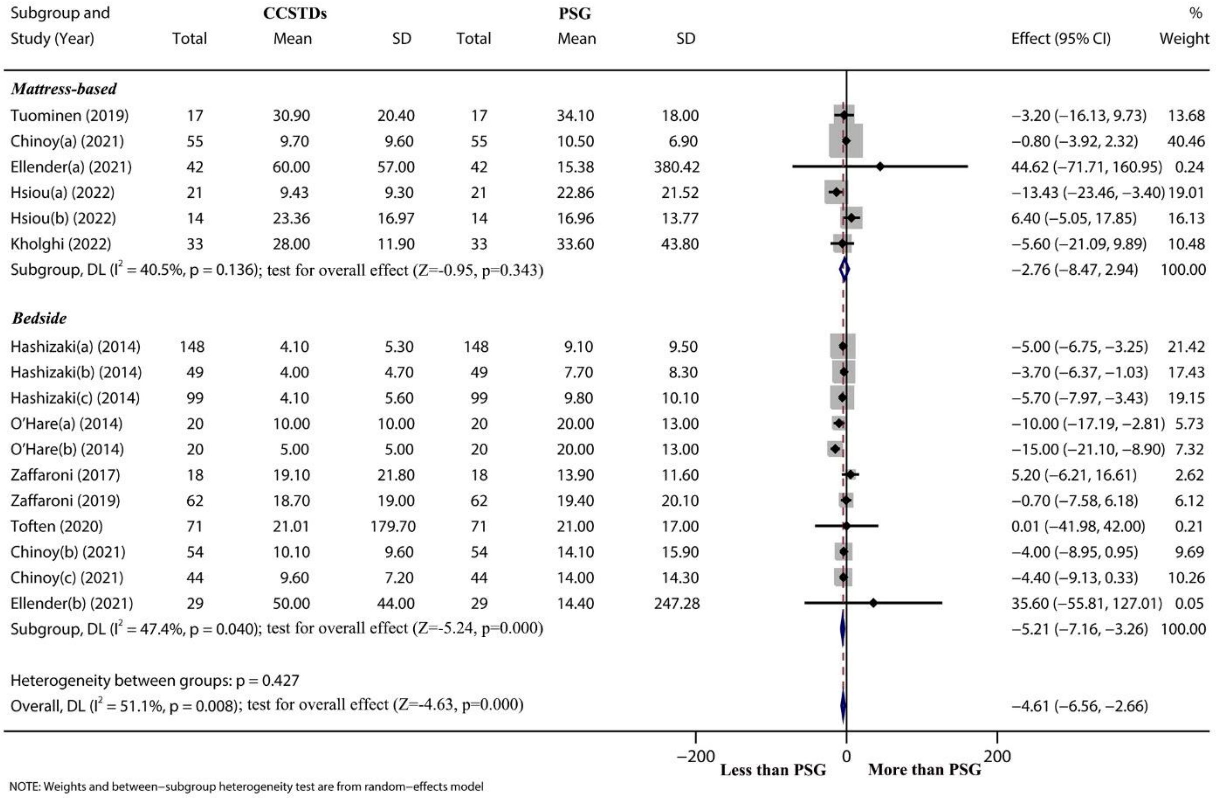

Figure S9. Forest plot meta-analyses for device types subgroup – SOL (min).

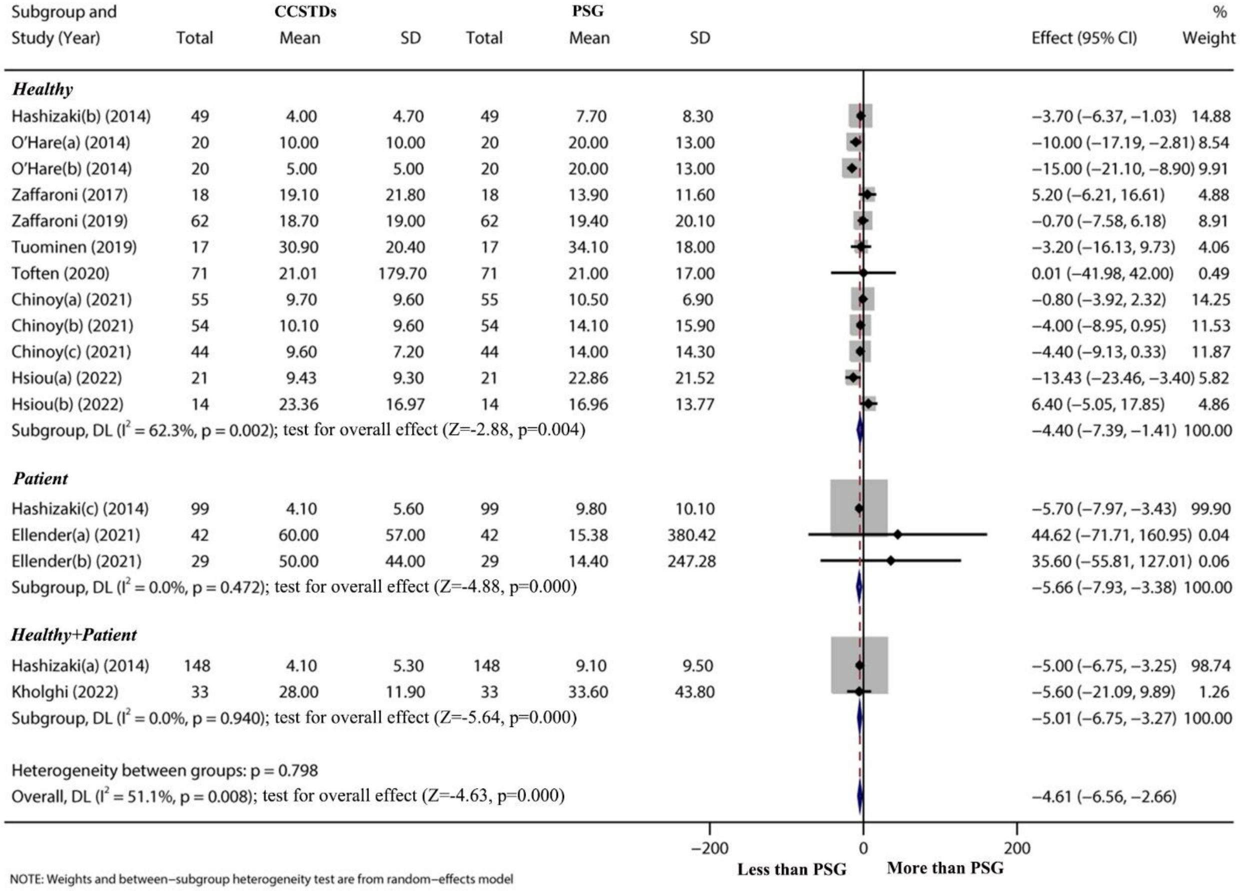

Figure S10. Forest plot meta-analyses for participant types subgroup – SOL (min).

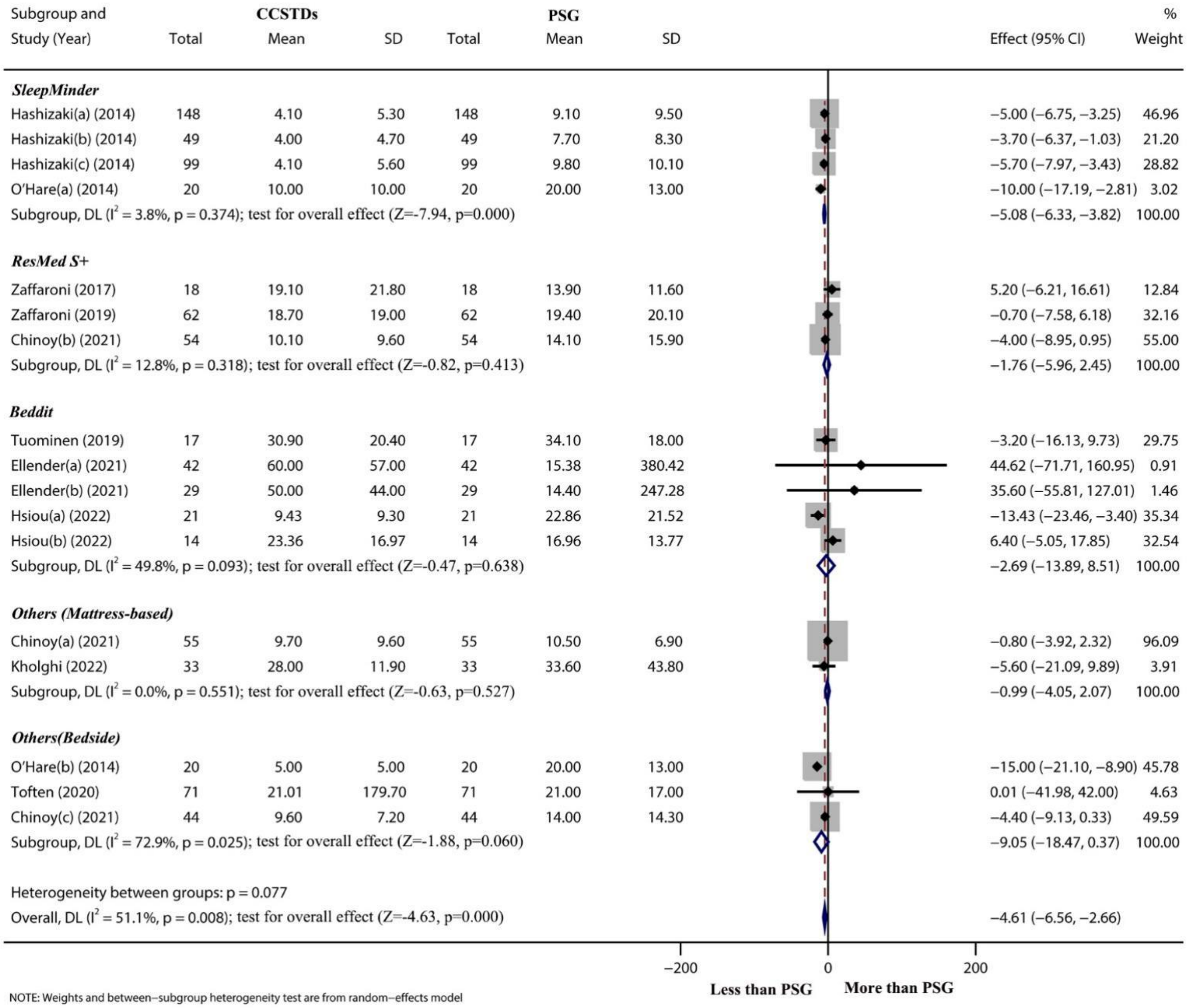

Figure S11. Forest plot meta-analyses for brands subgroup – SOL (min).

- SOL

Compared with PSG, the pooled estimate of effect size (see Figures S8 to S11) revealed a significant underestimation of radiofrequency sensor (N=11 studies; pooled mean=-5.21 min, 95% CI -7.16 to -3.26; P<.001), Bedside (N=11 studies; pooled mean=-4.61 min, 95% CI -6.56 to -2.66; P<.001), Healthy (N=12 studies; pooled mean=-4.4 min, 95% CI -7.39 to -1.41; P<.01), and SleepMinder (N=4 studies; pooled mean=-5.08 min, 95% CI -6.33 to -3.82; P<.001), while nonsignificant differences are shown in the estimation of piezoelectric sensor (N=5 studies; pooled mean=-2.48 min, 95% CI -9.19 to 4.22; P<.468), pressure sensor (N=1 study; pooled mean=-5.60 min, 95% CI -21.09 to 9.89; P<.478), mattress-based devices (hereinafter referred to as "Mattress-based") (N=6

studies; pooled mean=-2.76 min, 95% CI -8.47 to 2.94; P=.343), patient participants (hereinafter referred to as “Patient”) (N=3 studies; pooled mean=-5.66 min, 95% CI -7.93 to 3.38; P=.472), both healthy and patient participants (hereinafter referred to as “Healthy+ Patient”) (N=2 studies; pooled mean=-5.01 min, 95% CI -6.75 to 3.27; P=.478), ResMed S+ (N=3 studies; pooled mean =-1.76 min, 95% CI -5.96 to 2.45; P=.413), Beddit (N=5 studies; pooled mean=-2.69 min, 95% CI -13.89 to 8.51; P=.638), other brands of mattress device (hereinafter referred to as “Others (Mattress-based)”) (N=2 studies; pooled mean=-0.99 min, 95% CI -4.05 to 2.08; P=.527), and Others (Bedside) (N=3 studies; pooled mean=-9.05 min, 95% CI -18.47 to 0.37; P=.060). There was no significant heterogeneity in Mattress-Based (I^2=40.5%; p=.136), Patient (I^2=0.0%; p=.472), Healthy+Patient (I^2=0.0%; p=.940), SleepMinder (I^2=3.8%; p=.374), ResMed S+ (I^2=12.8 %; p=.318), and Others (Mattress-based) (I^2=0.0%; p=.551). Since only one study evaluated pressure sensor, testing for heterogeneity was not relevant.

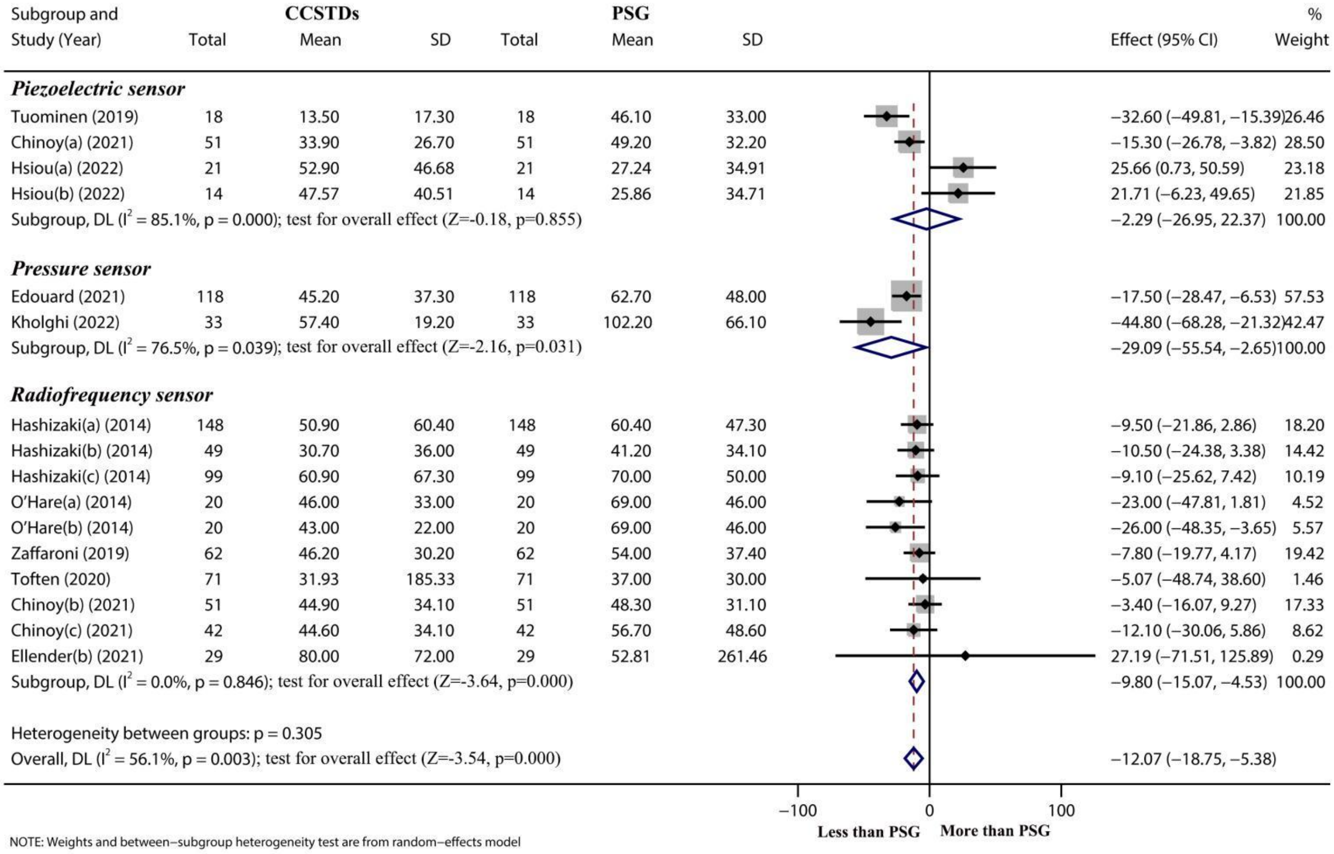

Figure S12. Forest plot meta-analyses for sensors subgroup -WASO (min).

Subgroup and Study (Year)	CCSTDs Total	CCSTDs Mean	CCSTDs SD	PSG Total	PSG Mean	PSG SD	Effect (95% CI)	% Weight
Mattress-based								
Tuominen (2019)	18	13.50	17.30	18	46.10	33.00	−32.60 (−49.81, −15.39)	17.57
Chinoy(a) (2021)	51	33.90	26.70	51	49.20	32.20	−15.30 (−26.78, −3.82)	19.68
Edouard (2021)	118	45.20	37.30	118	62.70	48.00	−17.50 (−28.47, −6.53)	19.85
Hsiou(a) (2022)	21	52.90	46.68	21	27.24	34.91	25.66 (0.73, 50.59)	14.49
Hsiou(b) (2022)	14	47.57	40.51	14	25.86	34.71	21.71 (−6.23, 49.65)	13.35
Kholghi (2022)	33	57.40	19.20	33	102.20	66.10	−44.80 (−68.28, −21.32)	15.06
Subgroup, DL (I^2 = 81.6%, p = 0.000); test for overall effect (Z=-1.47, p=0.141)							−12.34 (−28.76, 4.07)	100.00
Bedside								
Hashizaki(a) (2014)	148	50.90	60.40	148	60.40	47.30	−9.50 (−21.86, 2.86)	18.20
Hashizaki(b) (2014)	49	30.70	36.00	49	41.20	34.10	−10.50 (−24.38, 3.38)	14.42
Hashizaki(c) (2014)	99	60.90	67.30	99	70.00	50.00	−9.10 (−25.62, 7.42)	10.19
O'Hare(a) (2014)	20	46.00	33.00	20	69.00	46.00	−23.00 (−47.81, 1.81)	4.52
O'Hare(b) (2014)	20	43.00	22.00	20	69.00	46.00	−26.00 (−48.35, −3.65)	5.57
Zaffaroni (2019)	62	46.20	30.20	62	54.00	37.40	−7.80 (−19.77, 4.17)	19.42
Toften (2020)	71	31.93	185.33	71	37.00	30.00	−5.07 (−48.74, 38.60)	1.46
Chinoy(b) (2021)	51	44.90	34.10	51	48.30	31.10	−3.40 (−16.07, 9.27)	17.33
Chinoy(c) (2021)	42	44.60	34.10	42	56.70	48.60	−12.10 (−30.06, 5.86)	8.62
Ellender(b) (2021)	29	80.00	72.00	29	52.81	261.46	27.19 (−71.51, 125.89)	0.29
Subgroup, DL (I^2 = 0.0%, p = 0.846); test for overall effect (Z=-3.64, p=0.000)							−9.80 (−15.07, −4.53)	100.00
Heterogeneity between groups: p = 0.773								
Overall, DL (I^2 = 56.1%, p = 0.003); test for overall effect (Z=-3.54, p=0.000)							−12.07 (−18.75, −5.38)	

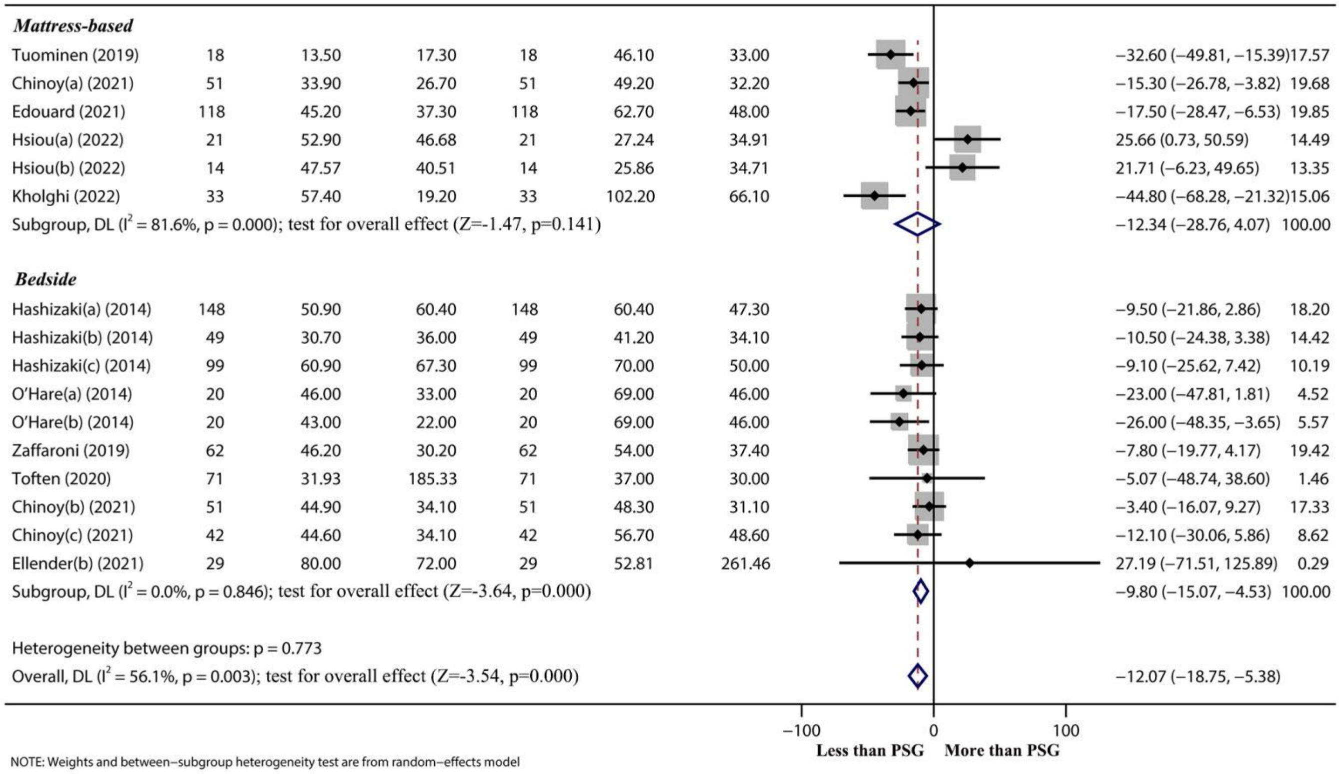

NOTE: Weights and between−subgroup heterogeneity test are from random−effects model

Figure S13. Forest plot meta-analyses for device types subgroup -WASO (min).

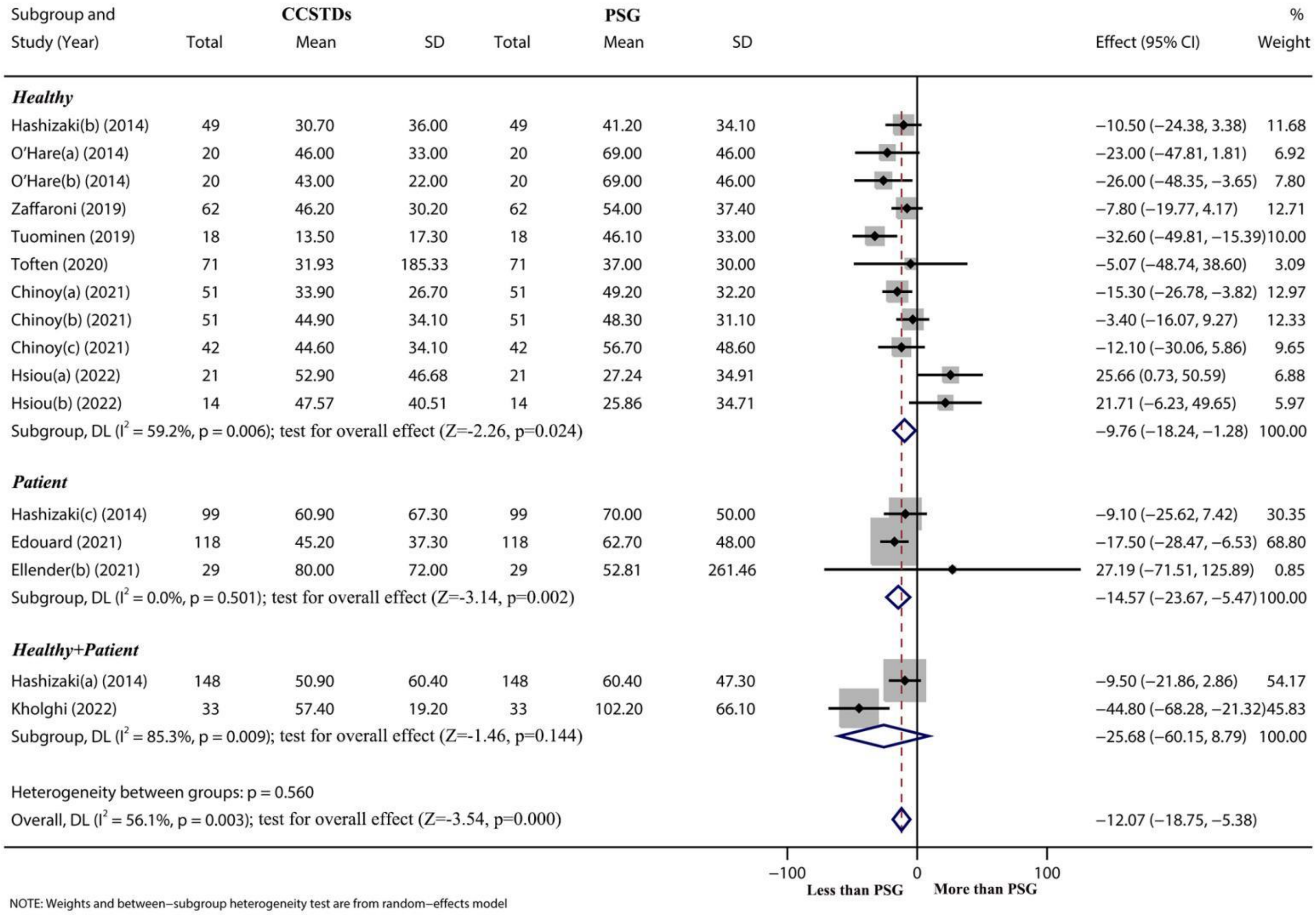

Figure S14. Forest plot meta-analyses for participants type subgroup – WASO (min).

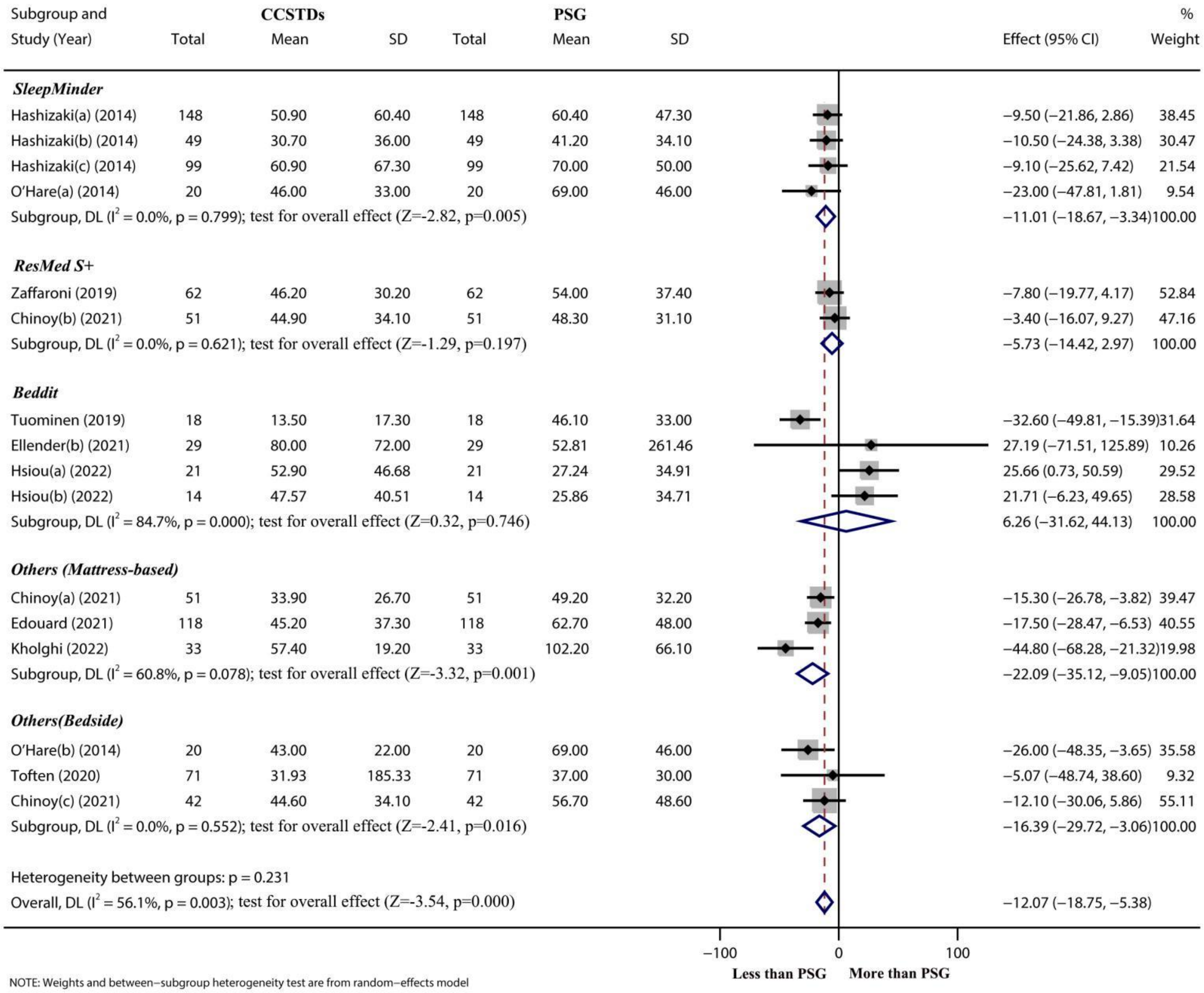

Figure S15. Forest plot meta-analyses for brands subgroup – WASO (min).

- WASO

The subgroup analyses in Figures S12 to S15 revealed general significant underestimation compared to PSG, but piezoelectric sensor (N=4 studies; pooled mean=-2.29 min, 95% CI -26.95 to 22.37; P=.855), Mattress-Based (N=6 studies; pooled mean=-12.34 min, 95% CI -28.76 to 4.07; P=.141), Healthy+Patient (N=2 studies; pooled mean =-25.68 min, 95% CI -60.15 to 8.79; P=.144), ResMed S+ (N=2 studies; pooled mean=-5.73 min, 95% CI -14.42 to 2.97; P=.197), and Beddit (N=4 studies; pooled mean=6.26 min, 95% CI -31.62 to 44.13; P=.746) showed a nonsignificant difference. There was no significant heterogeneity in Radiofrequency sensor (I^2=0.0%; p=.846), Bedside (I^2=0.0%; p=.846), Patients (I^2=0.0%; p=.501), SleepMinder (I^2=0.0%; p=.799), ResMed S+ (I^2=0.0%; p=.621), and Others (Bedside) (I^2=0.0%; p=0.552).

Subgroup and Study (Year)	CCSTDs Total	CCSTDs Mean	CCSTDs SD	PSG Total	PSG Mean	PSG SD	Effect (95% CI)	% Weight
Piezoelectric sensor								
Tuominen (2019)	18	90.90	6.70	18	83.60	7.90	7.30 (2.51, 12.09)	22.90
Stone (2020)	40	10.00	11.00	40	2.80	43.82	7.20 (−6.80, 21.20)	3.34
Chinoy(a) (2021)	51	90.80	6.10	51	87.80	7.30	3.00 (0.39, 5.61)	50.26
Ellender(a) (2021)	42	81.00	17.00	42	82.35	120.19	−1.35 (−38.06, 35.36)	0.50
Hsiou(a) (2022)	21	88.36	11.43	21	89.27	7.08	−0.91 (−6.66, 4.84)	17.03
Hsiou(b) (2022)	14	88.24	15.49	14	89.45	12.21	−1.21 (−11.54, 9.12)	5.97
Subgroup, DL (I^2 = 14.9%, p = 0.318); test for overall effect (Z=2.40, p=0.016)							3.19 (0.59, 5.79)	100.00
Pressure sensor								
Nagatomo (2020)	11	93.38	5.00	11	82.50	8.84	10.88 (4.88, 16.88)	46.95
Edouard (2021)	118	82.60	11.60	118	82.50	11.60	0.10 (−2.86, 3.06)	53.05
Subgroup, DL (I^2 = 90.0%, p = 0.002); test for overall effect (Z=0.96, p=0.337)							5.16 (−5.38, 15.71)	100.00
Radiofrequency sensor								
Hashizaki(a) (2014)	148	87.70	13.90	148	84.70	10.80	3.00 (0.16, 5.84)	12.59
Hashizaki(b) (2014)	49	91.90	8.60	49	88.80	9.00	3.10 (−0.39, 6.59)	9.93
Hashizaki(c) (2014)	99	85.60	15.40	99	82.60	11.10	3.00 (−0.74, 6.74)	9.07
O'Hare(a) (2014)	20	88.40	7.50	20	82.00	11.00	6.40 (0.57, 12.23)	4.66
O'Hare(b) (2014)	20	90.10	4.80	20	82.00	11.00	8.10 (2.84, 13.36)	5.52
De Chazal(a) (2011)	113	75.80	152.13	113	71.00	17.00	4.80 (−23.42, 33.02)	0.24
De Chazal(b) (2011)	50	80.00	98.24	50	73.00	17.00	7.00 (−20.63, 34.63)	0.25
De Chazal(c) (2011)	63	70.90	112.90	63	69.00	18.00	1.90 (−26.33, 30.13)	0.24
Pallin(a) (2014)	103	81.20	159.82	103	80.40	13.10	0.80 (−30.17, 31.77)	0.20
Pallin(b) (2014)	18	85.80	30.65	18	80.30	11.30	5.50 (−9.59, 20.59)	0.82
Pallin(c) (2014)	36	88.20	80.28	36	84.80	10.80	3.40 (−23.06, 29.86)	0.27
Pallin(d) (2014)	21	70.90	95.36	21	77.80	16.00	−6.90 (−48.26, 34.46)	0.11
Pallin(e) (2014)	28	77.10	86.23	28	77.00	13.70	0.10 (−32.24, 32.44)	0.18
Terjung (2016)	57	91.80	8.30	57	83.70	12.80	8.10 (4.14, 12.06)	8.40
Zaffaroni (2017)	18	92.90	3.70	18	93.10	3.70	−0.20 (−2.62, 2.22)	14.70
Toften (2020)	71	89.02	36.31	71	88.00	8.00	1.02 (−7.63, 9.67)	2.35
Feng (2020)	40	88.98	7.85	40	88.58	8.24	0.40 (−3.13, 3.93)	9.78
Chinoy(b) (2021)	51	88.00	8.30	51	88.00	7.00	0.00 (−2.98, 2.98)	11.94
Chinoy(c) (2021)	42	87.80	7.80	42	86.20	10.20	1.60 (−2.28, 5.48)	8.62
Ellender(b) (2021)	29	68.80	21.00	29	84.68	100.66	−15.88 (−53.30, 21.54)	0.14
Subgroup, DL (I^2 = 22.6%, p = 0.176); test for overall effect (Z=3.69, p=0.000)							2.61 (1.23, 4.00)	100.00
Heterogeneity between groups: p = 0.842								
Overall, DL (I^2 = 34.2%, p = 0.041); test for overall effect (Z=4.36, p=0.000)							2.88 (1.58, 4.17)	

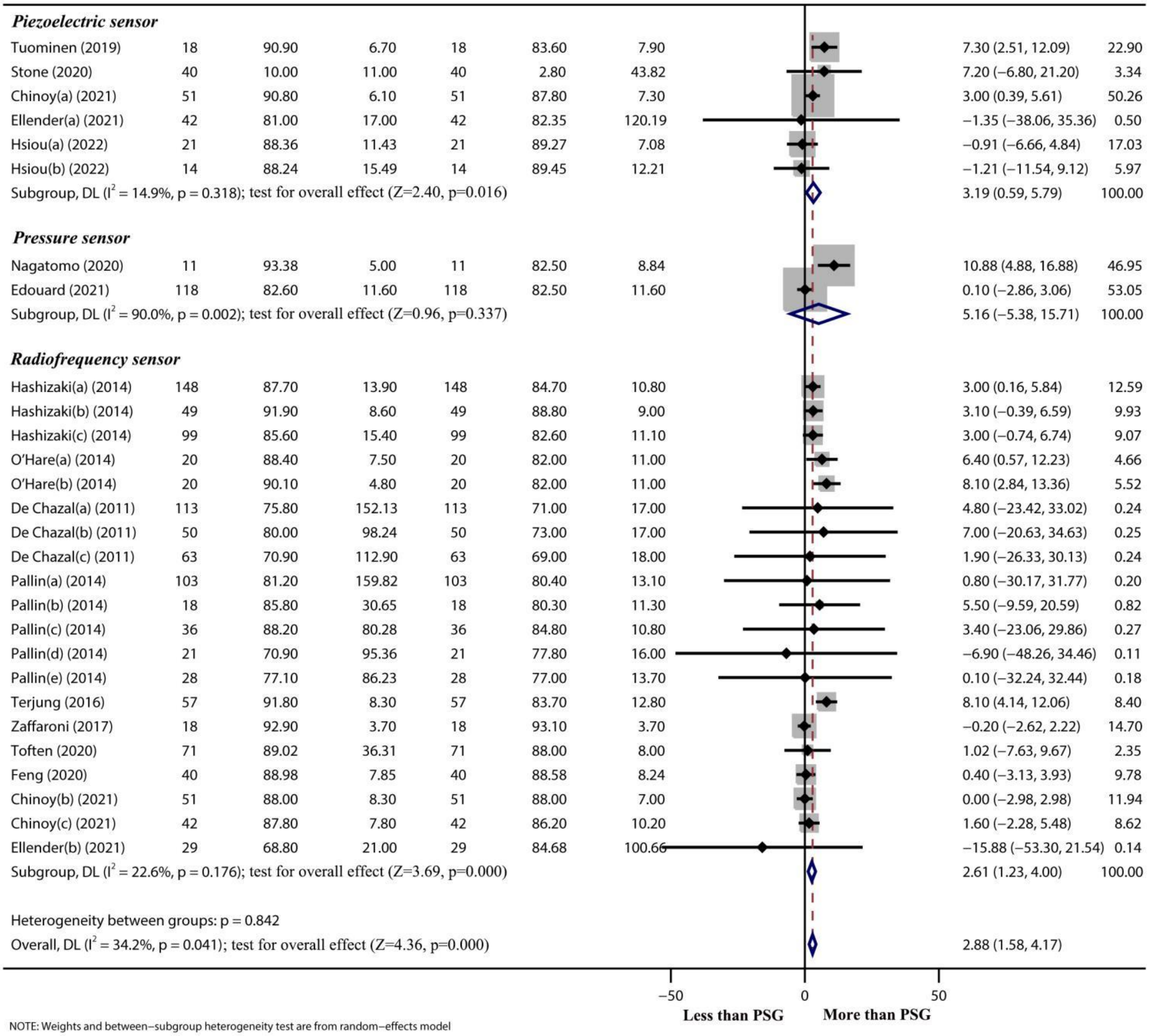

NOTE: Weights and between−subgroup heterogeneity test are from random−effects model

Figure S16. Forest plot meta-analyses for sensors subgroup - SE (%).

Subgroup and Study (Year)	CCSTDs Total	CCSTDs Mean	CCSTDs SD	PSG Total	PSG Mean	PSG SD	Effect (95% CI)	% Weight
Mattress-based								
Tuominen (2019)	18	90.90	6.70	18	83.60	7.90	7.30 (2.51, 12.09)	16.44
Stone (2020)	40	10.00	11.00	40	2.80	43.82	7.20 (−6.80, 21.20)	4.02
Nagatomo (2020)	11	93.38	5.00	11	82.50	8.84	10.88 (4.88, 16.88)	13.33
Chinoy(a) (2021)	51	90.80	6.10	51	87.80	7.30	3.00 (0.39, 5.61)	23.05
Edouard (2021)	118	82.60	11.60	118	82.50	11.60	0.10 (−2.86, 3.06)	21.98
Ellender(a) (2021)	42	81.00	17.00	42	82.35	120.19	−1.35 (−38.06, 35.36)	0.67
Hsiou(a) (2022)	21	88.36	11.43	21	89.27	7.08	−0.91 (−6.66, 4.84)	13.92
Hsiou(b) (2022)	14	88.24	15.49	14	89.45	12.21	−1.21 (−11.54, 9.12)	6.59
Subgroup, DL (I^2 = 56.7%, p = 0.023) ; test for overall effect (Z=2.22, p=0.027)							3.44 (0.40, 6.48)	100.00
Bedside								
Hashizaki(a) (2014)	148	87.70	13.90	148	84.70	10.80	3.00 (0.16, 5.84)	12.59
Hashizaki(b) (2014)	49	91.90	8.60	49	88.80	9.00	3.10 (−0.39, 6.59)	9.93
Hashizaki(c) (2014)	99	85.60	15.40	99	82.60	11.10	3.00 (−0.74, 6.74)	9.07
O'Hare(a) (2014)	20	88.40	7.50	20	82.00	11.00	6.40 (0.57, 12.23)	4.66
O'Hare(b) (2014)	20	90.10	4.80	20	82.00	11.00	8.10 (2.84, 13.36)	5.52
De Chazal(a) (2011)	113	75.80	152.13	113	71.00	17.00	4.80 (−23.42, 33.02)	0.24
De Chazal(b) (2011)	50	80.00	98.24	50	73.00	17.00	7.00 (−20.63, 34.63)	0.25
De Chazal(c) (2011)	63	70.90	112.90	63	69.00	18.00	1.90 (−26.33, 30.13)	0.24
Pallin(a) (2014)	103	81.20	159.82	103	80.40	13.10	0.80 (−30.17, 31.77)	0.20
Pallin(b) (2014)	18	85.80	30.65	18	80.30	11.30	5.50 (−9.59, 20.59)	0.82
Pallin(c) (2014)	36	88.20	80.28	36	84.80	10.80	3.40 (−23.06, 29.86)	0.27
Pallin(d) (2014)	21	70.90	95.36	21	77.80	16.00	−6.90 (−48.26, 34.46)	0.11
Pallin(e) (2014)	28	77.10	86.23	28	77.00	13.70	0.10 (−32.24, 32.44)	0.18
Terjung (2016)	57	91.80	8.30	57	83.70	12.80	8.10 (4.14, 12.06)	8.40
Zaffaroni (2017)	18	92.90	3.70	18	93.10	3.70	−0.20 (−2.62, 2.22)	14.70
Toften (2020)	71	89.02	36.31	71	88.00	8.00	1.02 (−7.63, 9.67)	2.35
Feng (2020)	40	88.98	7.85	40	88.58	8.24	0.40 (−3.13, 3.93)	9.78
Chinoy(b) (2021)	51	88.00	8.30	51	88.00	7.00	0.00 (−2.98, 2.98)	11.94
Chinoy(c) (2021)	42	87.80	7.80	42	86.20	10.20	1.60 (−2.28, 5.48)	8.62
Ellender(b) (2021)	29	68.80	21.00	29	84.68	100.66	−15.88 (−53.30, 21.54)	0.14
Subgroup, DL (I^2 = 22.6%, p = 0.176) ; test for overall effect (Z=3.69, p=0.000)							2.61 (1.23, 4.00)	100.00
Heterogeneity between groups: p = 0.629								
Overall, DL (I^2 = 34.2%, p = 0.041) ; test for overall effect (Z=4.36, p=0.000)							2.88 (1.58, 4.17)	

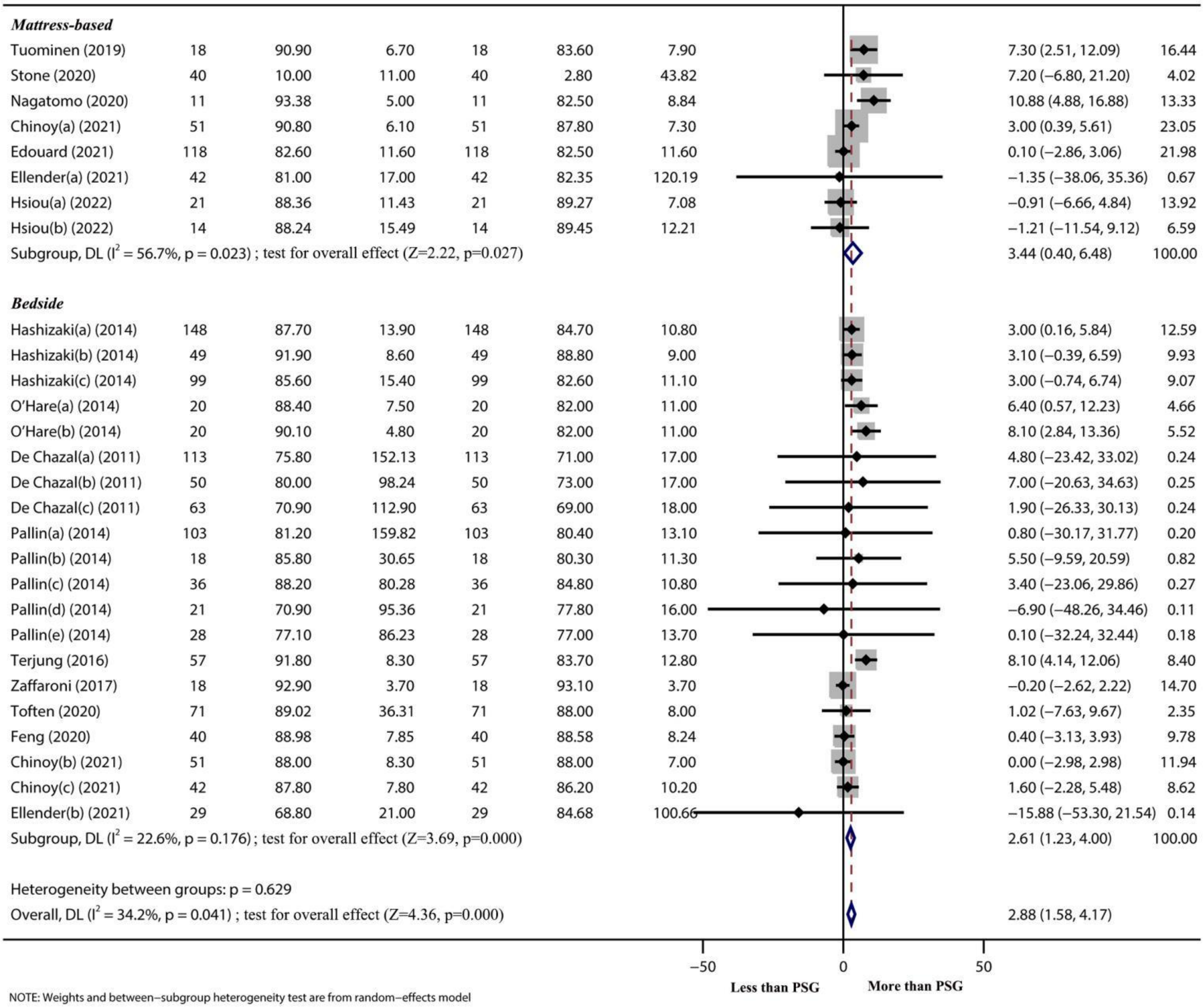

NOTE: Weights and between−subgroup heterogeneity test are from random−effects model

Figure S17. Forest plot meta-analyses for device types subgroup - SE (%)

Subgroup and Study (Year)	CCSTDs Total	CCSTDs Mean	CCSTDs SD	PSG Total	PSG Mean	PSG SD	Effect (95% CI)	% Weight
Healthy								
Hashizaki(b) (2014)	49	91.90	8.60	49	88.80	9.00	3.10 (−0.39, 6.59)	11.43
O'Hare(a) (2014)	20	88.40	7.50	20	82.00	11.00	6.40 (0.57, 12.23)	5.69
O'Hare(b) (2014)	20	90.10	4.80	20	82.00	11.00	8.10 (2.84, 13.36)	6.66
De Chazal(b) (2011)	50	80.00	98.24	50	73.00	17.00	7.00 (−20.63, 34.63)	0.32
Pallin(b) (2014)	18	85.80	30.65	18	80.30	11.30	5.50 (−9.59, 20.59)	1.04
Pallin(c) (2014)	36	88.20	80.28	36	84.80	10.80	3.40 (−23.06, 29.86)	0.35
Zaffaroni (2017)	18	92.90	3.70	18	93.10	3.70	−0.20 (−2.62, 2.22)	16.11
Tuominen (2019)	18	90.90	6.70	18	83.60	7.90	7.30 (2.51, 12.09)	7.64
Stone (2020)	40	10.00	11.00	40	2.80	43.82	7.20 (−6.80, 21.20)	1.21
Toften (2020)	71	89.02	36.31	71	88.00	8.00	1.02 (−7.63, 9.67)	2.94
Chinoy(a) (2021)	51	90.80	6.10	51	87.80	7.30	3.00 (0.39, 5.61)	15.15
Chinoy(b) (2021)	51	88.00	8.30	51	88.00	7.00	0.00 (−2.98, 2.98)	13.45
Chinoy(c) (2021)	42	87.80	7.80	42	86.20	10.20	1.60 (−2.28, 5.48)	10.07
Hsiou(a) (2022)	21	88.36	11.43	21	89.27	7.08	−0.91 (−6.66, 4.84)	5.82
Hsiou(b) (2022)	14	88.24	15.49	14	89.45	12.21	−1.21 (−11.54, 9.12)	2.13
Subgroup, DL (I^2 = 30.6%, p = 0.125); test for overall effect (Z=3.15, p=0.002)							2.53 (0.95, 4.10)	100.00
Patient								
Hashizaki(c) (2014)	99	85.60	15.40	99	82.60	11.10	3.00 (−0.74, 6.74)	19.86
De Chazal(c) (2011)	63	70.90	112.90	63	69.00	18.00	1.90 (−26.33, 30.13)	1.27
Pallin(d) (2014)	21	70.90	95.36	21	77.80	16.00	−6.90 (−48.26, 34.46)	0.61
Pallin(e) (2014)	28	77.10	86.23	28	77.00	13.70	0.10 (−32.24, 32.44)	0.98
Terjung (2016)	57	91.80	8.30	57	83.70	12.80	8.10 (4.14, 12.06)	19.25
Nagatomo (2020)	11	93.38	5.00	11	82.50	8.84	10.88 (4.88, 16.88)	14.06
Feng (2020)	40	88.98	7.85	40	88.58	8.24	0.40 (−3.13, 3.93)	20.46
Edouard (2021)	118	82.60	11.60	118	82.50	11.60	0.10 (−2.86, 3.06)	22.02
Ellender(a) (2021)	42	81.00	17.00	42	82.35	120.19	−1.35 (−38.06, 35.36)	0.76
Ellender(b) (2021)	29	68.80	21.00	29	84.68	100.66	−15.88 (−53.30, 21.54)	0.74
Subgroup, DL (I^2 = 55.1%, p = 0.018); test for overall effect (Z=2.19, p=0.028)							3.64 (0.39, 6.90)	100.00
Healthy+Patient								
Hashizaki(a) (2014)	148	87.70	13.90	148	84.70	10.80	3.00 (0.16, 5.84)	98.19
De Chazal(a) (2011)	113	75.80	152.13	113	71.00	17.00	4.80 (−23.42, 33.02)	0.99
Pallin(a) (2014)	103	81.20	159.82	103	80.40	13.10	0.80 (−30.17, 31.77)	0.82
Subgroup, DL (I^2 = 0.0%, p = 0.983); test for overall effect (Z=2.09, p=0.036)							3.00 (0.19, 5.81)	100.00
Heterogeneity between groups: p = 0.822								
Overall, DL (I^2 = 34.2%, p = 0.041); test for overall effect (Z=4.36, p=0.000)							2.88 (1.58, 4.17)	

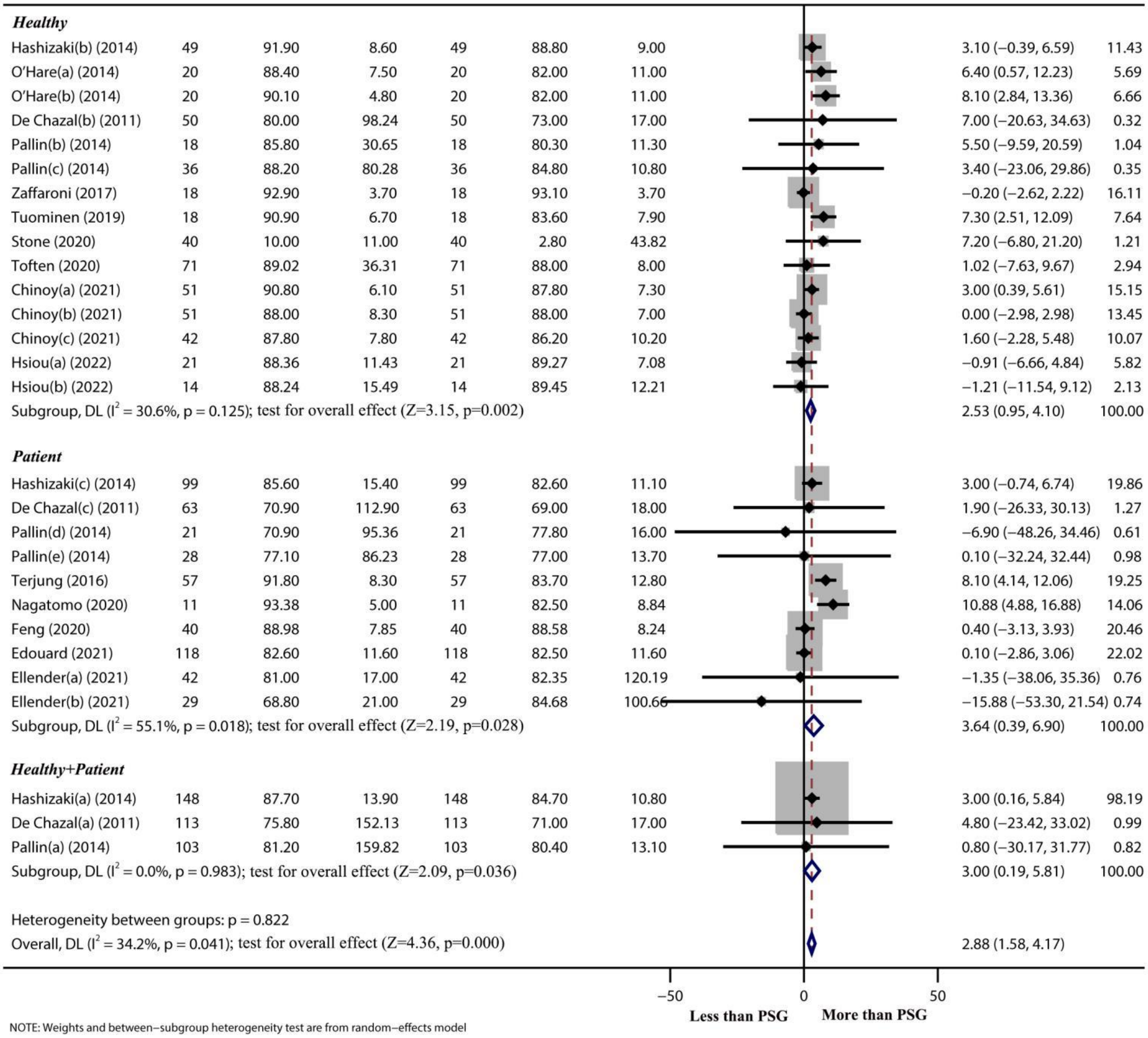

NOTE: Weights and between−subgroup heterogeneity test are from random−effects model

Figure S18. Forest plot meta-analyses for participant types subgroup - SE (%).

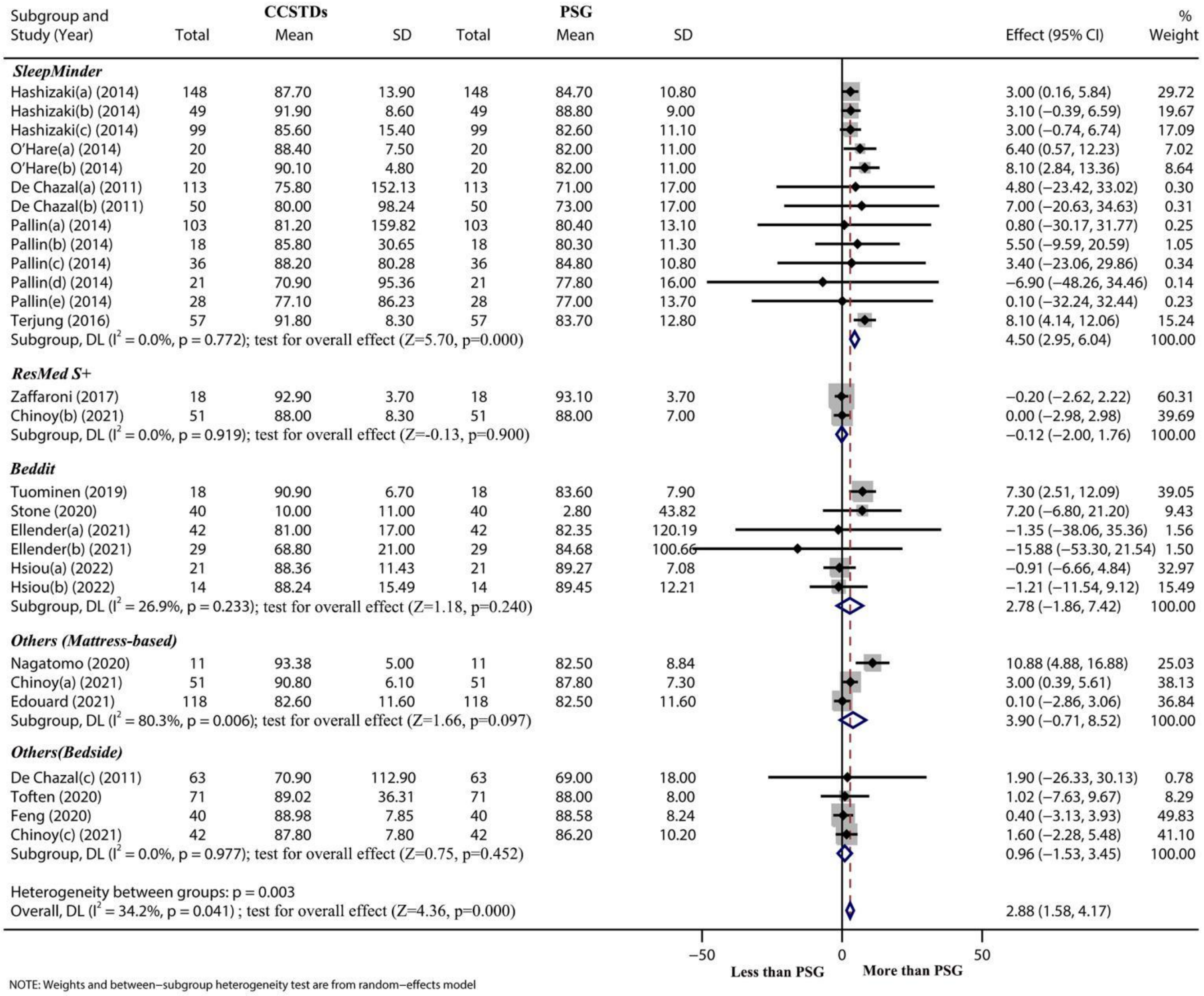

Figure S19. Forest plot meta-analyses for brands subgroup - SE (%).

- SE

Analysis of three subgroups in Figures S16 to S19 revealed significant overestimation except for pressure sensor (N=2 studies; pooled mean=5.16 min, 95% CI -5.38 to 15.71; P=.337) compared to PSG. The results of the subgroup analyses (see Figure S19) for the brand of devices showed no significant difference except for SleepMinder (N=13 studies; pooled mean=4.50 min, 95% CI 2.95 to 6.04; P<.001) which was a significant difference. The significant heterogeneity was detected only in pressure sensor (I^2=90.0%; P=.002), Mattress-Based (I^2=56.7%; P=.023), Patient (I^2=55.1%; p=.018), and Others (Mattress-based) (I^2=80.3%; p=.006).

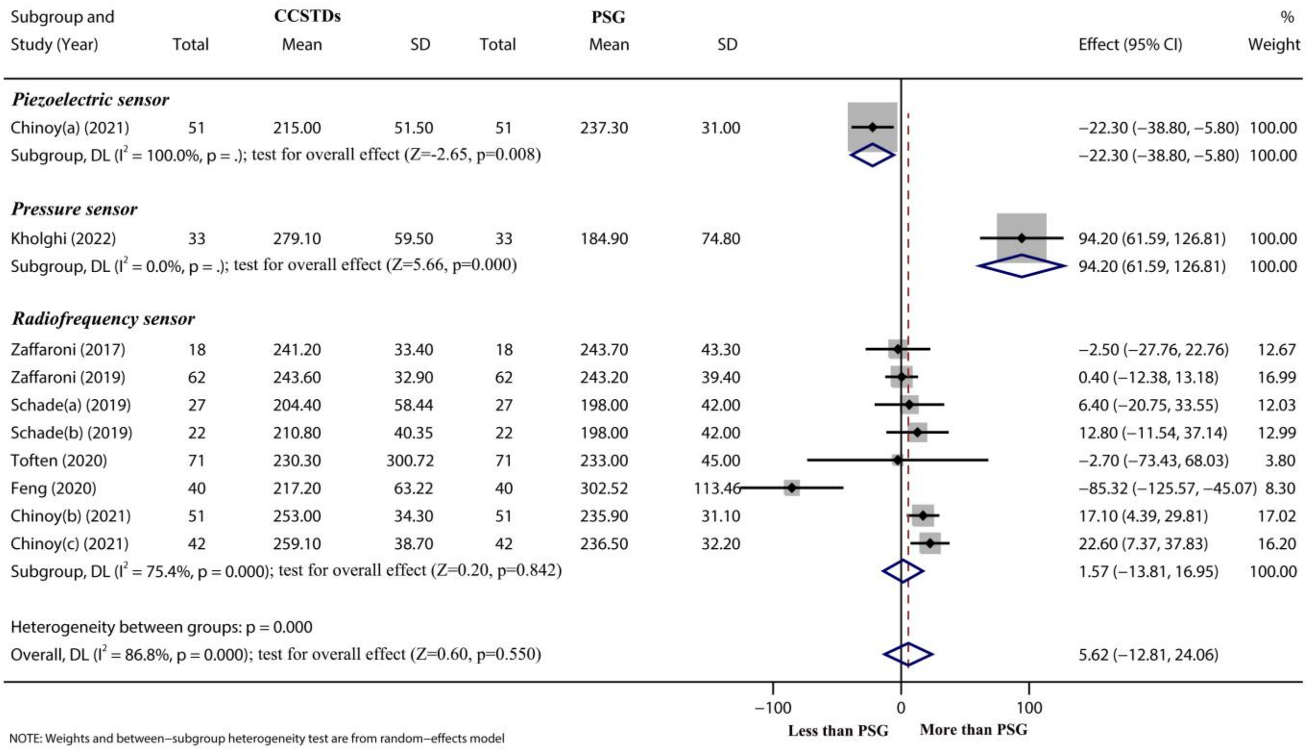

Figure S20. Forest plot meta-analyses for sensors subgroup - light sleep(min).

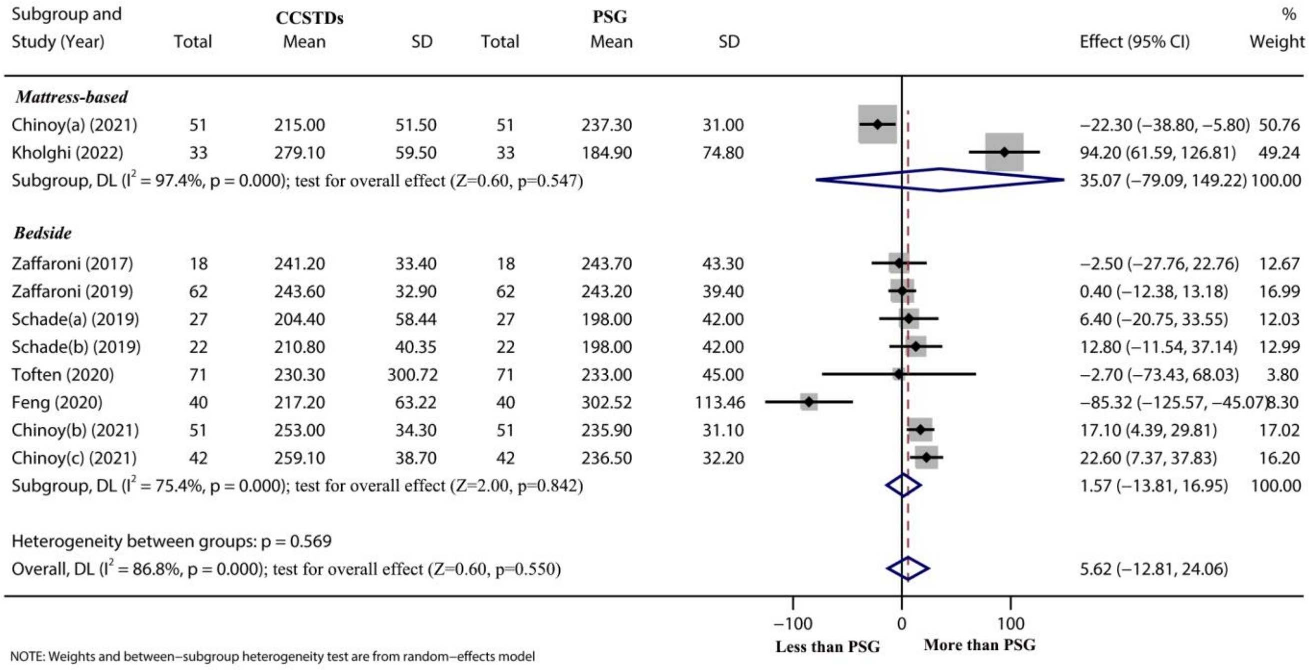

Figure S21. Forest plot meta-analyses for devices type subgroup - light sleep(min).

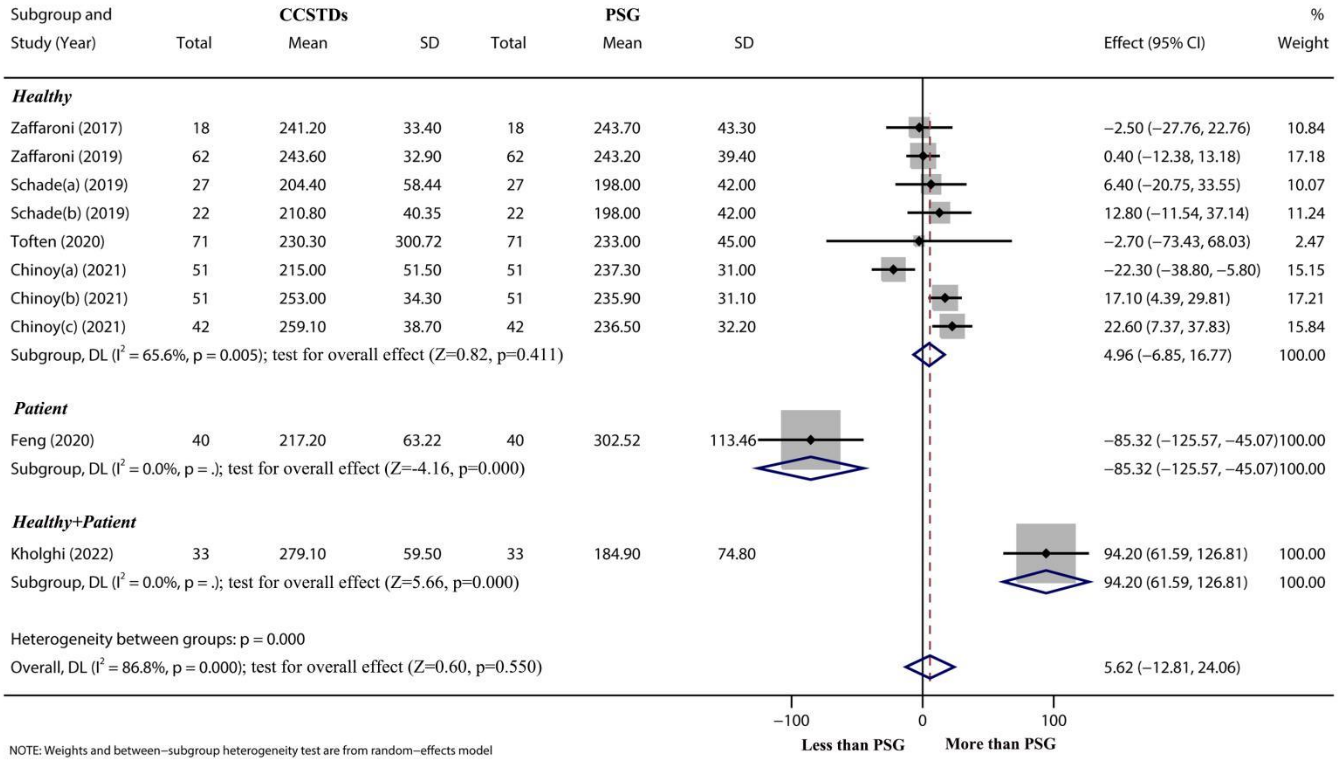

Figure S22. Forest plot meta-analyses for participant types subgroup - light sleep(min).

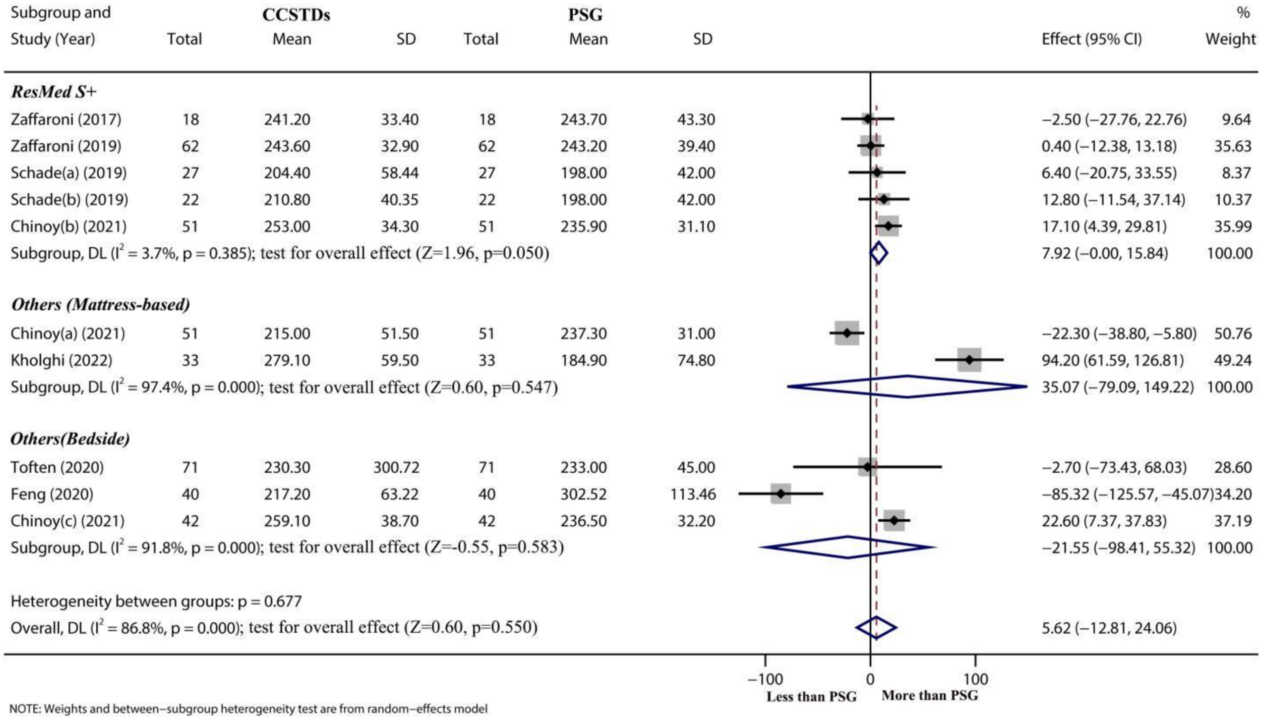

Figure S23. Forest plot meta-analyses for brands subgroup - light sleep(min).

- Light Sleep

Compared with PSG, the pooled estimate of effect size in Figures S20 to S23 revealed the following: nonsignificant difference in the estimation of radiofrequency sensor (n=8 studies; pooled mean=1.57 min, 95% CI -13.81 to 16.95; P=.842), Mattress-Based (n=2 studies; pooled mean=35.07 min, 95% CI -79.09 to 149.22; P=.547), Bedside (n=8 studies; pooled mean=1.57 min, 95% CI -13.81 to 16.95; P=.842), Healthy (n=8 studies; pooled mean=4.96 min, 95% CI -6.86 to 16.78; P=.404), and the subgroup of brands; significant underestimation of piezoelectric sensor (n=1 study; pooled mean=-22.30 min, 95% CI -38.80 to -5.80; P<.01), Patient (n=1 study; pooled mean=-85.32 min, 95% CI -125.57 to -45.07; P<.001); significant overestimation of pressure sensor (n=1 study; pooled mean =94.20 min, 95% CI 61.59 to 126.81; P<.001), Healthy+Patient (n=1 study; pooled mean =94.20 min, 95% CI 61.59 to 126.81; P<.001). The nonsignificant heterogeneity was only detected in ResMed S+ (I^2=3.7%; *p*=.385). Since only one study evaluated piezoelectric sensor, pressure sensor, Patient and Healthy+Patient, testing for heterogeneity was not relevant.

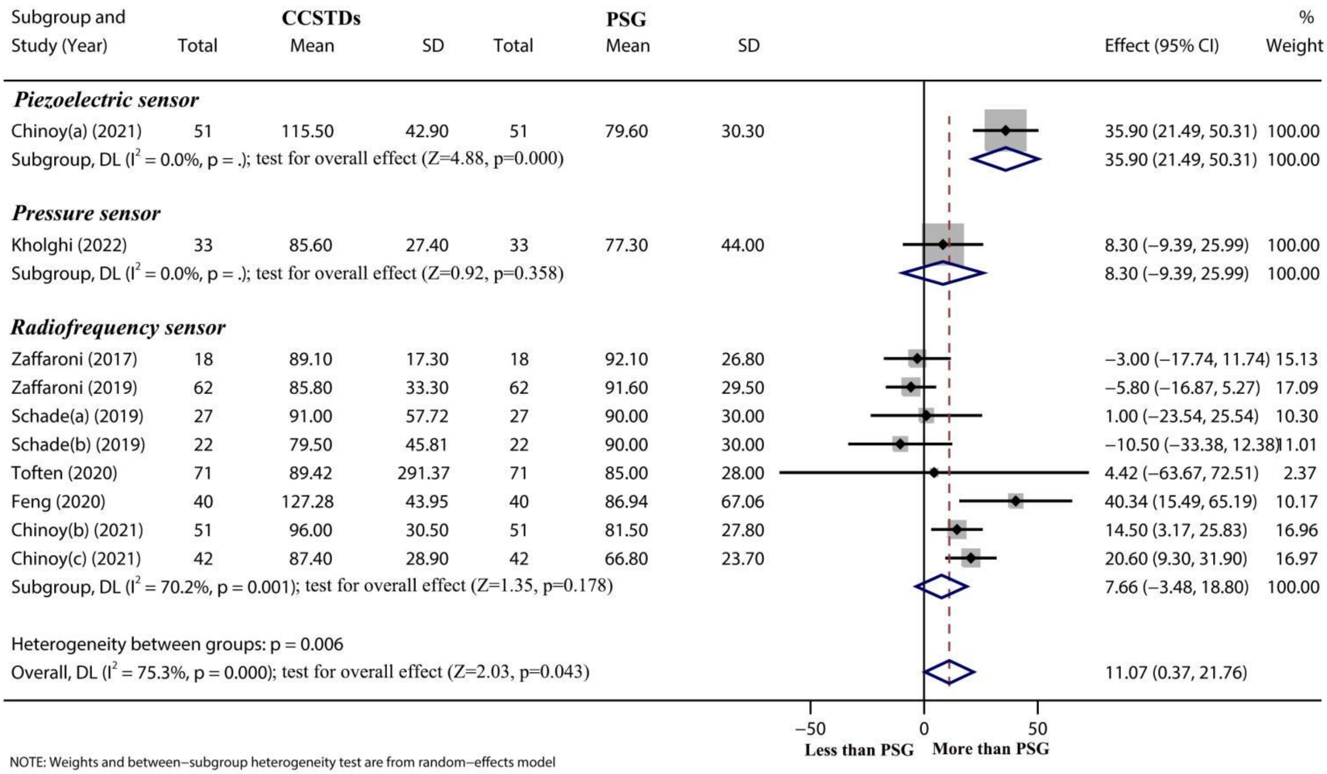

Figure S24. Forest plot meta-analyses for sensors subgroup - deep sleep(min).

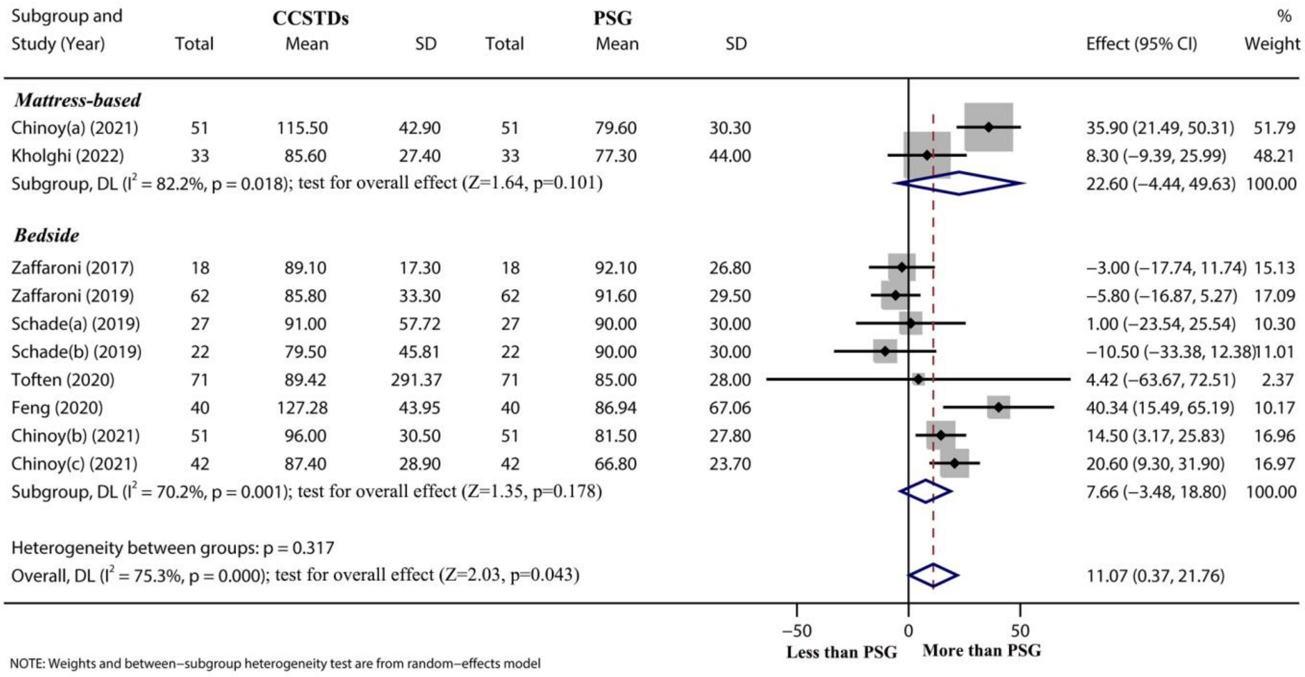

Figure S25. Forest plot meta-analyses for device types subgroup - deep sleep(min).

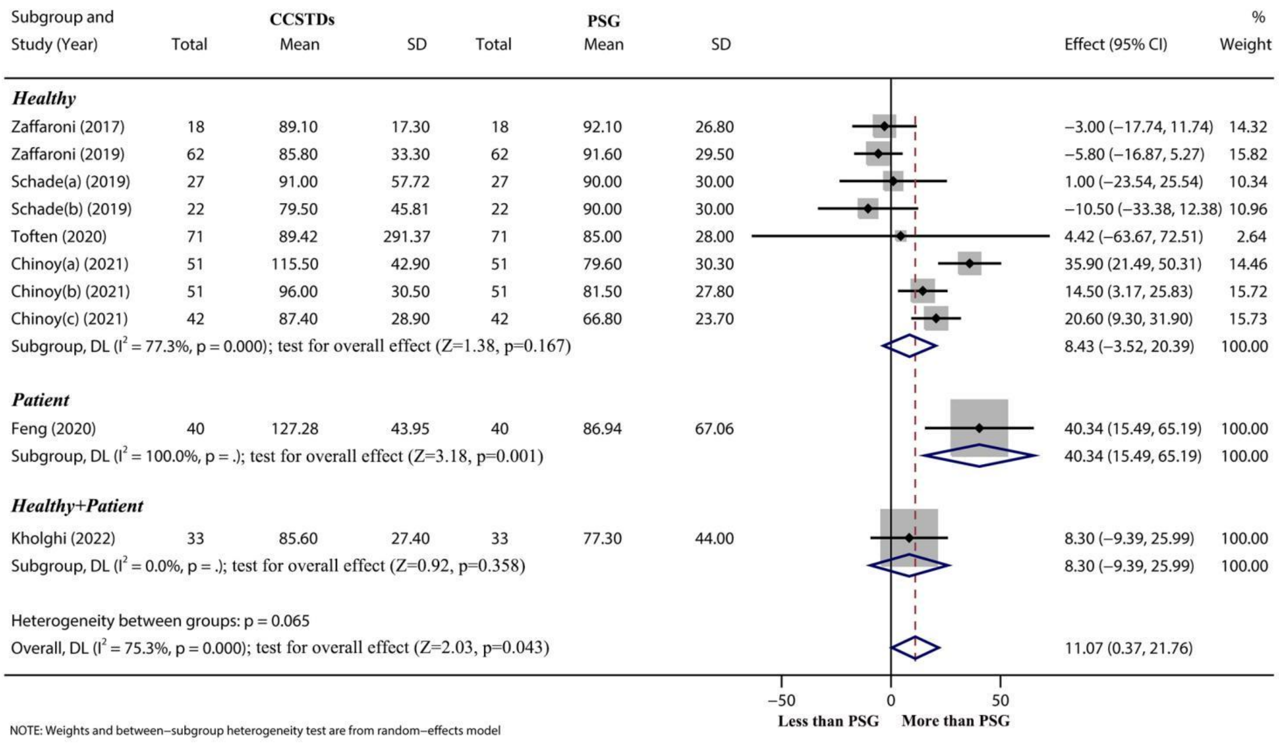

Figure S26. Forest plot meta-analyses for participant types subgroup - deep sleep(min).

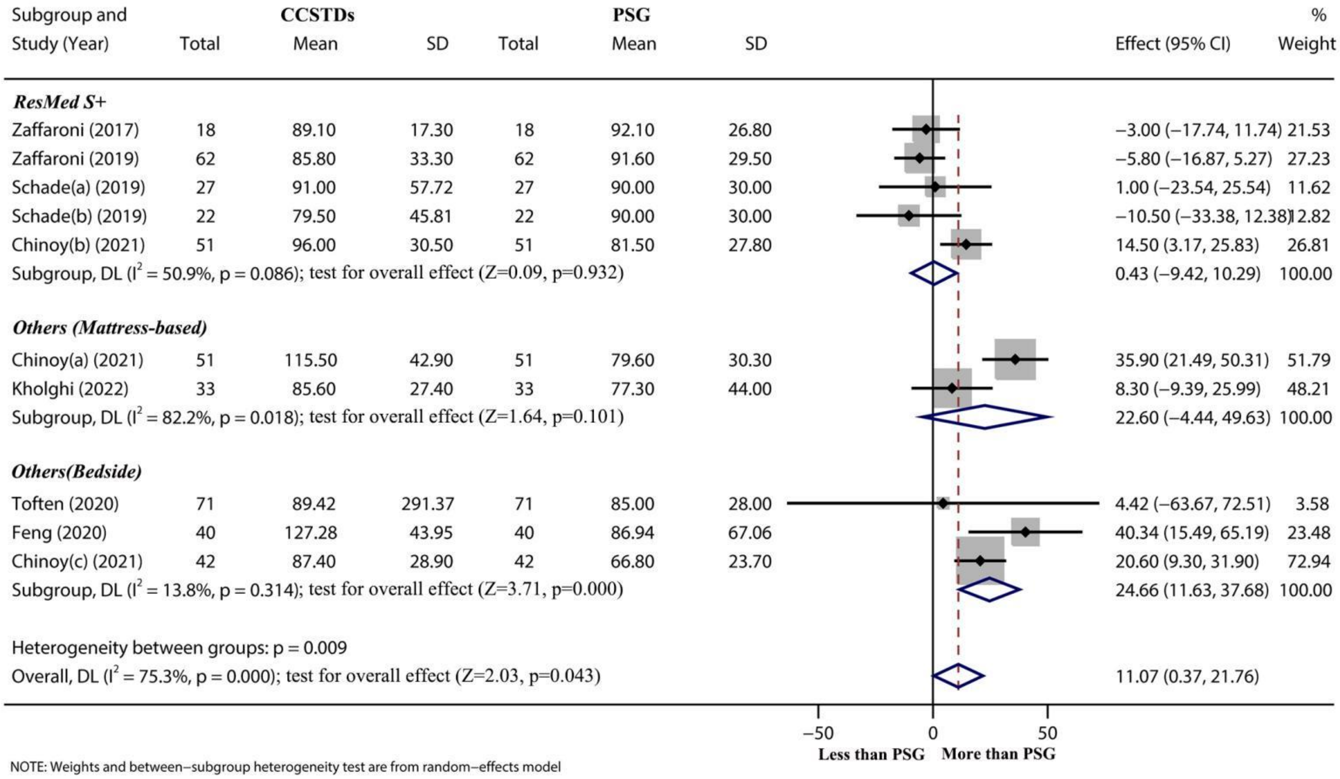

Figure S27. Forest plot meta-analyses for brands subgroup - deep sleep(min).

- Deep Sleep

Compared with PSG, the pooled estimate of effect size in Figures S24 to S27 revealed no significant difference in pressure sensor (n=1 study; pooled mean=8.30 min, 95% CI -9.39 to 25.99; P=.358), radiofrequency sensor (n=8 studies; pooled mean=7.66 min, 95% CI -3.48 to 18.80; P=.178), Healthy (n=8 studies; pooled mean=8.43 min, 95% CI -3.52 to 20.39; P=.167), Healthy+Patients (n=1 study; pooled mean=8.30min, 95% CI -9.39 to 25.99; P=.358), ResMed S+ (n=5 studies; pooled mean=0.43 min, 95% CI -9.43 to 10.29; P=.932), Others (Mattress-based) (n=2 studies; pooled mean=22.60 min, 95% CI -4.44 to 49.63; P=.101), and the subgroup of type of device. The significant overestimation was detected in piezoelectric sensor (n=1 study; pooled mean=35.9 min, 95% CI 21.49 to 50.31; P<.001), Patient (n=1 study; pooled mean=40.34 min, 95% CI 15.49 to 65.19; P<.001), and Others (Bedside) (n=3 studies; pooled mean=24.66 min, 95% CI 11.64 to 37.68; P<.001). There was no significant heterogeneity only in Others (Bedside) (I^2=13.8%; P=.314). Since only one study evaluated piezoelectric sensor, pressure sensor, Patient, and Healthy+Patient, testing for heterogeneity was not relevant.

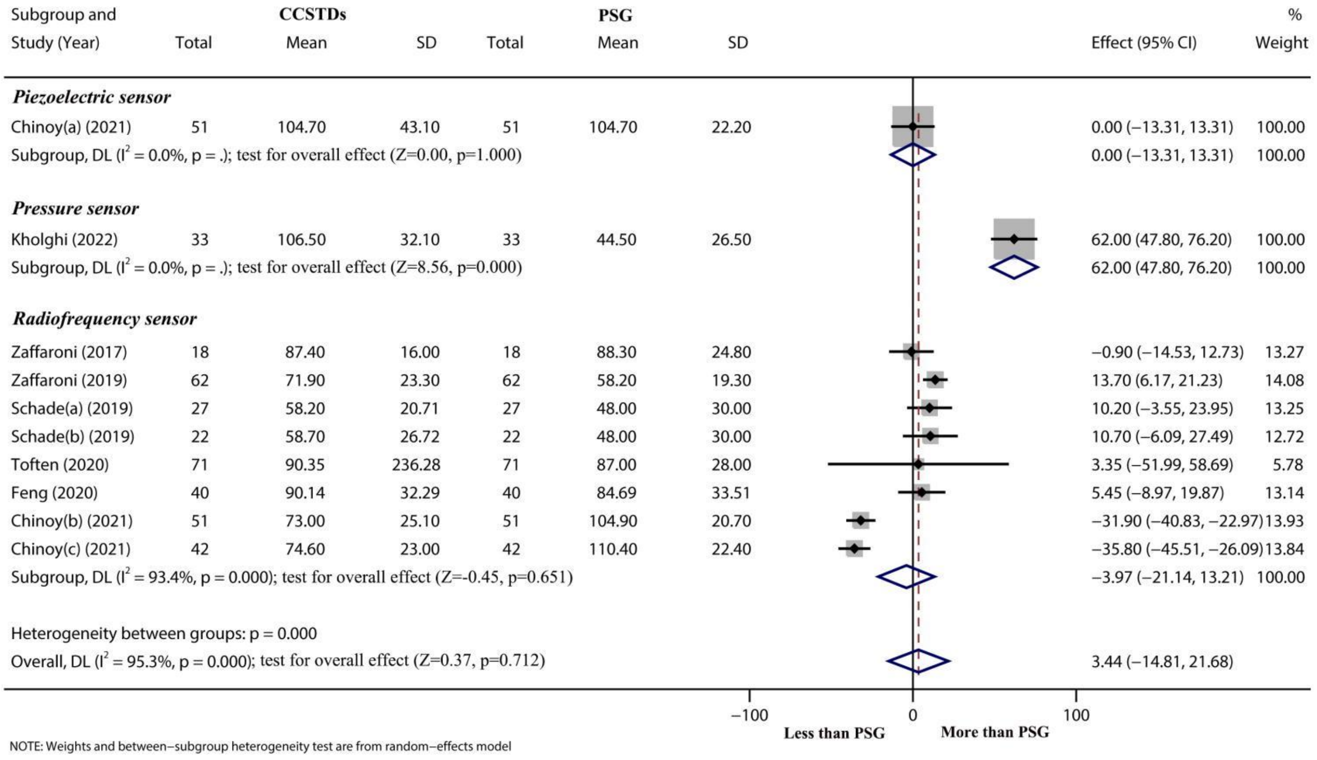

Figure S28. Forest plot meta-analyses for sensors subgroup - REM(min).

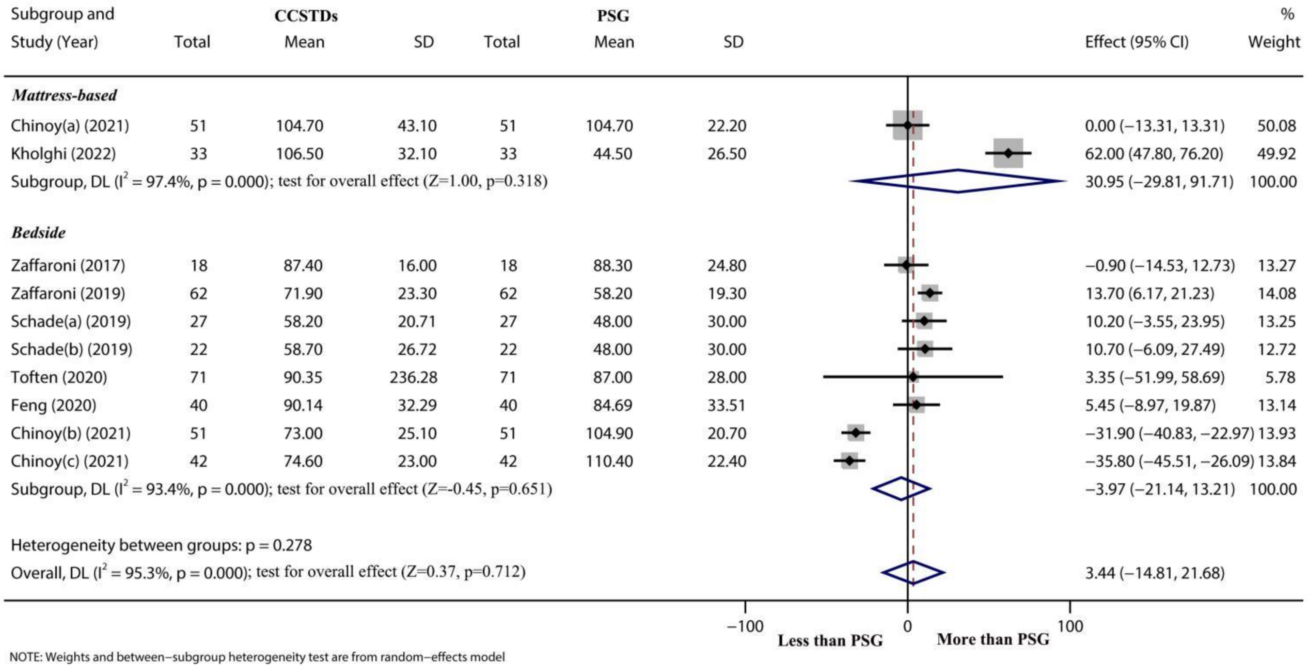

Figure S29. Forest plot meta-analyses for devices type subgroup – REM (min)

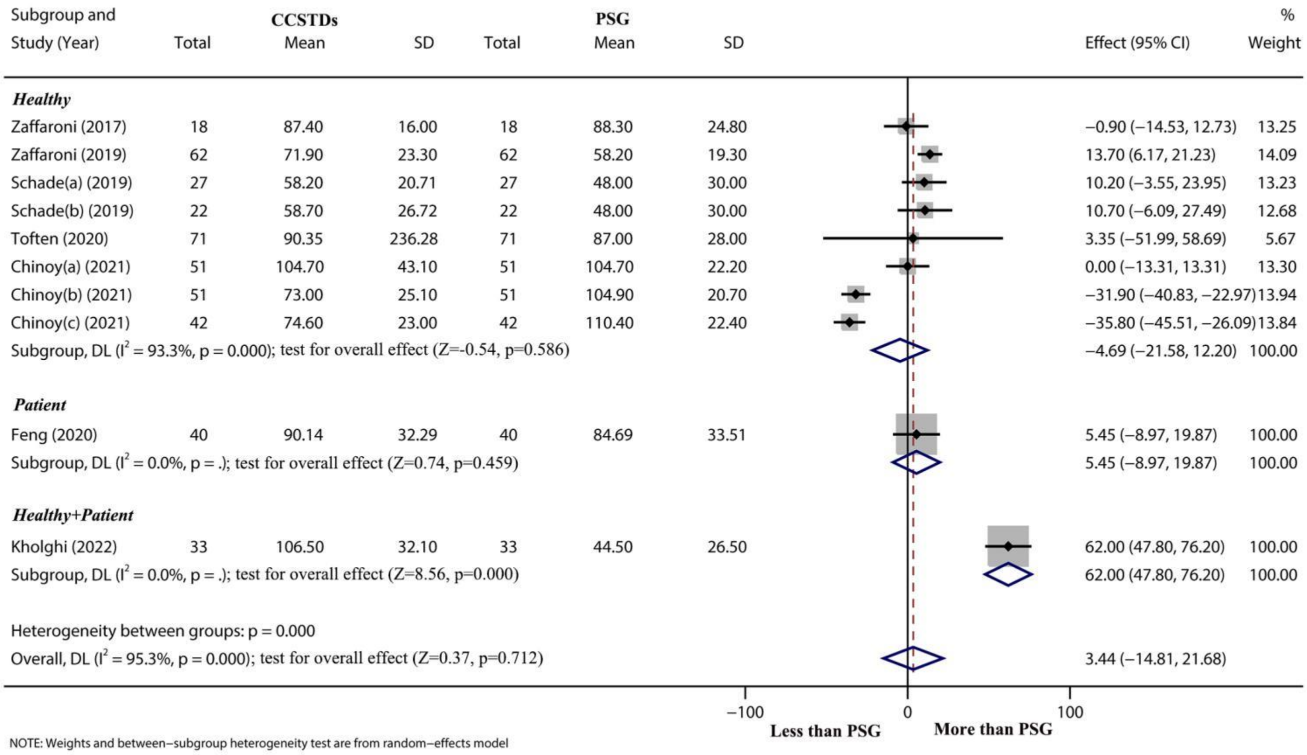

Figure S30. Forest plot meta-analyses for participant types subgroup - REM(min).

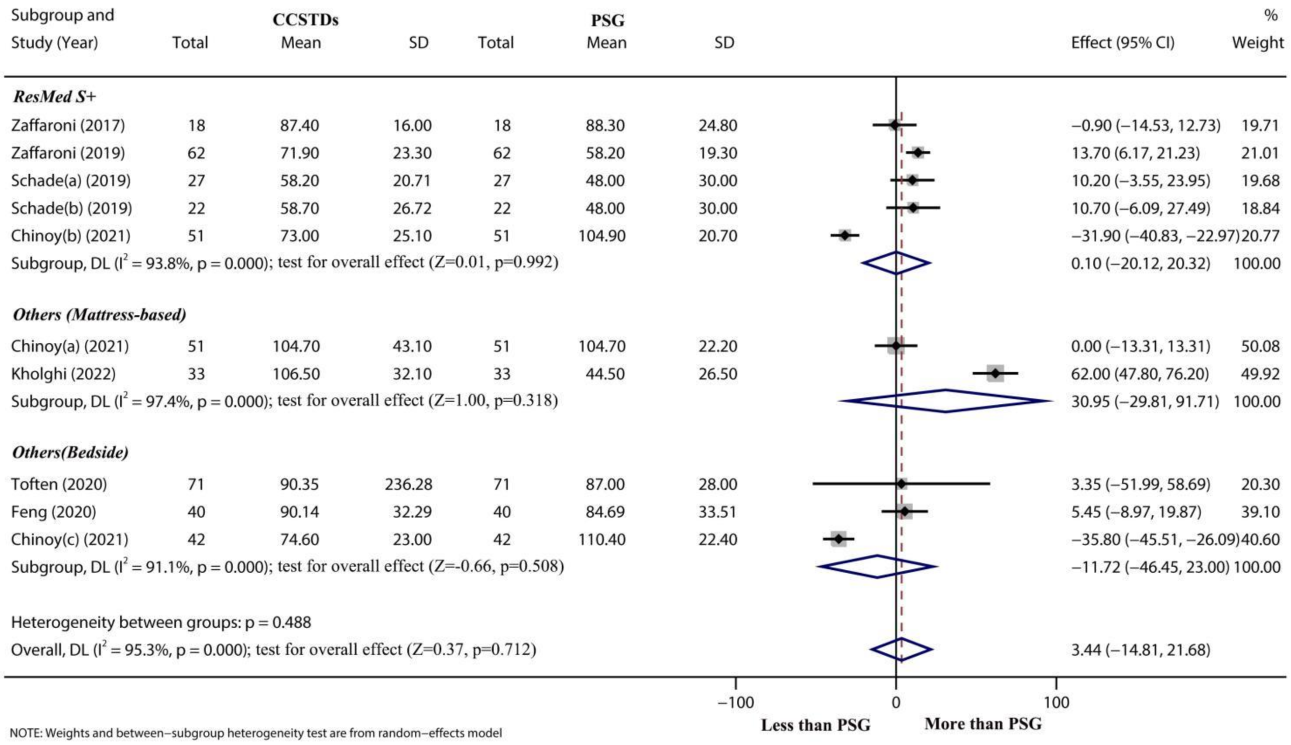

Figure S31. Forest plot meta-analyses for brands subgroup - REM(min).

- REM

The subgroups analyses in Figures S28 to S31 revealed no significant difference compared with PSG except a significant overestimation in pressure sensor (n=1 study; pooled mean =62.00min, 95% CI 47.80 to 76.20; P<.001), and Healthy+Patient (n=1 study; pooled mean =62.00min, 95% CI 47.80 to 76.20; P<.001). Since only one study evaluated piezoelectric sensor, pressure sensor, Patient, and Healthy+Patient, testing for heterogeneity was not relevant, but significant heterogeneity was detected in others.

Reference

1. Zhang Qing-feng, T. Y.-f., She Cui-ping,Zhang Xin-ran,Song Wei,Wang Li-mei,Cheng Chen-jing, Comparative analysis of polysomnography and micro-sensitive mattress-sleep monitor used for obstructive sleep apnea hypopnea syndrome. *Zhonghua er bi yan hou tou jing wai ke za zhi = Chinese journal of otorhinolaryngology head and neck surgery* **2010**, 45(12).
2. De Chazal, P.; Fox, N.; O'Hare, E.; Heneghan, C.; Zaffaroni, A.; Boyle, P.; Smith, S.; O'Connell, C.; McNicholas, W. T., Sleep/wake measurement using a non-contact biomotion sensor. *Journal of sleep research* **2011,** *20* (2), 356-366.
3. Hashizaki, M.; Nakajima, H.; Tsutsumi, M.; Shiga, T.; Chiba, S.; Yagi, T.; Ojima, Y.; Ikegami, A.; Kawabata, M.; Kume, K., Accuracy validation of sleep measurements by a contactless biomotion sensor on subjects with suspected sleep apnea. *Sleep and Biological Rhythms* **2014,** *12* (2), 106-115.
4. O'Hare, E.; Flanagan, D.; Penzel, T.; Garcia, C.; Frohberg, D.; Heneghan, C., A comparison of radio-frequency biomotion sensors and actigraphy versus polysomnography for the assessment of sleep in normal subjects. *Sleep and Breathing* **2014**, 1-8.
5. Pallin, M.; O'Hare, E.; Zaffaroni, A.; Boyle, P.; Fagan, C.; Kent, B.; Heneghan, C.; de Chazal, P.; McNicholas, W. T., Comparison of a novel non-contact biomotion sensor with wrist actigraphy in estimating sleep quality in patients with obstructive sleep apnoea. *Journal of sleep research* **2014,** *23* (4), 475-484.
6. Norman, M. B.; Middleton, S.; Erskine, O.; Middleton, P. G.; Wheatley, J. R.; Sullivan, C. E., Validation of the Sonomat: A contactless monitoring system used for the diagnosis of sleep disordered breathing. *Sleep* **2014,** *37* (9), 1477-1487.
7. Abad, J.; Munoz-Ferrer, A.; Cervantes, M. A.; Esquinas, C.; Marin, A.; Martinez, C.; Morera, J.; Ruiz, J., Automatic Video Analysis for Obstructive Sleep Apnea Diagnosis. *Sleep* **2016,** *39* (8), 1507-15.
8. Terjung, S.; Wang, Y.; Werther, S.; Zaffaroni, A.; Teschler, H.; Weinreich, G., Validation of SleepMinder® for sleep quality evaluation in patients with OSAS. *Somnologie* **2016,** *20* (1), 54-60.
9. Norman, M. B.; Pithers, S. M.; Teng, A. Y.; Waters, K. A.; Sullivan, C. E., Validation of the sonomat against PSG and quantitative measurement of partial upper airway obstruction in children with sleep-disordered breathing. *Sleep* **2017,** *40* (3).
10. Tal, A.; Shinar, Z.; Shaki, D.; Codish, S.; Goldbart, A., Validation of contact-free sleep monitoring device with comparison to polysomnography. *Journal of Clinical Sleep Medicine* **2017,** *13* (3), 517-522.
11. Zaffaroni, A.; Doheny, E. P.; Gahan, L.; Ivanov, Y.; Kilroy, H.; O'Mahony, N.; O'Rourke, D. In *Non-Contact Estimation of Sleep Staging*, Joint Conference of the European

Medical and Biological Engineering Conference (EMBEC) / Nordic-Baltic Conference on Biomedical Engineering and Medical Physics (NBC), Tampere, FINLAND, 2018 Jun; Tampere, FINLAND, 2017; pp 77-80.
12. Chung, K.-Y.; Song, K.; Cho, S. H.; Chang, J.-H., Noncontact Sleep Study Based on an Ensemble of Deep Neural Network and Random Forests. *Ieee Sensors Journal* **2018,** *18* (17), 7315-7324.
13. Zaffaroni A , C. S., Dodd S , et al, Sleep Staging Monitoring Based on Sonar Smartphone Technology. **2019**.
14. Schade, M. M.; Bauer, C. E.; Murray, B. R.; Gahan, L.; Doheny, E. P.; Kilroy, H.; Zaffaroni, A.; Montgomery-Downs, H. E., Sleep validity of a non-contact bedside movement and respiration-sensing device. *Journal of Clinical Sleep Medicine* **2019,** *15* (7), 1051-1061.
15. Tuominen, J.; Peltola, K.; Saaresranta, T.; Valli, K., Sleep parameter assessment accuracy of a consumer home sleep monitoring ballistocardiograph beddit sleep tracker: A validation study. *Journal of Clinical Sleep Medicine* **2019,** *15* (3), 483-487.
16. Miyata, S.; Otake, H.; Ando, M.; Okuda, M.; Fujishiro, H.; Iwamoto, K.; Noda, A.; Sone, M.; Ozaki, N., Patient characteristics affecting accurate detection of sleep apnea using a bed sheet-type portable monitor. *Sleep and Breathing* **2020,** *24* (2), 783-790.
17. Stone, J. D.; Rentz, L. E.; Forsey, J.; Ramadan, J.; Markwald, R. R.; Finomore, V. S., Jr.; Galster, S. M.; Rezai, A.; Hagen, J. A., Evaluations of Commercial Sleep Technologies for Objective Monitoring During Routine Sleeping Conditions. *Nature and Science of Sleep* **2020,** *12*, 821-842.
18. Nagatomo, K.; Masuyama, T.; Iizuka, Y.; Makino, J.; Shiotsuka, J.; Sanui, M., Validity of an under-mattress sensor for objective sleep measurement in critically ill patients: A prospective observational study. *Journal of intensive care* **2020,** *8* (1).
19. Toften, S.; Pallesen, S.; Hrozanova, M.; Moen, F.; Grønli, J., Validation of sleep stage classification using non-contact radar technology and machine learning (Somnofy®). *Sleep medicine* **2020,** *75*, 54-61.
20. Feng, C.; Zhang, H.; Han, Y.; Jin, X.; Yang, F.; Zou, J.; Wang, Y.; Li, Y., Application of impulse-radio ultra-wideband radar as a non-contact portable monitoring device for the diagnosis of obstructive sleep apnea. *Lin chuang er bi yan hou tou jing wai ke za zhi = Journal of clinical otorhinolaryngology, head, and neck surgery* **2020,** *34* (7), 634-638.
21. Chinoy, E. D.; Cuellar, J. A.; Huwa, K. E.; Jameson, J. T.; Watson, C. H.; Bessman, S. C.; Hirsch, D. A.; Cooper, A. D.; Drummond, S. P. A.; Markwald, R. R., Performance of seven consumer sleep-tracking devices compared with polysomnography. *Sleep* **2021,** *44* (5).
22. Edouard, P.; Campo, D.; Bartet, P.; Yang, R. Y.; Bruyneel, M.; Roisman, G.; Escourrou, P., Validation of the Withings Sleep Analyzer, an under-the-mattress device for the detection of moderate-severe sleep apnea syndrome. *Journal of clinical sleep medicine : JCSM : official publication of the American Academy of Sleep Medicine* **2021,** *17* (6), 1217-1227.
23. Ellender, C. M.; Zahir, S. F.; Meaklim, H.; Joyce, R.; Cunnington, D.; Swieca, J., Prospective cohort study to evaluate the accuracy of sleep measurement by consumer-grade smart devices compared with polysomnography in a sleep disorders population. *BMJ open* **2021,** *11* (11).
24. Xue, J. B.; Zhao, R.; Li, J.; Zhao, L.; Zhou, B.; Dong, X. S.; Han, F., [Validation of a contact-free sleep apnea monitor in adults with obstructive sleep apnea]. *Zhonghua jie he he hu xi za zhi = Zhonghua jiehe he huxi zazhi = Chinese journal of tuberculosis and respiratory diseases* **2021,** *44* (10), 880-885.

25. Hsiou, D. A.; Gao, C.; Matlock, R. C.; Scullin, M. K., Validation of a nonwearable device in healthy adults with normal and short sleep durations. *Journal of clinical sleep medicine : JCSM : official publication of the American Academy of Sleep Medicine* **2022,** *18* (3), 751-757.

26. Kholghi, M.; Szollosi, I.; Hollamby, M.; Bradford, D.; Zhang, Q., A validation study of a ballistocardiograph sleep tracker against polysomnography. *Journal of clinical sleep medicine : JCSM : official publication of the American Academy of Sleep Medicine* **2022,** *18* (4), 1203-1210.
